# A platform for SpyCatcher conjugation to native antibodies†

**DOI:** 10.1039/d5sc02286j

**Published:** 2025-05-06

**Authors:** Sona Krajcovicova, Thomas Wharton, Claudia L. Driscoll, Thomas A. King, Mark R. Howarth, David R. Spring

**Affiliations:** a Yusuf Hamied Department of Chemistry University of Cambridge Lensfield Road CB2 1EW Cambridge UK spring@ch.cam.ac.uk; b Department of Organic Chemistry Palacky University Olomouc Tr. 17. Listopadu 12 77900 Olomouc Czech Republic; c Department of Pharmacology University of Cambridge Tennis Court Road CB2 1PD Cambridge UK; d Department of Biochemistry University of Oxford South Parks Road OX1 3QU Oxford UK

## Abstract

Protein–antibody conjugates represent major advancements in targeted therapeutics. However, platforms enabling ‘off-the-shelf’ antibody conjugation are seldom reported. The SpyTag/SpyCatcher system, known for its stable isopeptide bond formation, is widely used to engineer protein architectures and study protein folding. This work introduces the fusion of SpyCatcher with native antibodies using cysteine-reactive tetra-divinylpyrimidine (TetraDVP)-SpyTag linkers. This platform allows for the rapid and stable conjugation of a native antibody with SpyCatcher proteins. As a proof of concept, the HER2-targeting antibody trastuzumab was conjugated to different SpyCatcher proteins using a TetraDVP-SpyTag linker, producing robust conjugates that retained specific binding to HER2-positive cells with excellent conversion rates. To demonstrate the platform's broader applicability, the TetraDVP-SpyTag linker was successfully conjugated to additional native IgG1 and IgG4 antibodies (durvalumab, brentuximab, cetuximab, and gemtuzumab) with similarly high efficiency as trastuzumab. Moreover, a scalable solid-phase synthesis of TetraDVP linkers has been developed, achieving high yields and purity. This innovative platform enables precise, single-step antibody bioconjugation, offering strong potential for protein–antibody conjugate synthesis. With applications across therapeutics and diagnostics, this method advances antibody-based drug development.

## Abstract 2

We introduce a platform for the conjugation of SpyCatcher proteins to native antibodies using cysteine-reactive tetra-divinylpyrimidine (TetraDVP)-SpyTag linkers.

## Graphical abstract

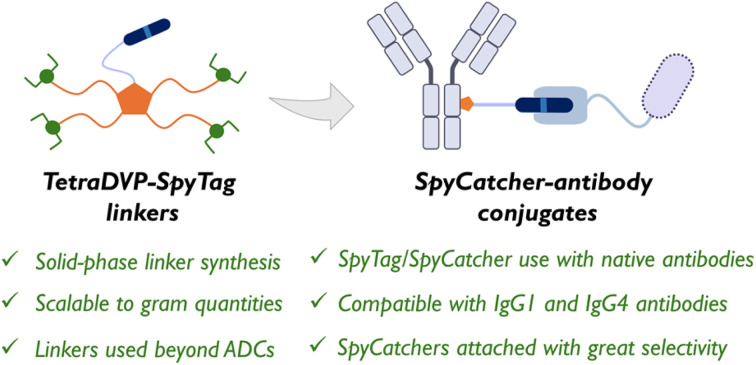

## Introduction

Protein–antibody conjugates represent major advancements in targeted biotherapeutics.^1–4^ Antibodies excel at delivering drugs as antibody–drug conjugates (ADCs) with a high level of selectivity towards a target cell whilst reducing off-target effects and increasing the therapeutic window compared to small molecule therapeutics.^5^ When conjugated with proteins such as enzymes^6,7^ or therapeutic peptides,^8,9^ the resulting constructs can introduce new functionalities that are not typically available through small molecule drug conjugates. These functionalities may include enzymatic activity, targeting protein–protein interactions, or fluorescent labelling for imaging applications. The ability to precisely target disease-related biomarkers, coupled with the versatility of protein payloads, makes protein–antibody conjugates an attractive tool in modern biomedicine.^4^ However, platforms for ‘off-the-shelf’ antibody conjugation to various proteins of interest are seldom reported.^10^ Such a platform would enable direct coupling of native antibodies to target proteins, avoiding the extra cost and time-consuming engineering required for recombinant antibodies.

To address the need for more precise and versatile protein-antibody conjugation strategies, we utilised the SpyTag/SpyCatcher system – a powerful tool for irreversible peptide–protein ligation, widely used for binding, labelling, immobilisation, and building of novel protein architectures.^7,11–14^ The SpyTag/SpyCatcher system is based on the spontaneous formation of a covalent isopeptide bond between lysine (Lys31) and aspartic acid (Asp117) residues within the CnaB2 domain of *Streptococcus pyogenes*. This reaction, catalysed by a nearby glutamate (Glu77), is highly stable. The two components – SpyCatcher (15 kDa) and SpyTag (13 residues) – can be expressed or chemically synthesised separately and, upon mixing, rapidly form stable conjugates with high specificity.^14^

**Fig. 1:**
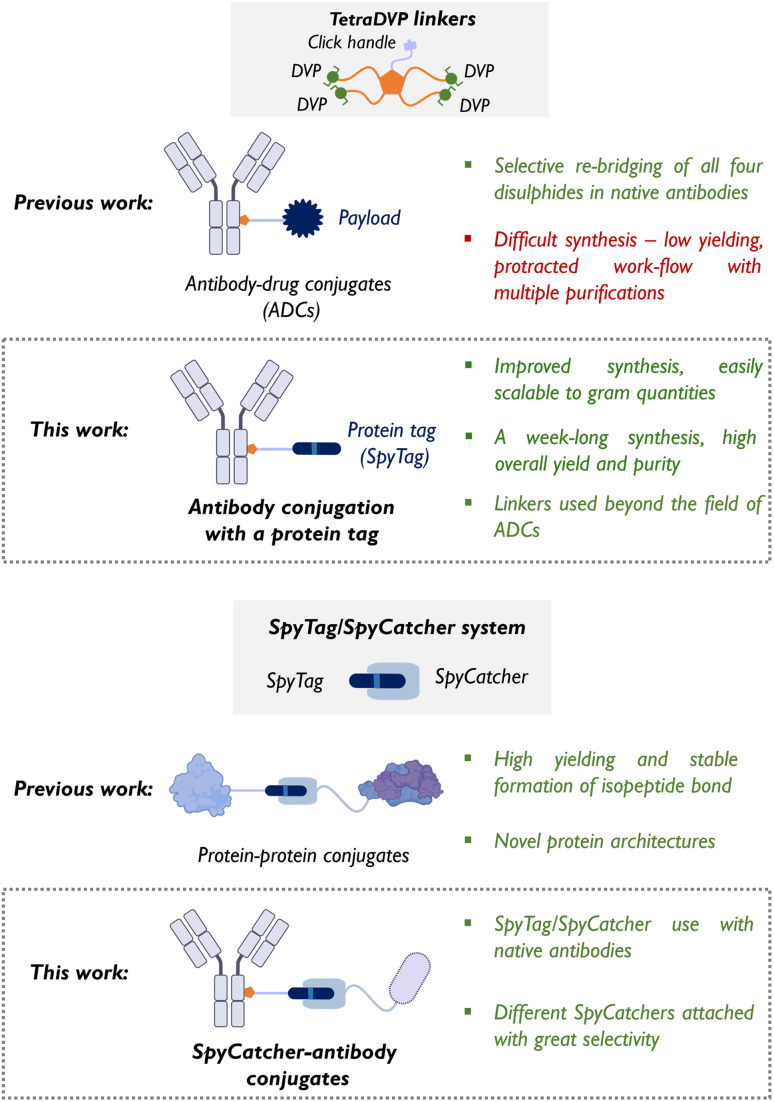
TetraDVP linkers and SpyTag/SpyCatcher system – then and now (size of the cartoons is purely schematic).

In this work, we integrated the SpyTag/SpyCatcher system with tetra-divinylpyrimidine (TetraDVP) linkers to generate stable SpyCatcher-antibody conjugates, representing an advancement in antibody conjugation technology (Fig. 1). Several well-established strategies have been developed for site-selective disulphide modification of native antibodies, including next-generation maleimides,^15–17^ pyridazinediones,^18–22^ pyrimidine nitriles,^23^ and phosphonamidates,^24–27^ among others.^28^ TetraDVP linkers are a class of cysteine-reactive scaffolds capable of simultaneously re-bridging all four interchain disulphide bonds in native antibodies using a single construct, thereby enabling site-specific conjugation with a defined payload-to-antibody ratio (PAR).^29,30^ TetraDVP linkers enable efficient conjugation and generate highly stable antibody constructs, making them a versatile platform that extends beyond classical ADCs to more complex protein–antibody conjugates.

However, the widespread adoption of TetraDVP linkers has been limited by their synthesis with low yields, challenging purification, and time-consuming procedures that hinder their scalability.^29,30^ To overcome these challenges, we herein developed a solid-phase organic synthesis (SPOS) approach, significantly improving the yield, efficiency, and purity of TetraDVP linkers. This scalable method addresses the limitations of conventional solution-phase synthesis, reducing production times and enhancing reproducibility. For the first time, the SpyTag peptide has been chemically modified and conjugated with TetraDVP linkers, creating a flexible platform for generating SpyCatcher-antibody conjugates. This approach facilitates the production of protein–antibody conjugates in a single step, offering broad applications in both therapeutic and diagnostic fields. By extending the scope of antibody conjugation beyond cytotoxic payloads, this platform holds significant potential to accelerate the development of next-generation antibody-based therapeutics.

## Results and discussion

### Design and synthesis of TetraDVP-SpyTag linkers

Several drawbacks in the previously designed TetraDVP linkers were identified, which, if overcome, could make the overall synthesis more efficient. For instance, the gradual polyethylene (PEG) chain elongation posed significant challenges due to high hydrophilicity and aggregation in organic solvents, complicating isolation and resulting in low yields with standard techniques like liquid–liquid extraction and silica gel chromatography.^30^ In contrast, SPOS allows smooth incorporation of PEG chains into TetraDVPs with quantitative conversions and high crude purity after cleavage, eliminating the need for intermediate purification. By retaining resin-bound intermediates and filtering out excess reagents, SPOS simplifies synthesis and accelerates production. This method is particularly effective for substrates with solubility issues or hazardous properties.^31–33^ It reduces hands-on time and minimises purification steps while requiring only basic equipment, making it highly accessible to most synthetic laboratories.

**Scheme 1:**
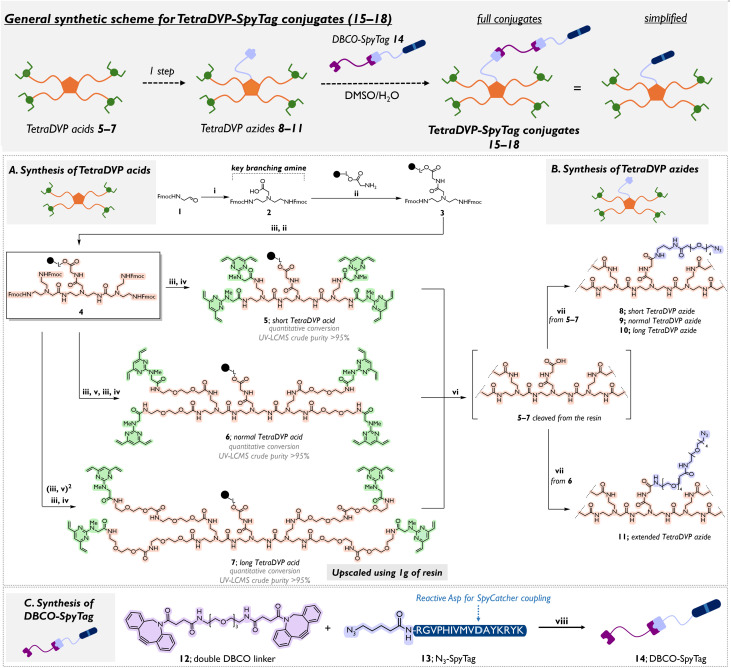
Synthesis of TetraDVP-SpyTag conjugates 15–18. Full structures can be found in the ESI.† Black ball indicates 2-chlorotrityl chloride polystyrene resin. Reaction conditions: (i) glycine, Bu_2_SnCl_2_, PhSiH_3_, THF, reflux, 16 h (98%, 2); (ii) H-Gly-2CT resin, key branching amine 2, HOBt, DIC, r.t., 16 h; (iii) DBU/CH_2_Cl_2_ 5 : 95, r.t., 15 min; (iv) sarcosine DVP S1, HOBt, DIC, DMF, r.t., 16 h; (v) {2-[2-(Fmoc-amino)ethoxy]ethoxy}acetic acid (FAEEAA), HOBt, DIC, DMF, r.t., 16 h; (vi) HFIP/CH_2_Cl_2_ 1 : 4, r.t., 3 h; (vii) for 8–10: TetraDVP acids 5–7 (respectively), azido spacer S4, HOBt, DIC, DMSO, r.t., 16 h; for 11: TetraDVP acid 6, azido spacer S6, HOBt, DIC, DMSO, r.t., 16 h. (viii) DMSO, r.t., 1 h (94%, 14).

Different TetraDVP linker lengths were evaluated to determine the optimal linker for achieving quantitative antibody conjugation, addressing previous challenges with conversion efficiency. Synthesis of TetraDVP acids 5–7 began with commercially available 2-chlorotrityl chloride (2-CT) resin preloaded with H-Gly-OH, followed by amide coupling with the key branching amine 2. The 2-CT resin was selected to ensure mild cleavage conditions with hexafluoroisopropanol (HFIP), avoiding possible degradation of the DVP core under strong acid, such as TFA. The synthesis of 2 was streamlined from a five-step process involving multiple extractions and purifications^30^ to a single high-yielding step using tin-mediated silane-based reductive amination^34,35^ of glycine with commercially available (9*H*-fluoren-9-yl)methyl (2-oxoethyl)carbamate 1 (Scheme 1A). Deprotection of Fmoc from intermediate 3 with 1,8-diazabicyclo[5.4.0]undec-7-ene (DBU) in CH_2_Cl_2_, followed by a second amide coupling with 2, produced the core tetra-Fmoc intermediate 4. Sequential deprotection and coupling with {2-[2-(Fmoc-amino)ethoxy]ethoxy}acetic acid (FAEEAA) generated normal-length (S12) and long-length (S13) Tetra-PEG intermediates (see ESI Section 1.3† for structures). TetraDVP acids 5–7 were then obtained by coupling sarcosine-based DVP S1 to the resins, completing the synthesis in 5–9 steps in a one week time frame with no intermediate purification and excellent purity after cleavage from the resin. The modularity of the SPOS method allowed us also to easily adjust the length of the azido-containing spacers, enabling their fine-tuning for optimal bioconjugation. Azido spacers S4 and S6 were amide coupled to TetraDVP acids 5–7 post-cleavage from the resin by HFIP, yielding TetraDVP azides 8–11 of varying lengths (Scheme 1B). This modular SPOS approach enabled the first scalable synthesis of TetraDVP acid 7, affording gram-scale quantities in one week with an 83% overall yield. Unlike traditional solution-phase methods, it allows rapid linker diversification without re-synthesis, supporting industrial-scale applications requiring high-purity linkers.

To prepare the desired SpyCatcher-antibody conjugates, TetraDVP-SpyTag compounds 15–18 were synthesised. The SpyTag peptide was prepared using automated solid-phase peptide synthesis and, after terminal Fmoc deprotection, reacted with 5-azidopentanoic acid to yield 13. A strain-promoted azide–alkyne cycloaddition (SPAAC) between 13 and double-DBCO linker 12 produced 14 in quantitative yield within one hour. TetraDVP azides 8–11 were then reacted with DBCO-SpyTag 14 in a DMSO/H_2_O mixture at ambient temperature, yielding four final TetraDVP-SpyTag conjugates – short TetraDVP-SpyTag 15, normal TetraDVP-SpyTag 16, long TetraDVP-SpyTag 17 and extended TetraDVP-SpyTag 18 (Scheme 1 General synthetic scheme; see ESI Section 1.3† for full structures).

### Optimisation of TetraDVP-SpyTag bioconjugation with trastuzumab

**Fig. 2:**
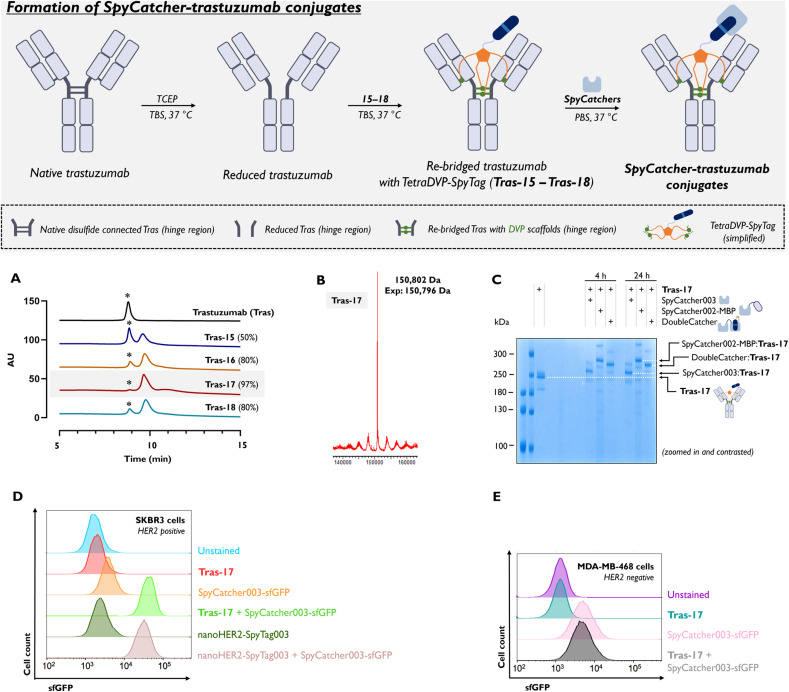
Formation of SpyCatcher-trastuzumab conjugates. The SpyTag-trastuzumab conjugates Tras-15 to Tras-18 originate from the conjugation of reduced trastuzumab with TetraDVP-SpyTag conjugate 15 for Tras-15, 16 for Tras-16, 17 for Tras-17, and 18 for Tras-18 (the full structures of the TetraDVP-SpyTag conjugates are available in the ESI Section 1.3†). Conjugations run in TBS (1×, 2.5 mg per mL trastuzumab, 100 μL) with 10 eq. TCEP for 1 h then 5 eq. of TetraDVP conjugates 15, 16, 17, or 18 were added and incubated at 37 °C for 23 h; repeated daily for 3 days. (A) Hydrophobic interaction chromatography (HIC) analysis of trastuzumab-TetraDVP-SpyTag conjugates. Percentage shows conversion from native trastuzumab (*). (B) Deconvoluted MS data of the most successful conjugate Tras-17; intensity *vs.* deconvoluted mass. (C) Conjugation of Tras-17 to different SpyCatcher constructs. 1 μM Tras-17 was incubated with 5 μM of either SpyCatcher003, SpyCatcher002 linked to maltose-binding protein (SpyCatcher002-MBP), or DoubleCatcher, in PBS pH 7.4 at 37 °C for the time indicated. Reactivity of Tras-17 with each Catcher was monitored by SDS-PAGE analysis followed by Coomassie staining (first two lanes are broad range unstained protein standard, molecular weight ladders). Cartoons represent protein conjugation partners, with SpyCatcher003 in light blue, MBP in lilac, SpyTag003 in dark blue with D117A indicated in light blue in DoubleCatcher, TEV site in orange. (D) Tras-17 pre-conjugated to SpyCatcher003-sfGFP (2 μM) was incubated with HER2-positive SKBR3 cells, or (E) HER2-negative MDA-MB-468 cells in HBS pH 7.2 + 10% (v/v) FBS for 30 min at 37 °C with 5% (v/v) CO_2_ before detection of sfGFP fluorescence by flow cytometry. As a positive control for HER2 binding, the anti-HER2 nanobody nanoHER2 was used instead of Tras-17. Cells incubated with either HER2-binder alone, SpyCatcher003-sfGFP alone, or buffer only were used to detect background signal. Abbreviations: sfGFP = superfolder Green Fluorescent Protein; HER2 = Human Epidermal Growth Factor Receptor 2; TCEP = Tris(2-carboxyethyl)phosphine; Tras = Trastuzumab (marked with asterisk *).

In our previous studies on ADCs construction,^30^ TetraDVP azides generally showed moderate side reactivity with tris(2-carboxyethyl)phosphine (TCEP). This is likely due to an undesired Staudinger reaction of the azide to amine which therefore hindered complete drug attachment *via* ‘click’ chemistry. To avoid this and prevent yield loss, we opted not to conjugate them to trastuzumab before the DBCO-SpyTag click reaction. Instead, bioconjugation was performed exclusively with pre-assembled TetraDVP-SpyTag constructs 15–18. Initial tests showed that the long TetraDVP-SpyTag 17 achieved the highest bioconjugation efficiency, with hydrophobic interaction chromatography (HIC) showing 97% conversion to Tras-17 (Trastuzumab-TetraDVP-SpyTag; Fig. 2A). In comparison, normal TetraDVP-SpyTag 16 reached 80%, while short TetraDVP-SpyTag 15 achieved only 50% bioconjugation efficiency, indicating that a longer PEG core enhances the conjugation. No solubility issues were observed for any of the linkers. Notably, TetraDVP-SpyTag 18, with an extended tether, showed similar bioconjugation efficiency to 16 (80%), suggesting that the distance from the SpyTag to the core is less restrictive than the distance from the DVP core (Fig. 2A).

**Fig. 3:**
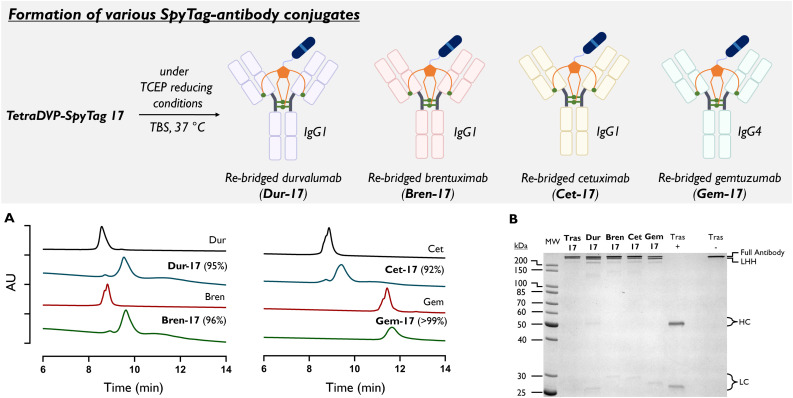
Formation of antibody-SpyTag conjugates Dur-17, Bren-17, Cet-17 (IgG1), and Gem-17 (IgG4). Bioconjugation of IgG1 antibodies: conjugations run in TBS (1×, 2.5 mg per mL trastuzumab, 100 μL) with 10 eq. TCEP for 1 h then 5 eq. of TetraDVP conjugate 17 was added and incubated at 37 °C for 23 h. Conjugation of gemtuzumab (IgG4) with 17 required three daily reagent additions to reach completion. (A) Hydrophobic interaction chromatography (HIC) analysis of Dur-17, Bren-17, Cet-17, and Gem-17. Percentage shows conversion from native antibody. (B) SDS-PAGE gel of Dur-17, Bren-17, Cet-17 and Gem-17 with Coomassie blue staining. +/− indicates with or without reducing stain. TCEP = Tris(2-carboxyethyl)phosphine, TBS = Tris Buffered Saline.

Due to incomplete conjugation, initial tests were conducted with a gradual addition of TetraDVP-SpyTag linkers to trastuzumab over 72 hours to achieve maximum conversion. With the aim to enhance the bioconjugation rate, the best candidate (TetraDVP-SpyTag 17) was re-purified using HPLC and underwent additional filtration prior to purification. Pleasingly, this new batch allowed for 92% conversion after a single 24 hours incubation with reduced trastuzumab, therefore all further studies were carried out over 24 hours. As was seen in our previous studies,^29,30^ the conjugation leads to two high molecular weight bands on SDS-PAGE gels (Fig. 3B) which MS analysis shows are the desired full re-bridged antibody and re-bridged light-heavy-heavy (LHH) chain antibody, with one light chain not covalently attached (Fig. S1†). Analysis of MS data showed that the re-bridged LHH species has a mass 250 Da higher than expected; this was attributed to the addition of a TCEP molecule (250 Da) to one of the vinyl groups of the TetraDVP core, presumably *via* conjugate addition. Consequently, once TCEP reacts with a vinyl group, the TetraDVP linker can no longer re-bridge to the final light chain, leading to the formation of the LHH species. Despite our best efforts, we were unable to completely eliminate its occurrence.§§Removal of TCEP *via* a desalting column prior to the addition of TetraDVP 17 was attempted but led to poor conversion due to the disulphide bond reoxidisation competing with the bioconjugation. This reactivity is well-documented in other cysteine-reactive bioconjugation platforms.^36^

Fortunately, as has been shown previously,^16,37^ activity of an antibody conjugate is maintained despite missing interchain covalent bonds. In this case, non-covalent interactions to the second light chain will afford the LHH species similar properties to the fully re-bridged antibody, with both species PAR 1. Therefore, whilst the aim of a truly homogenous PAR 1 antibody conjugation method continues to be elusive, the TetraDVP platform remains the only method to access PAR 1 species from native antibodies without the need for chromatographic purification.^38^

### Biological validation and target binding of SpyCatcher-Trastuzumab conjugates

The optimised Tras-17 conjugate was then further analysed for its biological function and ability to bind to SpyCatcher partners. As a proof-of-concept, we sought to validate the reactivity of Tras-17 with three SpyCatcher-containing proteins, which each exemplify a unique application of SpyTag-conjugated IgG molecules: SpyCatcher003 alone (15.6 kDa), which may be site-specifically conjugated to fluorescent dyes for imaging;^13^ SpyCatcher002-MBP (56.0 kDa), to demonstrate the coupling of an IgG to a protein payload or enzyme;^11^ or DoubleCatcher (31.1 kDa), a tandem SpyCatcher003 scaffold optimised for the heterodimerisation of binder moieties, for the synthesis of full-length IgG-containing bispecific antibodies.^39^Tras-17 was mixed with a five-fold molar excess of each SpyCatcher-containing construct in PBS at 37 °C for 4 or 24 hours, and isopeptide bond reconstitution (between SpyTag-Asp10 and SpyCatcher-Lys31) was monitored by SDS-PAGE with Coomassie staining (Fig. 2C). A shift in molecular weight for the major band was observed after incubation with each of the three constructs, with no unreacted Tras-17 visible after 4 hours. The molecular weight shift of the two minor bands within the sample upon incubation with each SpyCatcher construct corroborates the finding from the LCMS data for Tras-17 that there is no presence of any unmodified trastuzumab in the conjugated product (Fig. S1†).

To assess whether the trastuzumab retained tight and specific binding activity to HER2 after conjugation to SpyTag, we then conducted flow cytometric analysis of Tras-17 on the HER2-positive breast cancer cell line SKBR3 and the HER2-negative breast cancer cell line MDA-MB-468 (Fig. 2D and E). Tras-17 was pre-reacted with SpyCatcher003-sfGFP to detect binding to cells by sfGFP fluorescence. This additionally ensures that binding of SpyCatcher-coupled Tras-17 is monitored, mimicking the intended format as a therapeutic or detection tool. The anti-HER2 nanobody nanoHER2-SpyTag003 fusion, which we have previously validated to bind tightly and specifically to the HER2 extracellular domain,^39,40^ was used as a positive control for binding. We found Tras-17 bound with high affinity to SKBR3 cells, with more than a log-fold shift in fluorescence over background from the SpyCatcher003-sfGFP alone control, which was greater than the shift in signal for the nanoHER2-SpyTag003 positive control (Fig. 2D). Specificity of Tras-17 binding to HER2 was confirmed by the absence of shift in signal on Tras-17-SpyCatcher003-sfGFP-treated MDA-MB-468 cells over MDA-MB-468 cells treated with SpyCatcher003-sfGFP alone (Fig. 2E).

### Beyond trastuzumab: TetraDVP-SpyTag conjugation to different native IgG1 and IgG4 antibodies

As we wanted to extend the use of our TetraDVP linker methodology to different antibodies, conjugation to several clinically approved IgG1 and IgG4 antibodies was trialled. The use of IgG4 antibodies is of particular interest for general ADC research, because the Fc region binding affinity of an IgG4 antibody is often lower than an IgG1, lowering the potency of the antibody component itself at triggering an immune system response.^41^ This can be preferable as the antibody component will cause less overall cytotoxicity, thus increasing the safety profile of the ADC. An issue with IgG4 antibodies, however, is that they exhibit the ability to fragment in the hinge region to release two half-antibody fragments.^42^ These can then recombine with different IgG4 half-antibodies to create random bispecifics (*i.e.* antibody scrambling). The use of TetraDVP removes this issue, re-bridging all four disulphides with a single construct, preventing the fragmentation of the antibody. This could then allow for the study of single-payload species using IgG4 antibodies for a desired target with the benefit of the reduced immune response and removal of the antibody scrambling.

To explore the antibody tolerance of the TetraDVP bioconjugation, TetraDVP-SpyTag 17 was conjugated to the IgG1 antibodies durvalumab, brentuximab, and cetuximab (generating Dur-17, Bren-17, and Cet-17), and the IgG4 antibody gemtuzumab (Gem-17; Fig. 3). Pleasingly, the bioconjugations to the IgG1 antibodies were analogous to that of trastuzumab, leading to >92% conversion to PAR 1 species in all cases (Fig. 3A). The conjugation to gemtuzumab proved slower, requiring extra equivalents of TCEP and 17 to be added over 72 hours to reach high conversion (Fig. S7†). It is postulated that this is due to the differing spatial arrangement of the disulphide bridges in IgG4 antibodies compared to IgG1.

Nevertheless, to the best of our knowledge this represents the first IgG4 PAR 1 conjugate synthesised. The use of TetraDVP scaffolds on IgG4 antibodies should therefore enable stable DAR 1 species to be produced for the first time, removing the possibility of antibody scrambling.

## Conclusion

In summary, this work introduces a novel approach for the synthesis of TetraDVP linkers that enable precise incorporation of the SpyTag/SpyCatcher system into native antibodies, forming protein–antibody conjugates. For the first time, solid-phase organic synthesis (SPOS) has been employed for the preparation of these linkers, demonstrating notable advantages, including elevated yields, parallel synthesis, exceptional crude purities and minimal hands-on time to prepare the desired conjugates. The method enabled the efficient and scalable synthesis of TetraDVP acids 5–7, with the gram-scale preparation of TetraDVP acid 7 demonstrating the practicality of the approach for larger-scale applications. These results represent a dramatic improvement in the synthesis of TetraDVP linkers. Following optimisation of the bioconjugation, a single TetraDVP-SpyTag construct, 17, simultaneously re-bridged all four interchain disulphides of the antibody trastuzumab in a one-step reaction, enabling 92% conversion to single payload Trastuzumab-TetraDVP-SpyTag Tras-17, showing the potential of this method. Biological functionality of Tras-17 was evaluated, showing a complete molecular weight shift of Tras-17 after incubation with three SpyCatcher-containing protein constructs, confirming that the TetraDVP-conjugated SpyTag is still functional and reactive with SpyCatcher even after conjugation to trastuzumab. This further demonstrated the applicability of Tras-17 as an IgG1 anchor for protein payloads. Pleasingly, flow cytometric analysis of Tras-17 with HER2-positive cells or HER2-negative cells revealed that strong and specific binding of trastuzumab to HER2 was retained after bioconjugation with 17. Together, these results confirm that the biological activities of both SpyTag and trastuzumab are not perturbed upon their bioconjugation *via* the TetraDVP linkers, supporting this platform as an attractive strategy for the development of protein–antibody conjugates as improved therapeutics or diagnostic tools.

Importantly, TetraDVP-SpyTag species 17 conjugated efficiently to a range of native IgG1 antibodies and, for the first time, with a native IgG4 antibody. The data obtained underscores the desirability and utility of solid-phase synthesis offering a rapid and efficient platform for the preparation of novel antibody conjugates *via* one-step disulphide re-bridging.

## Data availability

The data supporting this article have been included as part of the ESI.†

## Author contributions

S. K. and T. W. were involved in conceptualisation, data curation, formal analysis, investigation, methodology, validation of the chemical and bioconjugation parts, visualisation, and writing (original draft and editing). C. L. D. did formal analysis, data curation, validation of SpyTag biological results and writing of the biological part (original draft and editing). T. K. was involved in initial investigation. M. R. H. and D. R. S. were involved in conceptualisation, funding acquisition, supervision and writing (editing). All authors have been involved in proof-reading of the manuscript.

## Conflicts of interest

There are no conflicts to declare.

## Supplementary Material

SC-OLF-D5SC02286J-s001
