# Supplementary material for: A platform for SpyCatcher conjugation to native antibodies
Source: Chem Sci. 2025 May 6;16(23):10602–9. doi: 10.1039/d5sc02286j (PMC12080404; doi:10.1039/d5sc02286j)
Supplement: SC-016-D5SC02286J-s001 [file SC-016-D5SC02286J-s001.pdf]

Supplementary Information (SI) for Chemical Science.
This journal is © The Royal Society of Chemistry 2025

This version of the ESI published 09/05/2025 replaces the previous version published 06/05/2025. This version includes a reference list.

Supporting Information

A Platform for SpyCatcher Conjugation to Native Antibodies

Sona Krajcovicova, Thomas Wharton, Claudia L. Driscoll, Thomas A. King, Mark R. Howarth and David R. Spring

Table of Contents

1. Experimental Procedures ... 3
1.1. General Information ... 3
1.2. Instrumentation ... 4
1.3. Manuscript related structures ... 6
1.4. Experimental Procedures ... 7
1.4.1. General procedure A: Amide coupling on resin ... 7
1.4.2. General procedure B: Manual Fmoc deprotection ... 7
1.4.3. General procedure C: Amide coupling in solution ... 7
1.4.4. General procedure D: SPAAC reaction ... 7
1.4.5. Key branching amine **2** ... 7
1.4.6. DVP **S1** ... 8
1.4.7. Azido spacer **S4** ... 8
1.4.8. Azido spacer **S6** ... 9
1.4.9. Double DBCO linker **12** ... 10
1.4.10. SpyTag-azide **13** ... 10
1.4.11. DBCO-SpyTag **14** ... 11
2. Synthesis of TetraDVP linkers ... 11
2.1. Synthesis of TetraFmoc compound **4** ... 11
2.2. Synthesis of Tetra-Fmoc-PEG compounds **S12** and **S13** ... 12
2.3. Synthesis of TetraDVP acids ... 13
2.4. Synthesis of TetraDVP azides ... 15
2.5. Synthesis of final TetraDVP-SpyTag conjugates ... 17
3. Analytical HPLC spectra ... 19
4. Bioconjugation ... 26
4.1. General bioconjugation procedure ... 26
4.2. Final conjugates bioconjugation ... 26
4.3. **Tras-17** conjugate analysis ... 27
4.4. **Dur-17** conjugate analysis ... 28
4.5. **Bren-17** conjugate analysis ... 29
4.6. **Cet-17** conjugate analysis ... 30
4.7. **Gem-17** conjugate analysis ... 31
4.8. Bioconjugation optimisation ... 32
5. Biology ... 34
6. NMR spectra ... 36
7. References ... 45

1. Experimental Procedures

1.1. General Information

All reagents were of reagent grade and used without further purification. Solvents and chemicals were purchased from Sigma-Aldrich (US), Acros Organics (BE), Fluorochem (UK) or Iris Biotech (DE). Anhydrous solvents were stored as received from commercial suppliers. Software used: chemical structures (ChemDraw), NMR (MestReNova and TopSpin), UV-LCMS (MassLynx), analytical HPLC/HIC/SEC (Prism), figures (MS PowerPoint), citations (Zotero).

Reactions in solution were performed in round-bottom flasks fitted with rubber septa under positive pressure of nitrogen, in ace-pressure tubes (up to 10 mL) or in conical vials (up to 4 mL). All reactions were monitored by UV-LCMS analysis. Thin-layer chromatography (TLC) plates using aluminium plates precoated with silica gel (silica gel 60 F254, Merck, US) impregnated with a fluorescent indicator were visualised by exposure to ultraviolet light (λ = 254 nm). Column chromatography was performed using silica gel (60 Å, 230–400 mesh, Sigma-Aldrich).

Automated peptide synthesis and automated solid-phase synthesis of TetraDVP linkers was carried out on a CEM Liberty Automated Microwave Peptide Synthesiser using Rink Amide resin (loading 0.3–0.8 mmol/g) or 2-Cl-Trt resin preloaded with H-Gly-OH (loading 0.85 mmol/g) or H-Ala-OH (0.59 mmol/g). The scale for resin-bound compounds was 0.25 mmol. All automated amide couplings were performed with Fmoc-protected amino acids (0.2 M), FAEEAA (0.15 M), **2** (0.15 M), Oxyma pure (1 M) and DIC (1 M) as coupling reagents in DMF. After automated synthesis all resin-bound compounds were transferred to plastic syringes each equipped with a porous disk, washed with CH_2Cl_2 (5 × 10 mL), DMF (2 × 10 mL) and CH_2Cl_2 again (2 × 10 mL) and stored dried in a freezer. Fmoc deprotection was performed with 20% piperidine in DMF. Protecting groups used during solid-phase peptide synthesis: *t*-Bu (Asp, Tyr); Boc (Lys, His); Pbf (Arg).

For manual solid-phase synthesis plastic syringes were used, each equipped with a porous disk using 2-chlorotrityl chloride resin pre-modified with H-Gly-OH (loading 0.66 mmol/g). All external reagents were dissolved in a glass beaker in an appropriate solvent and added to the resin at once, unless stated otherwise. The volume of used solvent was 10 mL per 1 g of resin, unless stated otherwise. All manual solid-phase reactions were performed at ambient temperature, unless stated otherwise. For manual washing, resin slurry was shaken with the fresh solvent for at least 1 min before changing the solvent. UV-LCMS analysis was performed after each reaction step (except for Fmoc cleavage) to ensure completeness of the synthesis, unless stated otherwise.

Upon solid-phase synthesis, UV-LCMS analysis was performed as follows: analytical sample of a resin (~5 mg) was transferred into a plastic Eppendorf tube, treated with a cleavage cocktail (**Rink amide:** CH_2Cl_2/TFA 1:1, 1 mL, v/v; **2-Cl-Trt:** CH_2Cl_2/HFIP 2:1, 1.5 mL, v/v) and shaken for 1–3 hours at ambient temperature. For peptides containing trityl protecting group, 100 µL of TIPS was added to a cleavage cocktail with TFA as a quencher. The cleavage cocktails were then evaporated under a stream of nitrogen, cleaved compounds extracted into DMSO, filtered, and submitted to UV-LCMS analysis to determine purity of the prepared compounds.

For full cleavage of TetraDVP linkers from the resin a cleavage cocktail of CH_2Cl_2/HFIP (2:1, 20 mL, v/v) was used and the cleavage time was prolonged to 4–5 hours (usually 2 × 2-hour cycles were performed). Cleavage cocktails were evaporated under a stream of nitrogen, crude products were suspended in DMSO to obtain a homogeneous mixture and purified by preparative HPLC. For the full cleavage of peptides from the resin prior to HPLC purification a cleavage cocktail of CH_2Cl_2/TFA/H2O (1:4:0.5 v/v, 30 mL) was used.

1.2. Instrumentation

UV-LCMS analysis was carried out using Waters ACQUITY H-Class UPLC with an ESCi Multi-Mode Ionisation Waters SQ Detector 2 spectrometer using ACQUITY UPLC® CSH C18 (2.1 mm × 50 mm, 1.7 μM, 130 Å) column at 40 °C and flow rate 0.6 mL/min. Mobile phase was (A) 2 mM ammonium acetate in water/MeCN (95:5), (B) MeCN and (C) 2% aq. formic acid, linearly programmed from 5–95% B over 5 min with constant 5% C over 1 min. The ESI source operated at a discharge current of 5 μA, vaporiser temperature of 350 °C and capillary temperature of 200 °C. Chromatographs were monitored by UV absorbance using a photodiode array detector at a wavelength range of 210–800 nm, interval 1.2 nm.

Preparative HPLC was carried out on an Agilent 1260 Infinity using a Supelcosil ABZ+PLUS column (250 mm × 21.2 mm, 5 μM) eluting with a linear gradient system with mobile phases (A) 0.1% TFA in water (v/v) and (B) 0.05% TFA in MeCN (v/v) over 20 min at a flow rate of 20 mL/min.

Preparative UV-LCMS-HPLC was carried out on Waters AutoPurification mass-directed HPLC system with photodiode array detector, SQ Detector 2 and at-column dilution. The analytical runs used an X-Select CSH C18 column (4.6 × 150mm, 5 μM particle size) and flow rate of 1.2 mL/min. The preparative runs used an X-Select CSH Prep C18 OBD column (19 × 150mm, 5 μM particle size) and flow rate of 20 mL/min. The machine used a linear gradient of solvent B in solvent A run over 15 min with 1 min isocratic hold before the gradient (analytical) or over 15 min with 1.4 min isocratic hold (preparative). For acidic runs, solvent A was water with 0.1% (v/v) formic acid and solvent B was MeCN with 0.1% (v/v) formic acid. For neutral runs solvent A was water (no additives) and solvent B was MeCN (no additives).

Analytical HPLC was carried out using an Agilent 1260 Infinity system with a reversed-phase Supelcosil™ ABZ+PLUS column (150 mm × 4.6 mm, 3 μM) eluting with a linear gradient system (solvent A: 0.05% (v/v) TFA in water, solvent B: 0.05% (v/v) TFA in MeCN) over 18 minutes, at a flow rate of 1 mL/min. Analytical HPLC was monitored by UV absorbance at 254 and 280 nm.

NMR spectra were recorded on Bruker Avance III 500 MHz HD Smart Probe and Bruker TXO 700 MHz Cryo Probe spectrometers at magnetic field strengths of 11.75 T (500 MHz) and 16.44 T (700 MHz) with operating frequencies 500.16 MHz and 700.13 MHz (for ^{1}H), 125.77 MHz and 176.04 MHz (for ^{13}C) at 23 °C, respectively. Chemical shifts (δ) are reported in parts per million (ppm) and coupling constants (J) are reported in Hertz (Hz). The ^{1}H and ^{13}C NMR chemical shifts (δ in ppm) were referenced to the residual signals of DMSO-d_6 [2.50 (^{1}H) and 39.52 (^{13}C)], $CDCl_3$ [7.26 (^{1}H) and 77.16 (^{13}C)], or MeOD [3.31 (^{1}H) and 49.00 (^{13}C)]. Structural assignment of resonances was performed with the help of 2D NMR gradient experiments (COSY, ^{1}H−^{13}C HSQC, ^{1}H−^{13}C HMBC, ^{1}H−^{15}N HSQC, ^{1}H−^{15}N HMBC, NOESY). Abbreviations in NMR spectra: app – apparent, br – broad, d – doublet, dd – doublet of doublets, h – heptet, m – multiplet, me – multiplicity edited, s – singlet, t – triplet.

HRMS measurements were recorded on an Agilent 6230 LC/TOF HRMS system using Electrospray ionisation (ESI) techniques. Mass values are reported within the 5 ppm error limit.

SDS-PAGE was carried using freshly made plates of 12% polyacrylamide running gel with a 4% polyacrylamide stacking gel. The analytes (20 μL, 0.25 μM final concentration in a reducing (with β -mercaptoethanol) or non-reducing Coomassie brilliant blue loading dye) were denatured by heating the sample to 90 °C for 5 min immediately before loading. Each gel was run with a 10-200 kDa molecular weight marker ladder (5 μL, New England BioLabs). Gels were run at 200 V for 65 min in Laemmli running buffer (LRB). All gels were stained with Coomassie dye and imaged on a Syngene gel imaging system.

Antibody-conjugate concentration in solution was determined by UV-vis spectroscopy using a NanoDrop One spectrophotometer. Sample buffer was used as the blank for baseline correction. Extinction coefficients from Expasy ProtParam. https://web.expasy.org/protparam/.

Protein LC-MS was performed on a Xevo G2-S TOF mass spectrometer coupled to an Acquity UPLC system using an Acquity UPLC BEH300 C4 column (1.7 μm, 2.1 × 50 mm). 0.1% Formic acid (aq.) (solvent A) and 95% MeCN with 5% 0.1% formic acid (aq.) (solvent B) were used as the mobile phase at a flow rate of 0.2 mL/min. The gradient was programmed as follows: 95% A for 0.93 min, then a gradient to 100% B over 4.28 min, then 100% B for 1.04 minutes, then a gradient to 95% A over 1.04 min. The electrospray source

was operated with a capillary voltage of 2.0 kV and a cone voltage of 190 V. Nitrogen was used as the desolvation gas at a total flow rate of 850 L/h. Total mass spectra were reconstructed from the ion series using the MaxEnt 1 algorithm preinstalled on MassLynx 4.2 software according to the manufacturer's instructions. Trastuzumab samples were deglycosylated with PNGase F (New England Biolabs) prior to LC-MS analysis. Only the region of each total ion chromatogram (TIC) containing protein signals was analysed. All calculated values for the masses of trastuzumab conjugates are based on the observed mass ion of native trastuzumab under the same preparation and ionisation conditions (145,171 Da).

Analytical size exclusion chromatography (SEC) was carried out using an Agilent 1260 Infinity system with a Sepax Zenix-C SEC-300column (30 cm × 7.8 mm, 3 µm) eluting with sodium phosphate buffer (54.7 mM NaH_2PO_4, 95.3 mM Na_2HPO_4, 100 mM NaCl, 0.02% (w/v) sodium azide, pH 7) over 30 minutes at a flow rate of 1 mL/min. HPLC was monitored by UV absorbance at 280 nm, and extent of aggregation was determined based on peak area.

Analytical hydrophobic interaction chromatography (HIC) was carried out using an Agilent 1260 Infinity system with a Tosoh TSKgel Butyl-NPR column (3.5 cm × 4.6 mm, 2.5 µm) eluting with a linear gradient of 100% solvent A for 3 min, then 0–100% solvent B in solvent A over 17 min or 0–70% solvent B in solvent A over 37 min (solvent A: 1.5 M ammonium sulfate, 25 mM Na_3PO_4, pH 7; solvent B: 25% (v/v) isopropyl alcohol in 25 mM Na_3PO_4, pH 7) at a flow rate of 0.6 mL/min. HPLC was monitored by UV absorbance at 280 nm, with conversion calculated by peak area.

1.3. Manuscript related structures

The key structures related to the main manuscript (please see below for experimental procedures and characterisation):

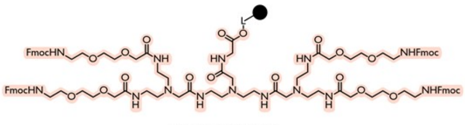

Tetra-PEG intermediate
S12

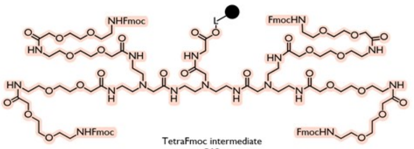

TetraFmoc intermediate
S13

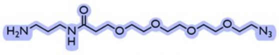

Azido spacer
S4

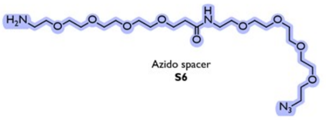

Azido spacer
S6

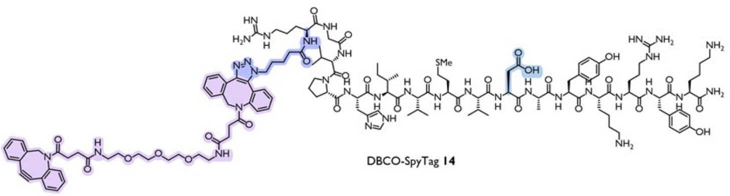

DBCO-SpyTag **14**

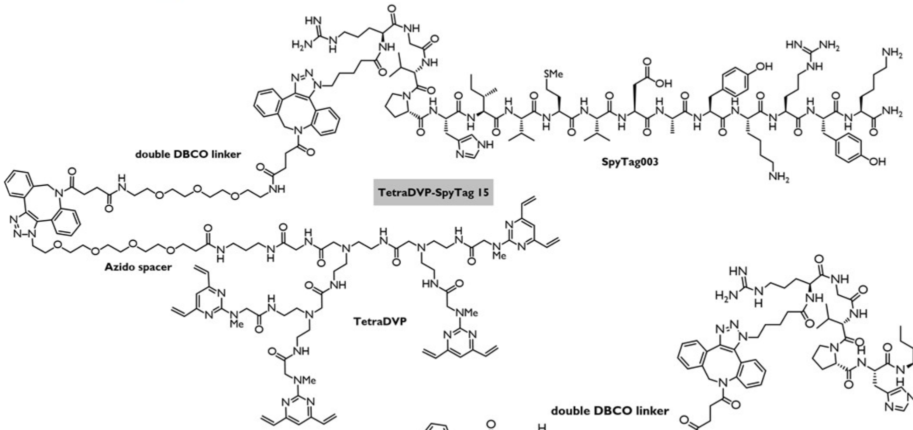

TetraDVP-SpyTag 15

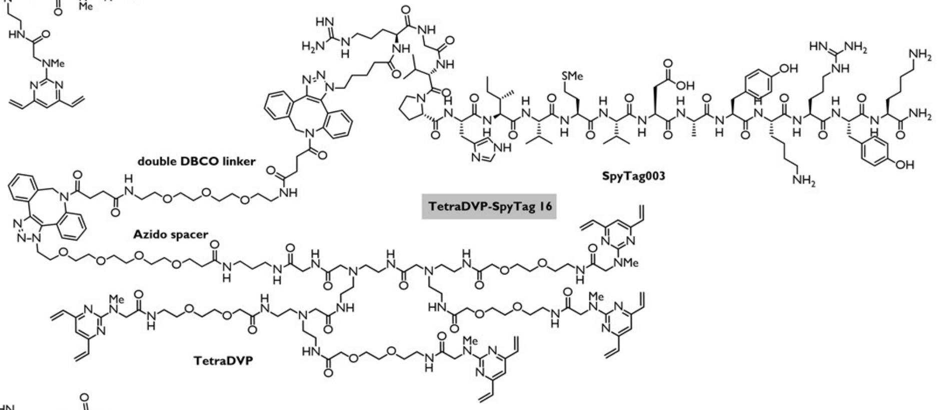

TetraDVP-SpyTag 16

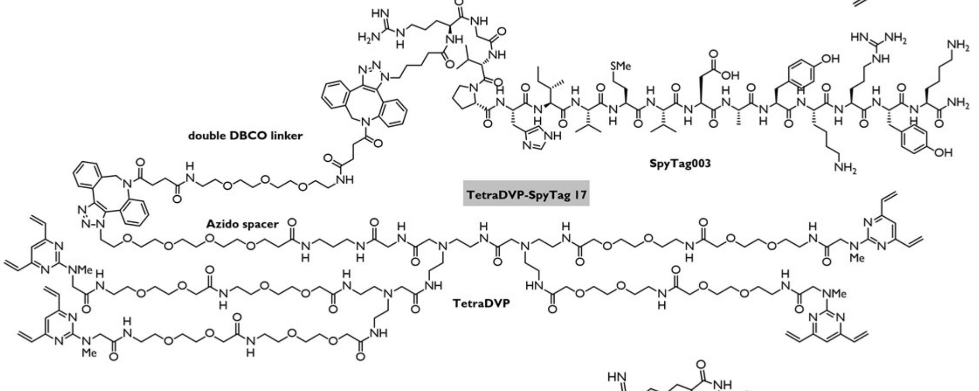

TetraDVP-SpyTag 17

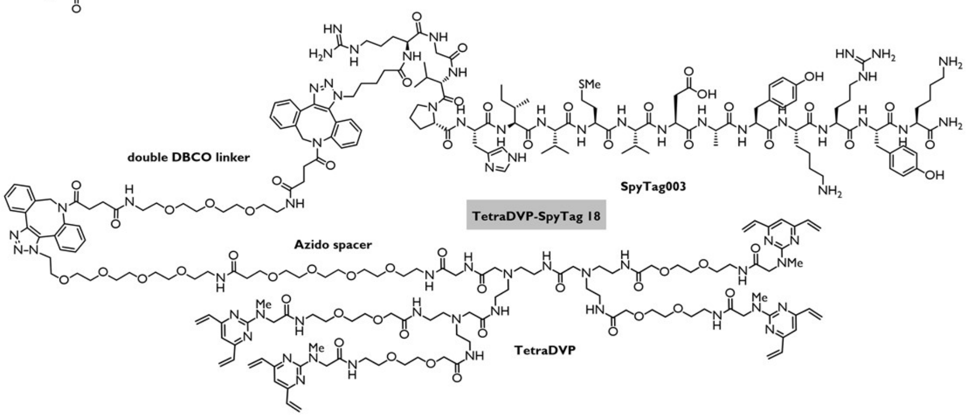

TetraDVP-SpyTag 18

1.4. Experimental Procedures

1.4.1. General procedure A: Amide coupling on resin

Corresponding Fmoc deprotected resin was washed with CH_2Cl_2 (3 × 10 mL). Corresponding carboxylic acid and HOBt were dissolved in DMF, followed by addition of DIC prior to the addition to the resin, unless stated otherwise. The reaction mixture was added to a plastic syringe with the resin at once and the slurry was shaken for 16 hours. The resin was washed with CH_2Cl_2 (3 × 10 mL), DMF (3 × 15 mL) and CH_2Cl_2 (5 × 10 mL) and small portions were cleaved and analysed by UV-LCMS.

*Note: in case of amide coupling with DVP, **S1** and HOBt were dissolved and added to the resin at once. DIC was then added directly to the resin, without pre-mixing in a beaker.*

1.4.2. General procedure B: Manual Fmoc deprotection

Corresponding resin was washed with CH_2Cl_2 (3 × 10 mL). Manual Fmoc deprotection was performed with 5% DBU in CH_2Cl_2 (350 µL in 7 mL of CH_2Cl_2 per 250 mg of resin) in a plastic syringe at ambient temperature for 20 minutes. The resin was washed with CH_2Cl_2 (3 ×10 mL), DMF (3 ×15 mL) and CH_2Cl_2 (5 ×10 mL) and used in the next step without further analysis.

1.4.3. General procedure C: Amide coupling in solution

Corresponding TetraDVP carboxylic acid was dissolved in DMSO (500 µL per 5 mg of acid), followed by addition of dissolved azido spacer in DMF and HOBt in DMF. Then, DIC was added and the reaction mixture was stirred for 16 hours. It was monitored by UV-LCMS which indicated full consumption of the starting material and formation of the product. The crude reaction mixture was directly purified by preparative HPLC with a neutral mobile phase (MeCN/water). Upon HPLC, the fractions were collected, combined, evaporated to dryness under a stream of nitrogen and used to the next step or stored in a freezer as a stock solution in DMSO.

1.4.4. General procedure D: SPAAC reaction

Corresponding TetraDVP azide was dissolved in DMSO in an Eppendorf tube. Corresponding DBCO-containing compound was dissolved in DMSO and added to the Eppendorf tube with TetraDVP azide. Water was added to the reaction mixture, and it was shaken for 5–24 hours upon which UV-LCMS analysis confirmed full consumption of the azide. The final compounds were purified on preparative HPLC.

1.4.5. Key branching amine 2

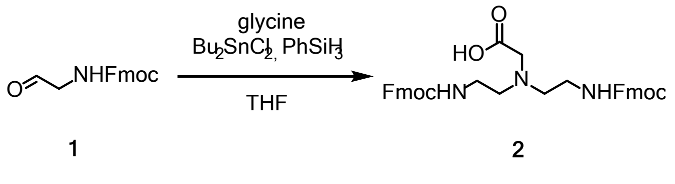

Commercially available (9H-fluoren-9-yl)methyl (2-oxoethyl)carbamate **1** (3 g, 10.65 mmol, 5 eq) was dissolved in anhydrous THF (20 mL), followed by addition of glycine (159 mg, 2.13 mmol, 1 eq) and Bu_2SnCl_2 (645 mg, 2.13 mmol, 1 eq). The mixture was vigorously stirred for 20 min at ambient temperature and then phenyl silane (1.31 mL, 10.65 mmol, 5 eq) was slowly added. The reaction mixture was heated up to reflux and stirred for 22 h after which UV-LCMS analysis confirmed full conversion to product **2**. The mixture was cooled down to ambient temperature, diluted with aq. NH_4Cl (500 mL) and extracted with EtOAc (3 × 600 mL). Organic extracts were combined, dried over $MgSO_4$, filtered and evaporated under reduced pressure. The crude product was purified by column chromatography (CH_2Cl_2/MeOH 12.5:1 to 1:2, v/v) to afford compound **2** as a white amorphous solid (1.27 g, 2.09 mmol, 98%).

LCMS R_t = 2.45 min (5–95% B over 5 min with constant 5% C over 1 min)

Analytical HPLC R_t = 11.81 min (5–95% B over 18 min)

^{1}**H NMR** (500 MHz, DMSO-d_6): δ 7.87 (d, *J* = 7.5 Hz, 4H), 7.66 (d, *J* = 7.5 Hz, 4H), 7.39 (t, *J* = 7.5 Hz, 4H), 7.30 (td, *J* = 7.5, 1.2 Hz, 4H), 7.22 (t, *J* = 5.7 Hz, 2H), 4.27 (d, *J* = 7.1 Hz, 4H), 4.19 (t, *J* = 7.0 Hz, 2H), 3.31 (s, 2H), 3.06 (q, *J* = 6.4 Hz, 4H), 2.67 (t, *J* = 6.7 Hz, 4H) ppm.

^{13}**C NMR** (126 MHz, DMSO-d_6): δ 173.1, 163.5, 156.6, 144.3, 141.1, 128.0, 127.5, 125.5, 120.5, 65.8, 53.8, 47.1 ppm.

HRMS calcd. for $C_{36}H_{36}N_3O_6$ *m/z* 606.2599 $[M+H]^+$, found 606.2586.

1.4.6. DVP S1

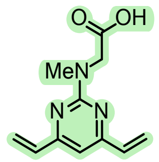

DVP **S1**

S1 was obtained according to the previously reported procedure.[1] The data agree with that previously reported.[1]
LCMS R_t = 2.06 min (5–95% B over 5 min with constant 5% C over 1 min)

Analytical HPLC R_t = 8.69 min (5–95% B over 18 min)

Purity: 99%

^{1}**H NMR** (500 MHz, MeOD): δ 6.68 – 6.59 (m, 3H), 6.40 (dd, *J* = 17.4, 1.7 Hz, 2H), 5.53 (dd, *J* = 10.6, 1.6 Hz, 2H), 4.27 (s, 2H), 3.27 (s, 3H) ppm.

^{13}**C NMR** (126 MHz, MeOD): δ 177.2, 163.3, 162.3, 136.2, 119.8, 103.5, 53.0, 35.1 ppm.

LRMS calcd. For $C_{11}H_{14}N_3O_2$ *m/z* $[M+H]^+$ 220.1, found 220.2.

1.4.7. Azido spacer S4

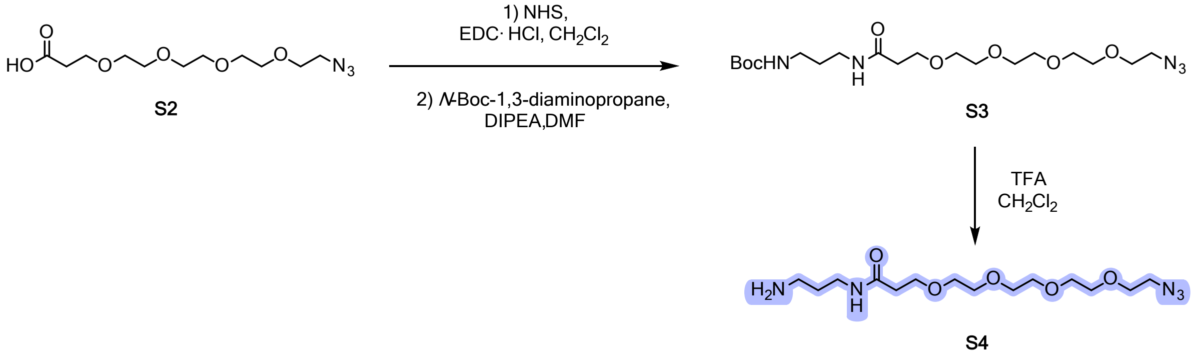

S3 was obtained as follows: Commercially available 15-azido-4,7,10,13-tetraoxapentadecanoic acid **S2** (1.5 g, 5.15 mmol, 1 eq.) and *N*-hydroxysuccinimide (890 mg, 7.73 mmol, 1.5 eq.) were dissolved in CH_2Cl_2 (30 mL). *N*-(3-Dimethylaminopropyl)-*N'*-ethylcarbodiimide hydrochloride (1.97 g, 10.3 mmol, 2 eq.) was added and the solution stirred for 16 h at rt. The solution was washed with sat. aq. $NaHSO_4$ (30 mL) then brine (30 mL). The organic layer was dried ($MgSO_4$), filtered, and concentrated *in vacuo*. The resulting crude orange oil was diluted with DMF (10 mL), *N*-boc-1,3-diaminopropane (896 mg, 5.15 mmol, 1 eq.) and DIPEA (1.8 mL, 10.30 mmol, 2 eq.) were added and the mixture stirred for 1 h at rt. The solution was diluted with EtOAc (50 mL) and extracted with sat. NH_4Cl (30 mL) then 5% aq. LiCl (4 x 30 mL). The organic layer was dried ($MgSO_4$), filtered and concentrated in vacuo to yield **S3** as an orange oil (1.86 g, 4.15 mmol, 81%).

^{1}H NMR (500 MHz, $CDCl_3$) δ 6.77 (s, 1H), 5.15 (s, 1H), 3.71 (t, J = 5.7 Hz, 2H), 3.68 – 3.59 (m, 14H), 3.37 (t, J = 5.1 Hz, 2H), 3.28 (app. q, J = 6.3 Hz, 2H), 3.13 (app. q, J = 6.1 Hz, 2H), 2.46 (t, J = 5.8 Hz, 2H), 1.61 (app. qn , J = 6.3 Hz, 2H), 1.42 (s, 9H) ppm.

^{13}C NMR (126 MHz, $CDCl_3$) δ 172.0, 156.4, 79.0, 70.71, 70.68, 70.6, 70.5, 70.4, 70.3, 70.0, 67.3, 50.7, 37.1, 37.0, 36.0, 30.1, 28.4 ppm.

The data agree with that previously reported.[1,2]

S4 was obtained as follows: **S3** (300 mg, 0.67 mmol) was dissolved in CH_2Cl_2 (5 mL) and trifluoracetic acid (1.25 mL) and stirred for 2 h at rt. The solvent was removed with a stream of nitrogen and the resulting oil purified by automated reverse phase flash column chromatography (10-50% MeCN in aq. 0.1% NH_4OH) to yield **S4** as a yellow oil (142 mg, 0.41 mmol, 61%).

^{1}H NMR (500 MHz, $CDCl_3$) δ 6.89 (app. s, 1H), 3.74 (t, J = 5.9 Hz, 2H), 3.71 – 3.61 (m, 14H), 3.40 (t, J = 5.0 Hz, 2H), 3.36 (app. q, J = 6.4 Hz, 2H), 2.78 (t, J = 6.5 Hz, 2H), 2.48 (t, J = 5.8 Hz, 2H), 1.65 (app. qn, J = 6.6 Hz, 4H) ppm.

^{13}C NMR (126 MHz, $CDCl_3$) δ 171.6, 70.70, 70.68, 70.6, 70.5, 70.4, 70.3, 70.1, 67.4, 50.7, 39.8, 37.3, 37.1, 32.8 ppm.

HRMS calcd. for $C_{14}H_{30}N_5O_5$ *m/z* $[M+H]^+$ 348.2241, found 348.2245.

The data agree with that previously reported.[1,2]

1.4.8. Azido spacer S6

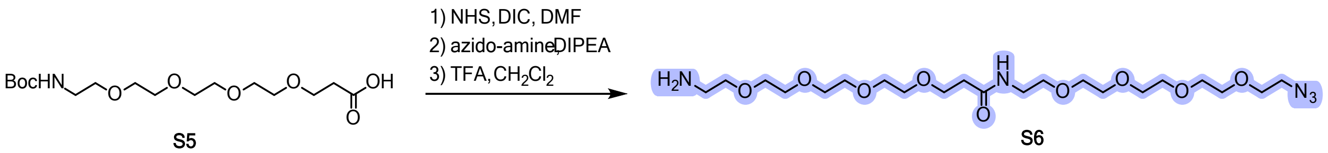

S5

S6

Commercially available 2,2-dimethyl-4-oxo-3,8,11,14,17-pentaoxa-5-azaicosan-20-oic acid **S5** (500 mg, 1.37 mmol, 1 eq.) and *N*-hydroxysuccinimide (473 mg, 4.11 mmol, 3 eq.) were dissolved in DMF (3 mL). *N*,*N*'-Diisopropylcarbodiimide (319 µL, 2.05 mmol, 1.5 eq.) was added dropwise over 2 min and the solution stirred for 16 h at rt. 14-Azido-3,6,9,12-tetraoxatetradecan-1-amine (358 mg, 1.37 mmol, 1 eq.) and DIPEA (238 µL, 1.37 mmol, 1 eq.) were added and the resulting mixture stirred for a further 1 h at rt. The solution was washed with sat. aq. $NaHSO_4$ (30 mL) then brine (2 x 30 mL). The organic layer was dried ($MgSO_4$), filtered, and concentrated *in vacuo* to afford an orange oil. The crude oil was diluted with a CH_2Cl_2/TFA 1:1 solution (5 mL) and stirred at rt for 4 h. The solvent was removed with a stream of nitrogen and the resulting oil purified by automated reverse phase flash column chromatography (10-70% MeCN in aq. 0.1% NH_4OH) to yield **S6** as a yellow oil (82 mg, 0.16 mmol, 12%).

^{1}H NMR (500 MHz, MeOD) δ 3.75 (t, J = 6.2 Hz, 2H), 3.72 – 3.60 (m, 26H), 3.56 (t, J = 5.5 Hz, 2H), 3.53 (t, J = 5.3 Hz, 2H), 3.43 – 3.36 (m, 4H), 2.80 (t, J = 5.0 Hz, 2H), 2.48 (t, J = 6.2 Hz, 2H) ppm.

^{13}C NMR (126 MHz, MeOD) δ 172.6, 72.2, 70.26, 70.25, 70.22, 70.21, 70.20, 70.19, 70.1, 69.99, 69.95, 69.9, 69.8, 69.2, 66.9, 50.4, 40.8, 39.1, 36.2 ppm.

HRMS calcd. for $C_{21}H_{44}N_5O_9$ *m/z* $[M+H]^+$ 510.3134, found 510.3147.

1.4.9. Double DBCO linker 12

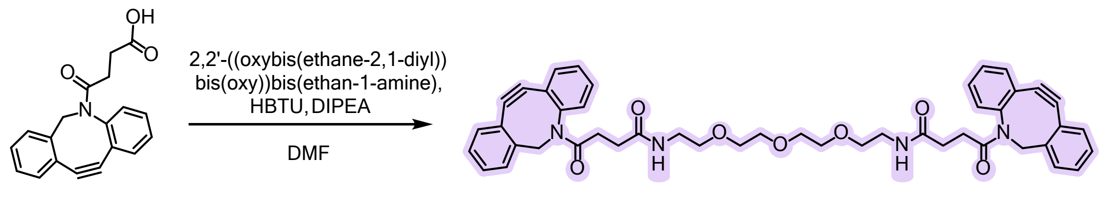

DBCO acid

12; doubleDBCO linker

To a solution of 2,2'-((oxybis(ethane-2,1-diyl))bis(oxy))bis(ethan-1-amine) (142 mg, 0.74 mmol, 1 eq.), DBCO-acid* (500 mg, 1.64 mmol, 2.2 eq.), and HBTU (644 mg, 1.70 mmol, 2.3 eq.) in DMF (10 mL) was added DIPEA (386 µL, 2.22 mmol, 3 eq.) and the resulting mixture stirred for 1 h at rt. The solution was diluted with EtOAc (30 mL) and washed with water (30 mL) followed by 5% aq. LiCl (3 x 30 mL). The organic layer was dried ($MgSO_4$), filtered, and concentrated *in vacuo*. The crude oil was purified *via* flash column chromatography (0-4% MeOH in CH_2Cl_2) to afford **12** as a light-yellow oil (381 mg, 0.50 mmol, 67%).

LCMS R_t = 2.79 min (5–95% B over 5 min with constant 5% C over 1 min)

Analytical HPLC R_t = 12.61 min (5–95% B over 18 min)

^{1}H NMR (700 MHz, $CDCl_3$) δ 7.71 – 7.62 (m, 2H), 7.56 – 7.50 (m, 2H), 7.45 – 7.36 (m, 6H), 7.36 – 7.30 (m, 2H), 7.30 – 7.26 (m, 2H), 7.27 – 7.22 (m, 2H), 6.36 – 6.26 (m, 2H), 5.16 (d, *J* = 14.0 Hz, 2H), 3.67 (d, *J* = 14.1 Hz, 2H), 3.63 – 3.51 (m, 8H), 3.51 – 3.39 (m, 4H), 3.37 – 3.25 (m, 4H), 2.87 – 2.79 (m, 2H), 2.50 – 2.41 (m, 2H), 2.21 – 2.09 (m, 2H), 2.00 – 1.89 (m, 2H) ppm.

^{13}C NMR (176 MHz, CDCl3) δ 172.3, 172.1, 151.4, 148.1, 132.3, 129.4, 128.7, 128.2, 128.1, 127.7, 127.0, 125.5, 123.2, 122.5, 114.6§, 107.9§, 70.4, 70.1, 69.7, 55.5, 39.1, 31.1, 30.2 ppm.

HRMS calcd. For $C_{46}H_{47}N_4O_7$ *m/z* $[M+H]^+$ 767.3445, found 767.3476.

**DBCO-acid was kindly donated by Dr Tim Schober and synthesised according to literature.*[3]
§*Presence determined by HSQC and HMBC*

1.4.10. SpyTag-azide 13

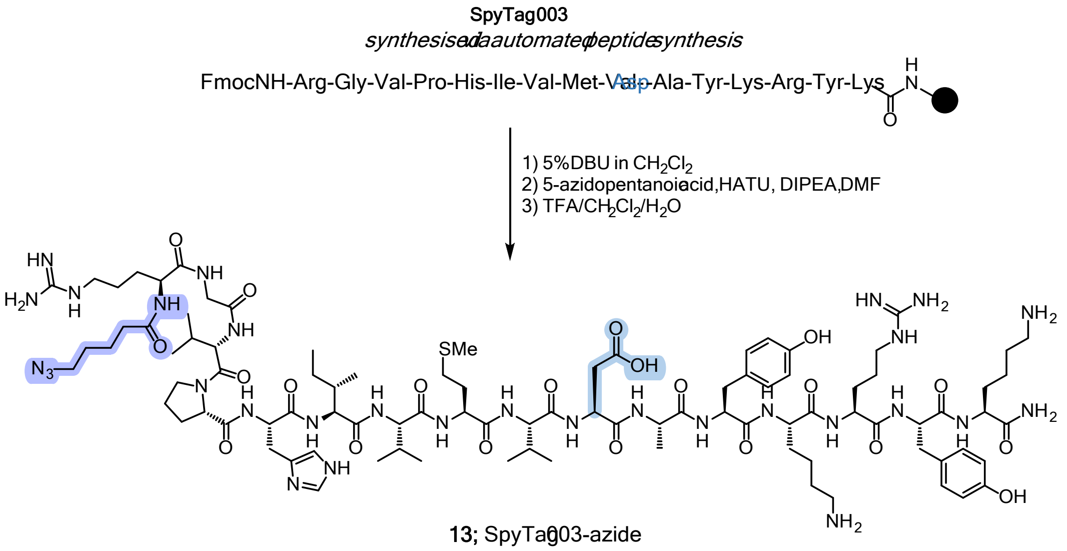

13; SpyTag003-azide

13 was obtained by General procedures B and A, followed by cleavage from the resin and HPLC purification. From Rink amide resin 0.125 mmol (400 mg). 5-azidopentanoic acid (150 µL, 1.2 mmol, 0.3M), HATU (456 mg, 1.2 mmol, 0.3M), DIPEA (400 µL, 2.4 mmol, 0.6M), DMF (4 mL). White amorphous solid. Overall yield: 120 mg, 47%.

LCMS R_t = 1.32 min (5–95% B over 5 min with constant 5% C over 1 min)

Analytical HPLC R_t = 6.72 min (5–95% B over 18 min)

Purity: 96%

HRMS calcd. for $C_{93}H_{151}N_{30}O_{21}S$ *m/z* $[M+H]^{2+}$ 2057.1458, found $[M+H]^{2+}$ 2057.1429.

1.4.11. DBCO-SpyTag 14

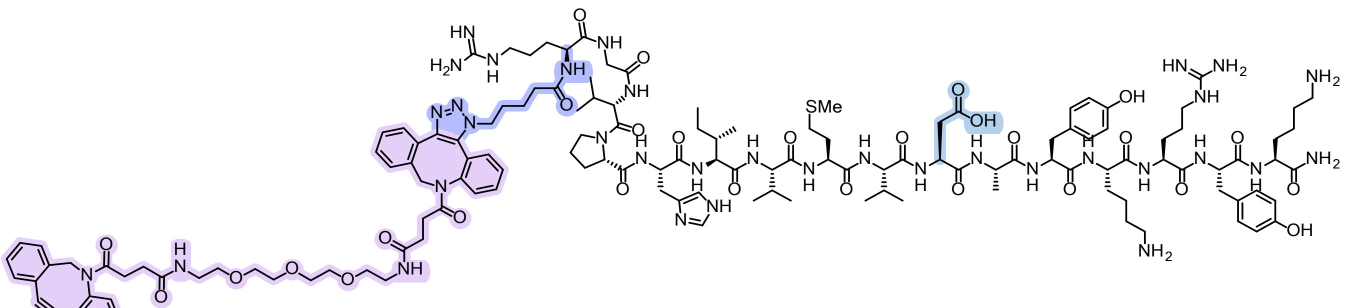

14; DBCO-SpyTag

14 was obtained by General procedure D, followed by HPLC purification. From **13** (27 mg, 0.013 mmol) and **12** (27 mg, 0.032 mmol). White amorphous solid. Yield: 35 mg, 94%. *Caution: it is recommended that the compound be stored as a solid after purification to prevent partial DBCO scaffold hydrolysis over time. This is only an issue before the SPAAC occurs.*

LCMS R_t = 1.63 min (5–95% B over 5 min with constant 5% C over 1 min)

Analytical HPLC R_t = 8.03 min (5–95% B over 18 min)

Purity: 97%

LRMS calcd. for $C_{139}H_{197}N_{34}O_{28}S$ *m/z* $[M+H]^+$ 2822.47, found $[M/4+H]^+$ 706.94.

2. Synthesis of TetraDVP linkers

2.1. Synthesis of TetraFmoc compound 4

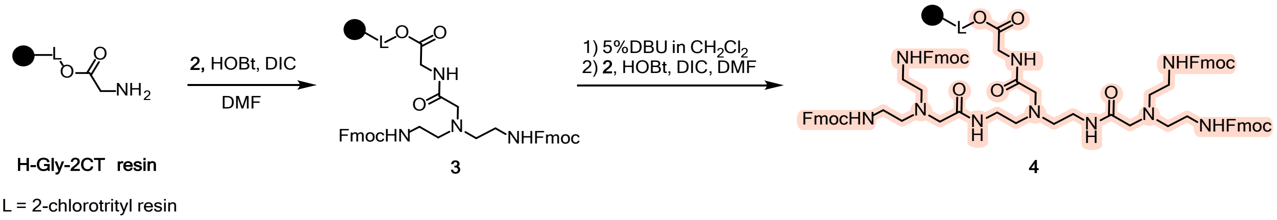

3 was obtained by General procedure A and B. **2** (317 mg, 0.525 mmol), HOBt (80 mg, 0.525 mmol), DIC (82 µL, 0.525 mmol), DMF (3.5 mL). An analytical sample was subjected to a mini-cleavage in CH_2Cl_2/HFIP cleavage cocktail and analysed by UV-LCMS.

LCMS R_t = 2.48 min (5–95% B over 5 min with constant 5% C over 1 min)

Analytical HPLC R_t = 11.19 min (5–95% B over 18 min)

Crude purity: 98%

LRMS calcd. for $C_{38}H_{39}N_4O_7$ *m/z* [M+H]+ 663.28, found 663.30.

4 was obtained by General procedure A and B. **2** (317 mg, 0.525 mmol), HOBt (80 mg, 0.525 mmol), DIC (82 µL, 0.525 mmol), DMF (3.5 mL). An analytical sample was subjected to a mini-cleavage in CH_2Cl_2/HFIP cleavage cocktail and analysed by UV-LCMS.

LCMS R_t = 3.14 min (5–95% B over 5 min with constant 5% C over 1 min)

Analytical HPLC R_t = 12.13 min (5–95% B over 18 min)

Crude purity: 96%

LRMS calcd. for $C_{80}H_{85}N_{10}O_{13}$ *m/z* [M+H]+ 1393.63, found 1393.52.

2.2. Synthesis of Tetra-Fmoc-PEG compounds S12 and S13

- **Normal TetraFmoc-PEG S12**

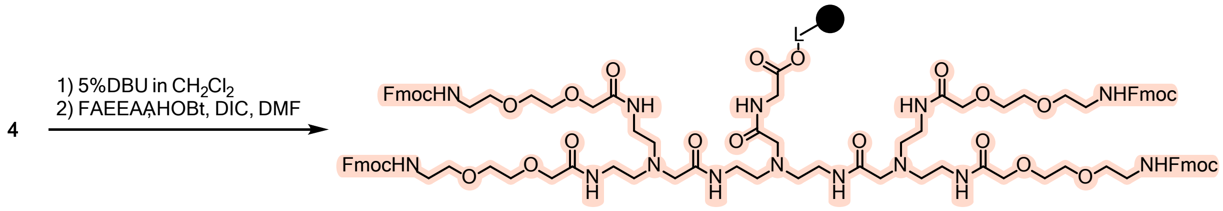

S12

S12 was obtained by General procedure A and B. Resin **4** (250 mg), FAEEAA (346 mg, 0.9 mmol), HOBt (137 mg, 0.9 mmol), DIC (142 µL, 0.9 mmol), DMF (6 mL). An analytical sample was subjected to a mini-cleavage in CH_2Cl_2/HFIP cleavage cocktail and analysed by UV-LCMS.

LCMS R_t = 2.91 min (5–95% B over 5 min with constant 5% C over 1 min)

Analytical HPLC R_t = 11.60 min (5–95% B over 18 min)

Crude purity: 95%

LRMS calcd. for $C_{104}H_{129}N_{14}O_{25}$ *m/z* $[M+H]^+$ 1973.92, found $[M/2+H]^+$ 988.22.

- **Long TetraFmoc-PEG S13**

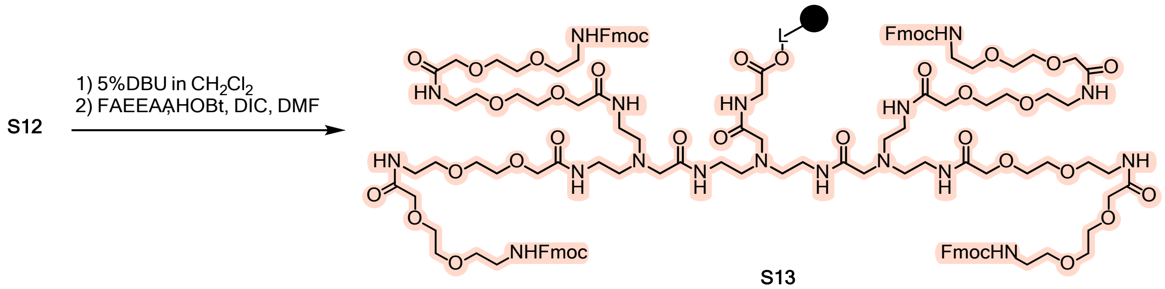

S13

S13 was obtained by General procedure A and B. Resin **S12** (250 mg), FAEEAA (346 mg, 0.9 mmol), HOBt (137 mg, 0.9 mmol), DIC (142µL, 0.9 mmol), DMF (6 mL). An analytical sample was subjected to a mini-cleavage in CH_2Cl_2/HFIP cleavage cocktail and analysed by UV-LCMS.

LCMS R_t = 2.59 min (5–95% B over 5 min with constant 5% C over 1 min)

Analytical HPLC R_t = 11.15 min (5–95% B over 18 min)

Crude purity: 95%

LRMS calcd. for $C_{128}H_{173}N_{18}O_{37}$ *m/z* [M+H]+ 2554.22, found 2554.62.

2.3. Synthesis of TetraDVP acids

- **Short TetraDVP-acid 5**

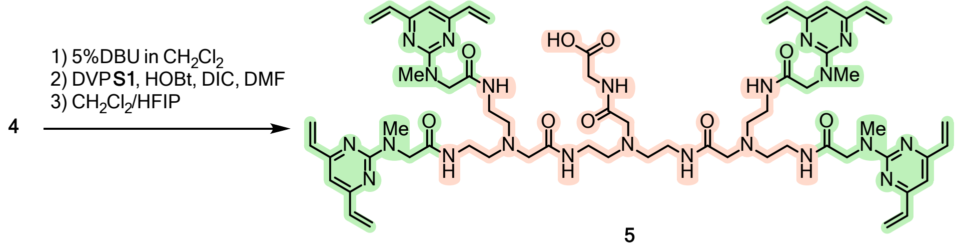

5

5 was obtained by General procedure A and B. Resin **4** (250 mg), DVP **S1** (197 mg, 0.9 mmol), HOBt (137 mg, 0.9 mmol), DIC (142 µL, 0.9 mmol), DMF (6 mL). An analytical sample was subjected to a mini-cleavage in CH_2Cl_2/HFIP cleavage cocktail and analysed by UV-LCMS. Compound was then subjected to a full cleavage from the resin in CH_2Cl_2/HFIP cleavage cocktail. Yellowish oil. Overall yield: 30 mg, 21%.

LCMS R_t = 9.45 min (5–95% B over 15 min with 1 min isocratic hold before the gradient)

Analytical HPLC R_t = 9.55 min (5–95% B over 18 min)

Purity after HPLC: 98%

LRMS calcd. for $C_{64}H_{90}N_{22}O_9$ *m/z* $[M+H]^{2+}$ 1310.73, found 1310.27.

- **Normal TetraDVP-acid 6**

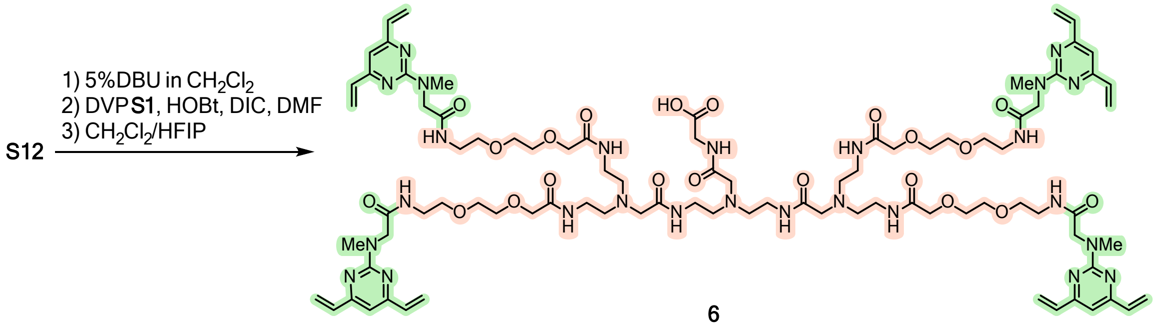

6

6 was obtained by General procedure A and B. Resin **S16** (250 mg), DVP **S1** (197 mg, 0.9 mmol), HOBt (137 mg, 0.9 mmol), DIC (142 µL, 0.9 mmol), DMF (6 mL). Upscale reaction (1 g) has been carried out in four separate plastic 12 mL syringes. An analytical sample was subjected to a mini-cleavage in CH_2Cl_2/HFIP cleavage cocktail and analysed by UV-LCMS. Compound **6** was then subjected to a full cleavage from the resin in CH_2Cl_2/HFIP cleavage cocktail and purified on HPLC. Yellowish oil. Overall yield: 150 mg, 48%.

LCMS R_t = 8.67 min (5–95% B over 15 min with 1 min isocratic hold before the gradient)

Analytical HPLC R_t = 8.91 min (5–95% B over 18 min)

Purity after HPLC: 99%

LRMS calcd. for $C_{88}H_{134}N_{26}O_{21}$ *m/z* $[M+H]^{2+}$ 1891.02, found 1891.39.

- **Long TetraDVP-acid 7**

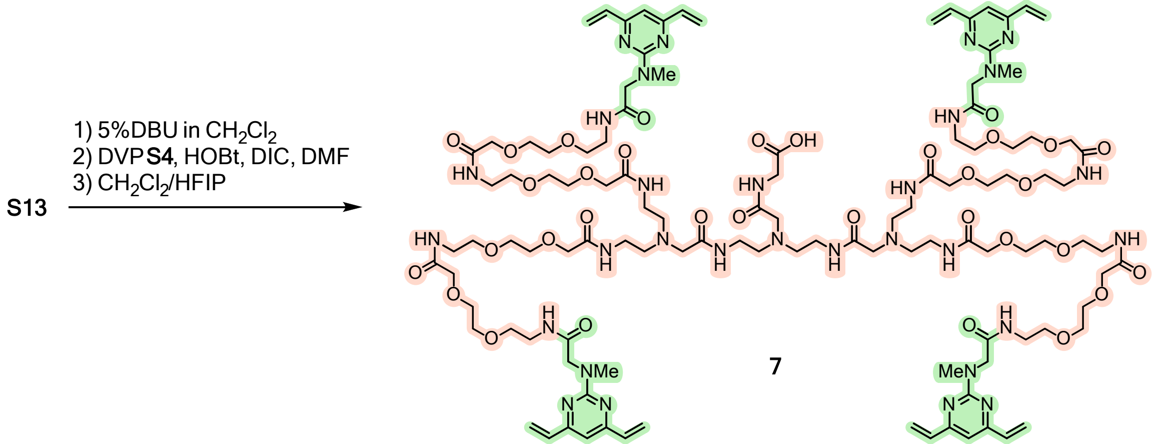

7 was obtained by General procedure A and B. Resin **S13** (250 mg), DVP **S1** (197 mg, 0.9 mmol), HOBt (137 mg, 0.9 mmol), DIC (142 µL, 0.9 mmol), DMF (6 mL). An analytical sample was subjected to a mini-cleavage in CH_2Cl_2/HFIP cleavage cocktail and analysed by UV-LCMS. Compound **7** was then subjected to a full cleavage from the resin in CH_2Cl_2/HFIP cleavage cocktail. Yellowish solid. Crude overall yield of the upscale reaction from 1g of H-Gly-O-2-CT resin (after 9 steps): 1.8 g, 83% yield.

LCMS R_t = 8.20 min (5–95% B over 15 min with 1 min isocratic hold before the gradient)

Analytical HPLC R_t = 8.40 min (5–95% B over 18 min)

Crude purity: 95% (HPLC)

^{1}H NMR (700 MHz, DMSO): δ 7.96 – 7.92 (m, 7H), 7.80 – 7.74 (app br s, 4H), 7.68 (t, *J* = 6.0 Hz, 4H), 6.79 (s, 4H), 6.63 – 6.59 (m, 8H), 6.40 – 6.38 (m, 8H), 5.60 – 5.58 (m, 8H), 4.19 (s, 8H), 3.86 – 3.85 (m, 18H), 3.55 – 3.50 (m, 40H), 3.43 – 3.41 (m, 8H) *overlap with solvent*, 3.26 (app q, *J* = 6.0 Hz, 16H), 3.22 (app q, *J* = 5.9 Hz, 12H), 3.16 (app br s, 18H), 2.60 – 2.54 (m, 12H) ppm.

^{13}C NMR (176 MHz, DMSO) δ 170.8, 169.5, 169.4, 169.3, 162.4, 161.8, 136.0, 121.8, 104.8, 70.2, 70.2, 70.0, 69.9, 69.4, 69.3, 69.1, 68.9, 57.8, 54.9, 53.9, 52.2, 38.5, 38.0, 36.5 ppm.

HRMS calcd. for $C_{112}H_{177}N_{30}O_{33}$ *m/z* 2470.3089 $[M+H]^+$, found $[M+H]^+$ 2470.3101.

2.4. Synthesis of TetraDVP azides

- **Short TetraDVP-azide 8**

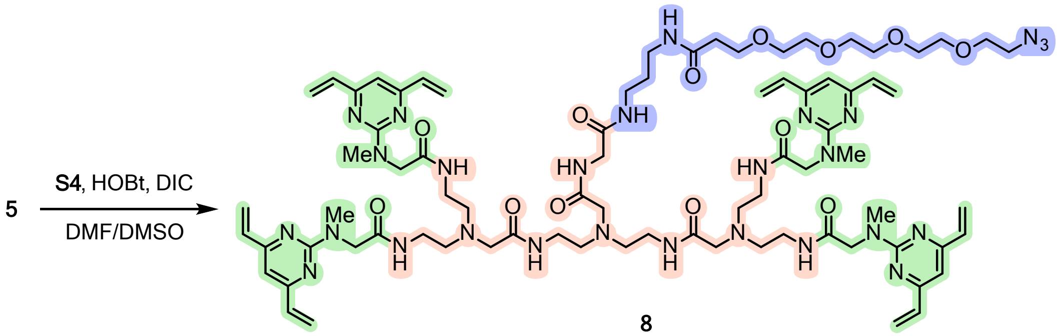

8

8 was obtained by General procedure C. **5** (5 mg, 0.003 mmol, 1 eq), **S4** (2 mg, 0.005 mmol, 1.5 eq), HOBt (10 mg, 0.065 mmol, 17 eq), DIC (11 µL, 0.065 mmol, 17 eq), DMF (800 µL), DMSO (500 µL). Upon completion, crude product has been purified on preparative HPLC with neutral mobile phase (MeCN/water without additives). Yellowish oil. Yield: 2.33 mg, 47%.

LCMS R_t = 10.42 min (45–75% B over 15 min with 1 min isocratic hold before the gradient)

Analytical HPLC R_t = 9.83 min (5–95% B over 18 min)

Purity: 99%

LRMS calcd. for $C_{78}H_{118}N_{27}O_{13}$ *m/z* $[M+H]^{3+}$ 1640.93, found 1640.44.

- **Normal TetraDVP-azide 9**

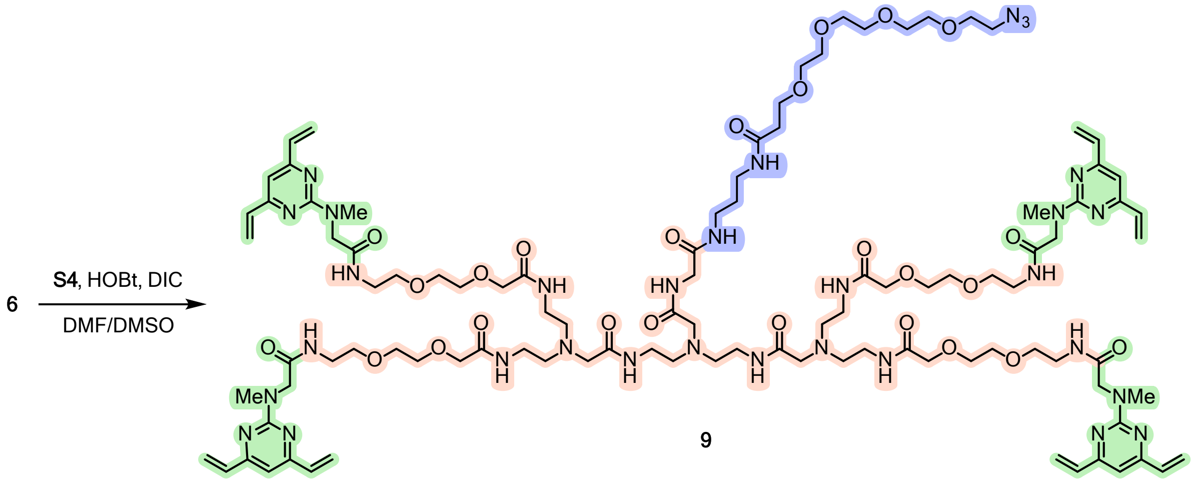

9

9 was obtained by General procedure C. **6** (10 mg, 0.005 mmol, 1 eq), **S4** (2.75 mg, 0.008 mmol, 1.5 eq), HOBt (13 mg, 0.085 mmol, 17 eq), DIC (13 µL, 0.085 mmol, 17 eq), DMF (800 µL), DMSO (1 mL). Upon completion, crude product has been purified on preparative HPLC with neutral mobile phase (MeCN/water without additives). Yellowish oil. Yield: 10 mg, 85%.

LCMS R_t = 8.80 min (5–95% B over 15 min with 1 min isocratic hold before the gradient)

Analytical HPLC R_t = 9.20 min (5–95% B over 18 min)

Purity: 98%

LRMS calcd. for $C_{102}H_{161}N_{31}O_{25}$ *m/z* $[M+H]^{2+}$ 2220.22, found $[M/2+H]^{2+}$ 1110.94.

- **Long TetraDVP-azide 10**

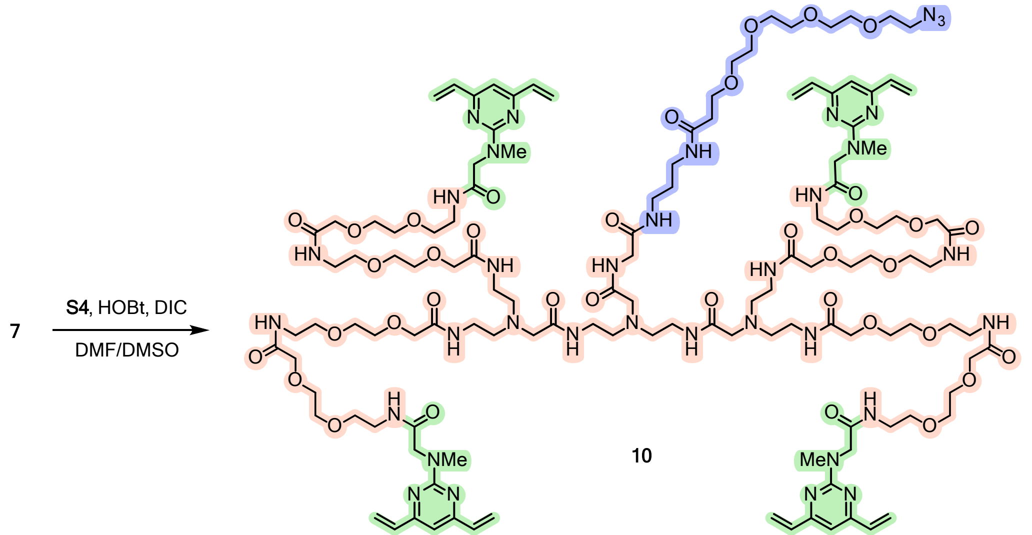

10 was obtained by General procedure C. **7** (15 mg, 0.006 mmol, 1 eq), **S4** (3 mg, 0.009 mmol, 1.5 eq), HOBt (16 mg, 0.102 mmol, 17 eq), DIC (14 µL, 0.102 mmol, 17 eq), DMF (200 µL), DMSO (1 mL), water (200 µL). Upon completion, crude product has been purified on preparative HPLC with neutral mobile phase (MeCN/water without additives). Yellowish oil. Yield: 8 mg, 47%.

LCMS R_t = 8.55 min (5–95% B over 15 min with 1 min isocratic hold before the gradient)

Analytical HPLC R_t = 8.79 min (5–95% B over 18 min)

Purity: 98%

LRMS calcd. for $C_{126}H_{205}N_{35}O_{37}$ *m/z* $[M+H]^{2+}$ 2800.52, found $[M/4+H]^{2+}$ 701.10.

- **Extended TetraDVP-azide 11**

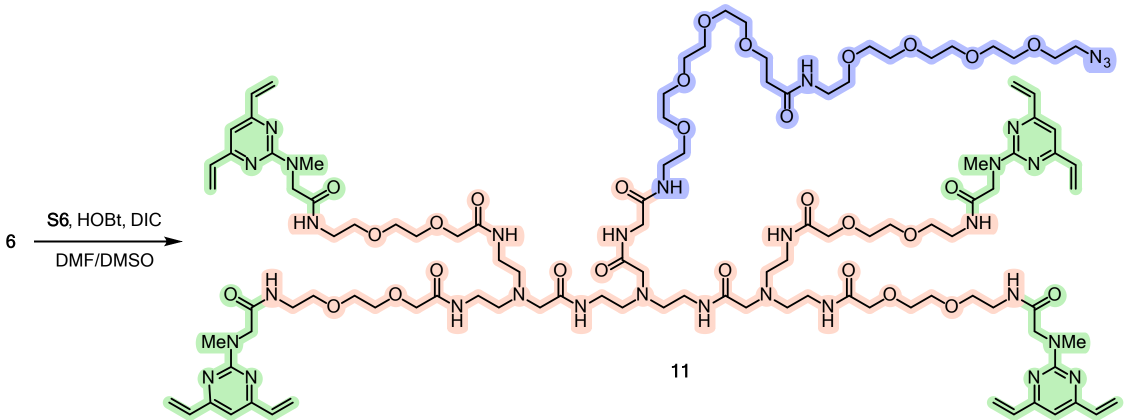

11 was obtained by General procedure C. **6** (10 mg, 0.005 mmol, 1 eq), **S6** (4 mg, 0.008 mmol, 1.5 eq), HOBt (13 mg, 0.085 mmol, 17 eq), DIC (13 µL, 0.085 mmol, 17 eq), DMF (800 µL), DMSO (1 mL). Upon completion, crude product has been purified on preparative HPLC with neutral mobile phase (MeCN/water without additives). Yellowish oil. Yield: 5 mg, 42%.

LCMS R_t = 12.48 min (5–95% B over 15 min) *neutral*

Analytical HPLC R_t = 9.17 min (5–95% B over 18 min) *acidic*

Purity: 95%

LRMS calcd. for $C_{109}H_{175}N_{31}O_{29}$ m/z $[M+H]^{2+}$ 2382.31, found $[M/2]^{2+}$ 1191.95.

2.5. Synthesis of final TetraDVP-SpyTag conjugates

- **Final TetraDVP-SpyTag conjugate 15**

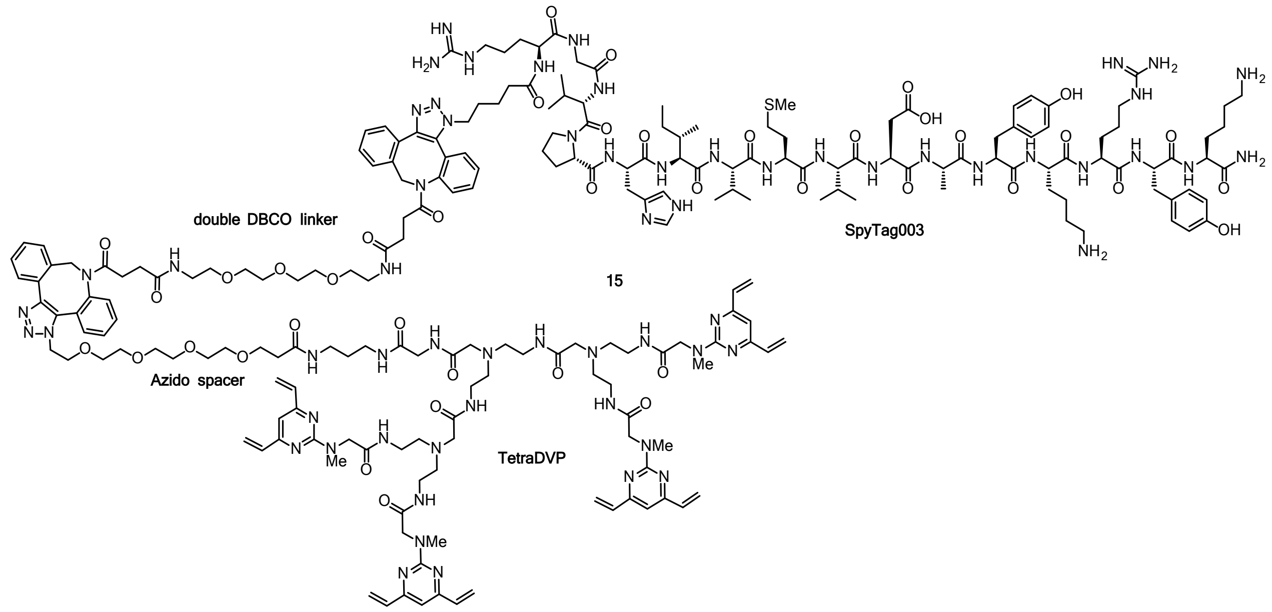

15 was obtained by General procedure D, followed by HPLC purification. **14** (1.5 mg in 250 µL DMSO), **8** (0.77 mg in 500 µL DMSO), water (500 µL). White amorphous solid. Yield: 0.66 mg, 31%.

LCMS R_t = 7.07 min (5–95% B over 15 min with 1 min isocratic hold before the gradient)

Analytical HPLC R_t = 8.73 min (5–95% B over 18 min)

Purity: 95%

LRMS calcd. for $C_{217}H_{312}N_{61}O_{41}S$ *m/z* $[M+H]^+$ 4460.39, found $[M/7]^+$ 638.54.

- **Final TetraDVP-SpyTag conjugate 16**

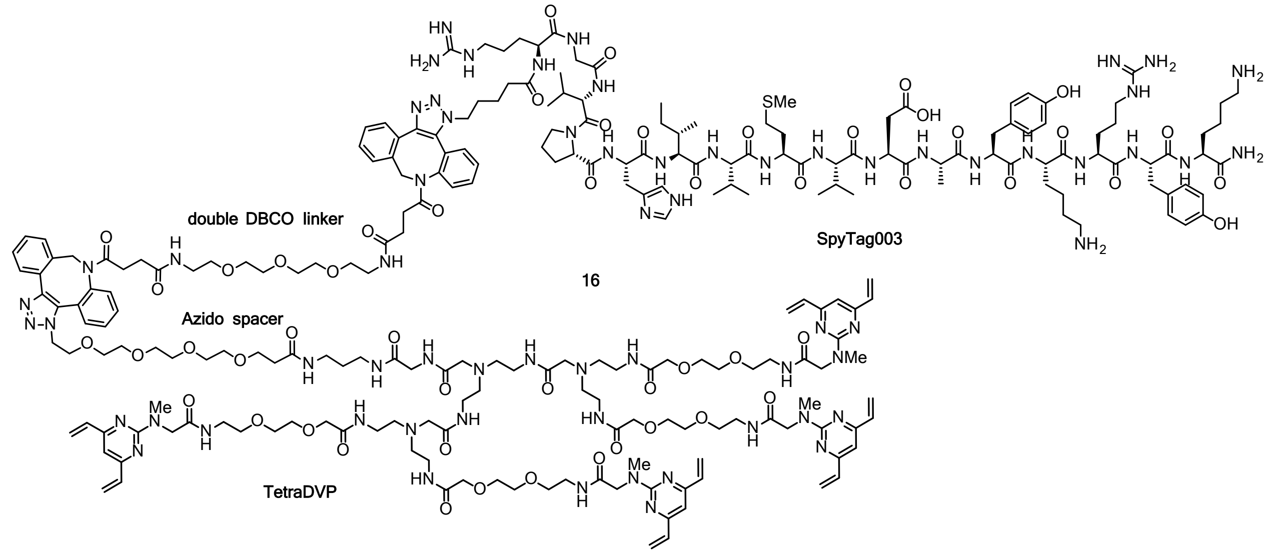

16 was obtained by General procedure D, followed by HPLC purification. **14** (2.5 mg in 250 µL DMSO), **9** (1.7 mg in 250 µL DMSO), water (500 µL). White amorphous solid. Yield: 0.34 mg, 10%.

LCMS R_t = 7.04 min (5–95% B over 15 min with 1 min isocratic hold before the gradient)

Analytical HPLC R_t = 8.37 min (5–95% B over 18 min)

Purity: 98%

LRMS calcd. for $C_{241}H_{356}N_{65}O_{53}S$ *m/z* $[M+H]^+$ 5040.68, found $[M/7]^+$ 721.51, $[M/8]^+$ 631.44.

- **Final TetraDVP-SpyTag conjugate 17**

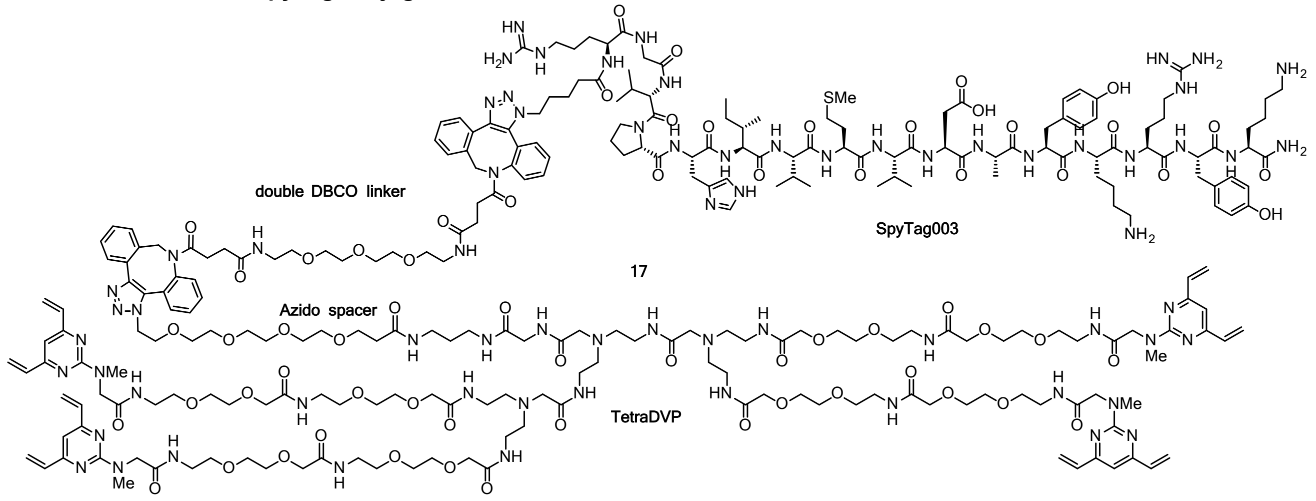

17

17 was obtained by General procedure D, followed by HPLC purification. **14** (2.5 mg in 250 µL DMSO), **10** (1 mg in 500 µL DMSO), water (500 µL). White amorphous solid. Yield: 0.17 mg, 10%.

LCMS R_t = 6.88 min (5–95% B over 15 min with 1 min isocratic hold before the gradient)

Analytical HPLC R_t = 8.19 min (5–95% B over 18 min)

Purity: 95%

LRMS calcd. for $C_{265}H_{400}N_{69}O_{65}S$ *m/z* $[M+H]^+$ 5620.98, found $[M/9]^+$ 625.92.

- **Final TetraDVP-SpyTag conjugate 18**

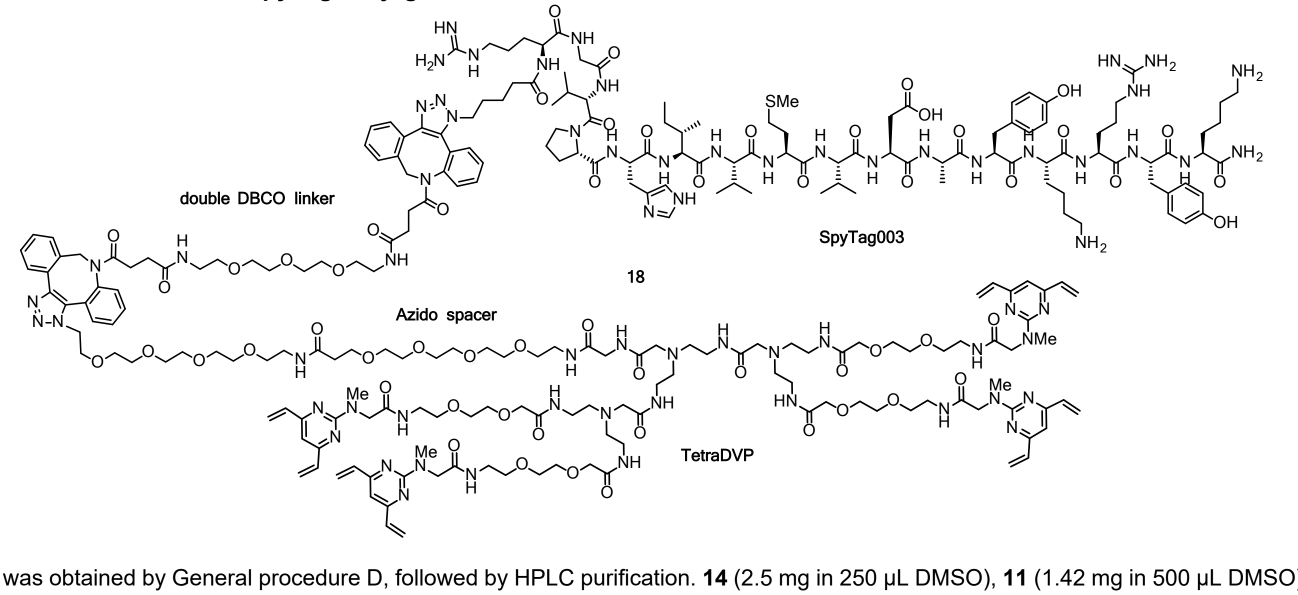

18

18 was obtained by General procedure D, followed by HPLC purification. **14** (2.5 mg in 250 µL DMSO), **11** (1.42 mg in 500 µL DMSO), water (500 µL). White amorphous solid. Yield: 0.57 mg, 18%.

LCMS R_t = 6.93 min (5–95% B over 15 min with 1 min isocratic hold before the gradient)

Analytical HPLC R_t = 8.40 min (5–95% B over 18 min)

Purity: 97%

LRMS calcd. for $C_{248}H_{370}N_{65}O_{57}S$ m/z $[M+H]^+$ 5202.77, found $[M/9]^+$ 579.43.

3. Analytical HPLC spectra

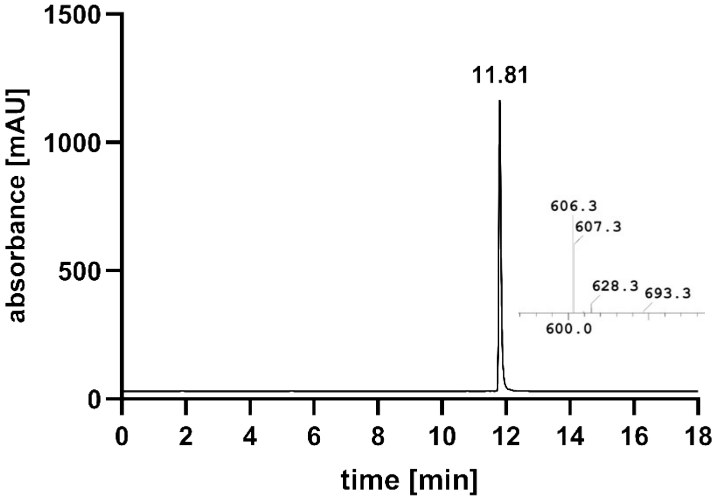

Analytical HPLC chromatogram of the key branching amine 2

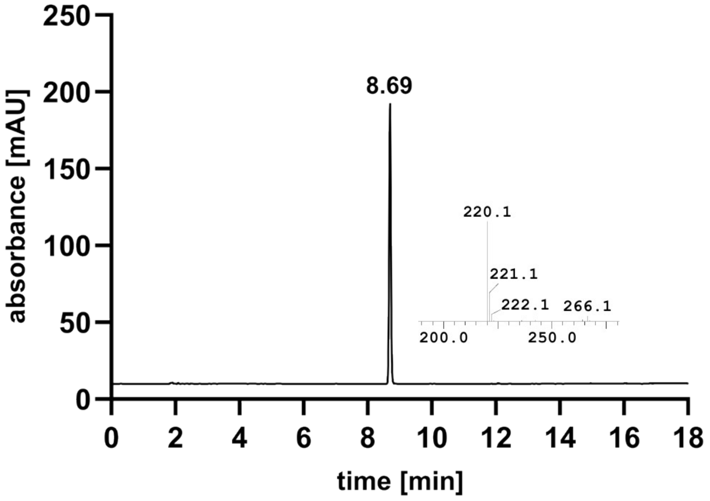

Analytical HPLC chromatogram of DVP S1

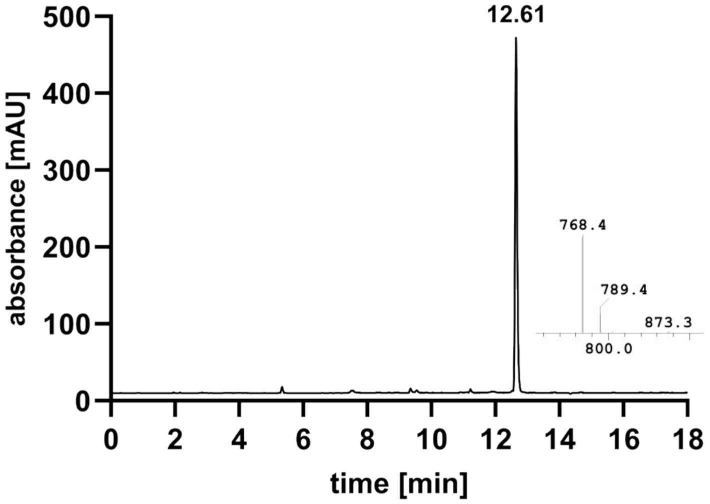

Analytical HPLC chromatogram of the double DBCO linker 12

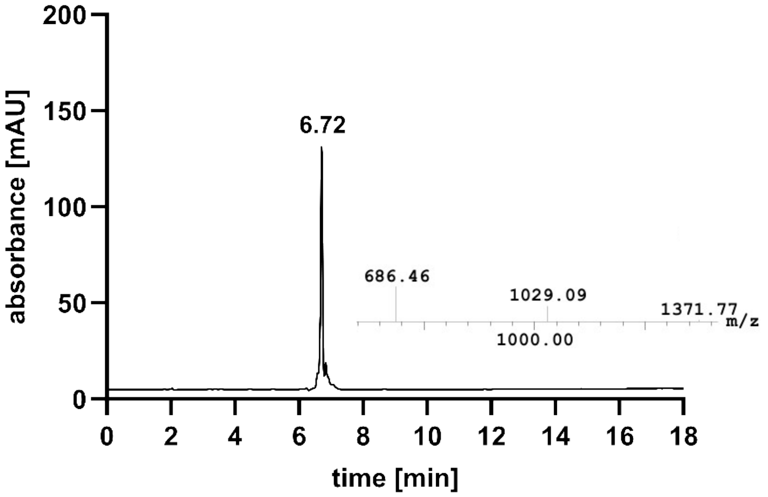

Analytical HPLC chromatogram of SpyTag azide 13

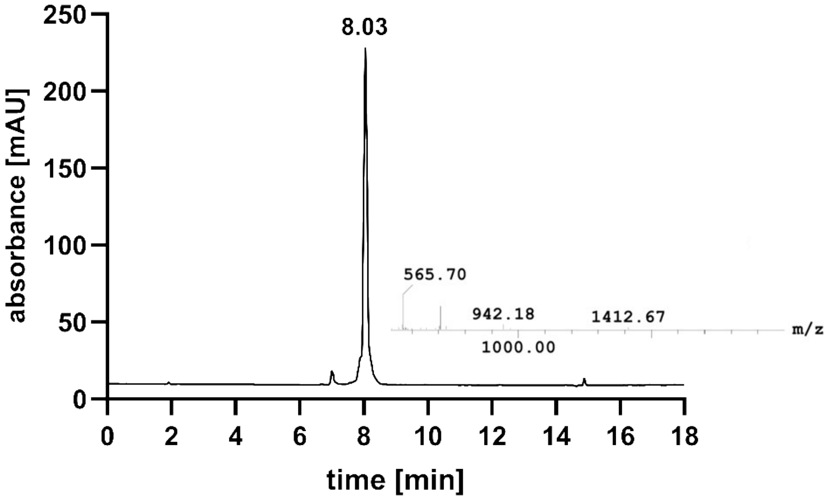

Analytical HPLC chromatogram of DBCO-SpyTag 14

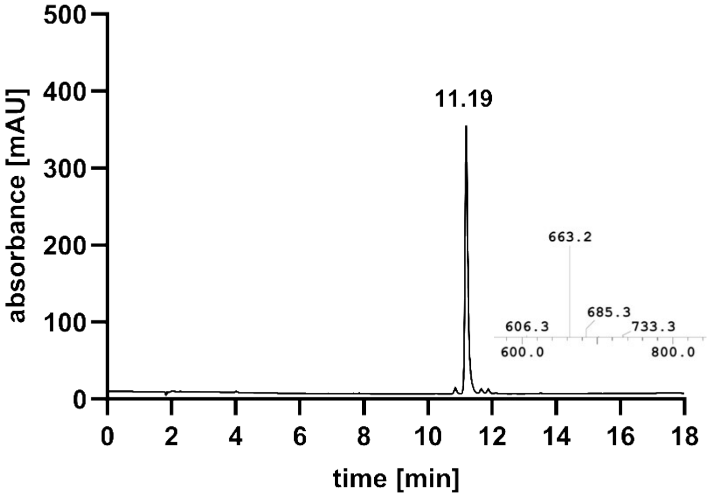

Analytical HPLC chromatogram of di-Fmoc compound 3 (crude)

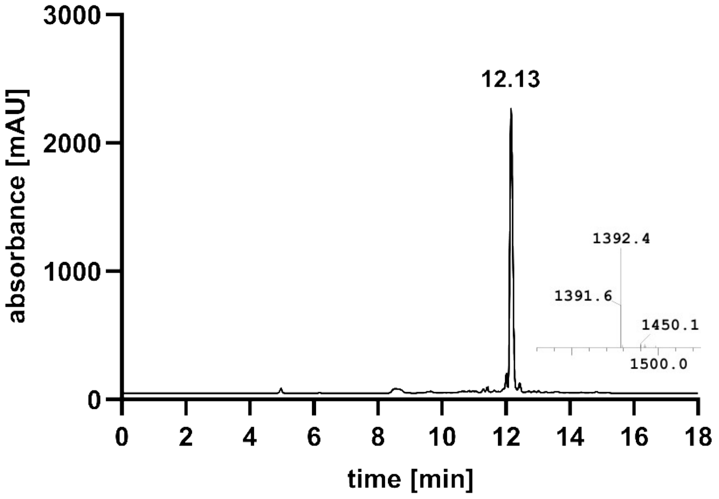

Analytical HPLC chromatogram of TetraFmoc compound 4 (crude)

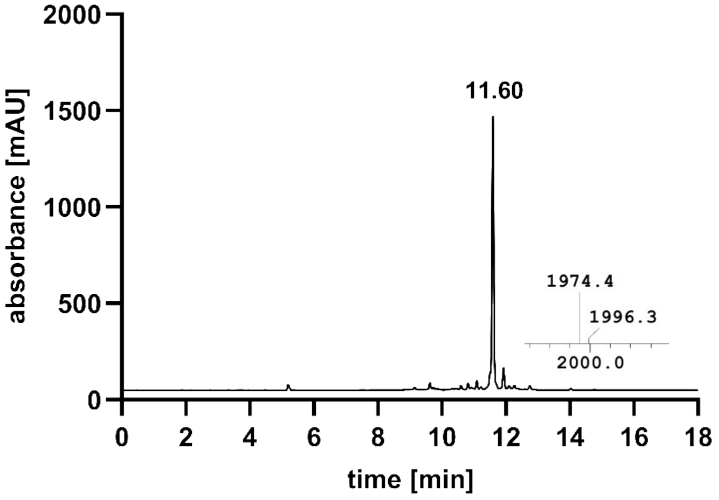

Analytical HPLC chromatogram of normal TetraFmoc-PEG compound S12 (crude)

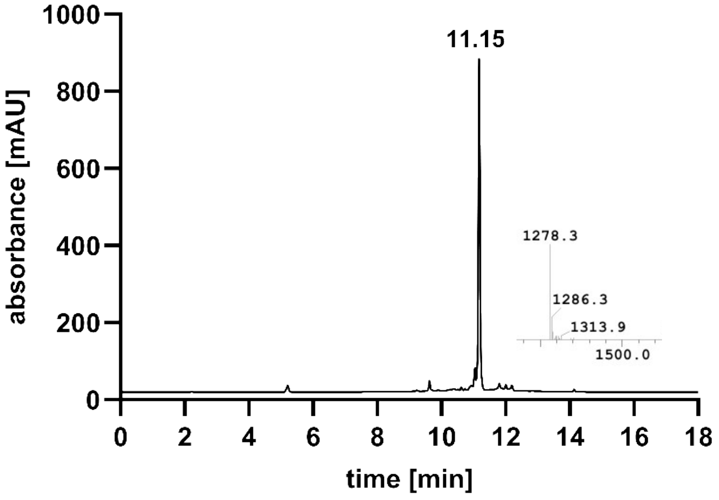

Analytical HPLC chromatogram of long TetraFmoc-PEG compound S13 (crude)

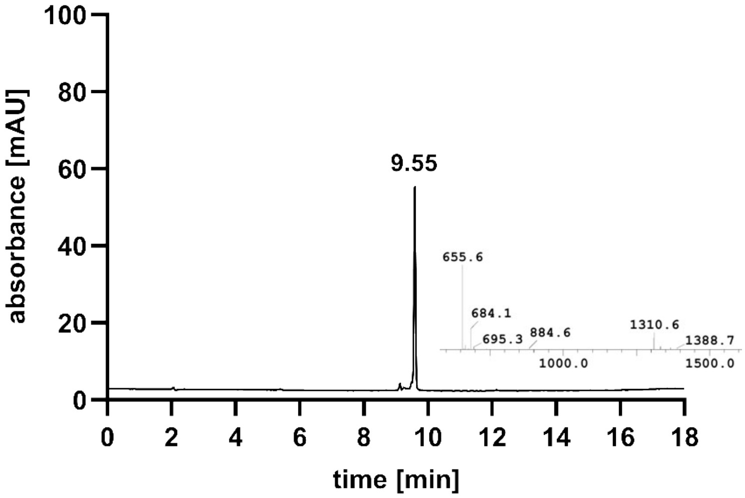

Analytical HPLC chromatogram of short TetraDVP acid 5

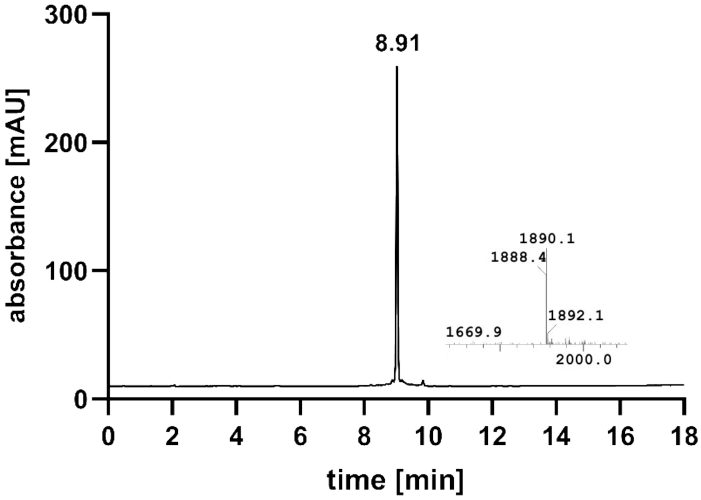

Analytical HPLC chromatogram of normal TetraDVP acid 6 (crude)

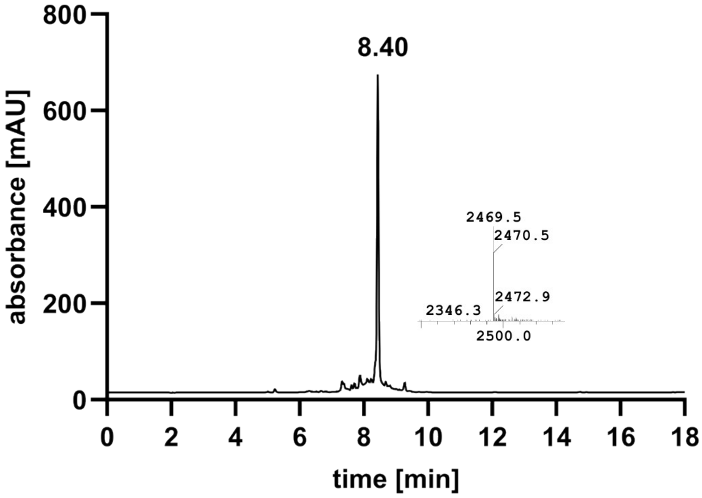

Analytical HPLC chromatogram of long TetraDVP acid 7 (crude; from 1g of resin)

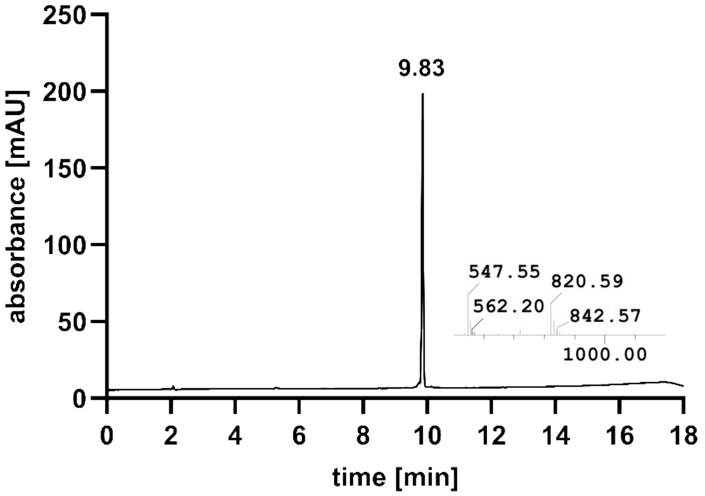

Analytical HPLC chromatogram of short TetraDVP azide 8

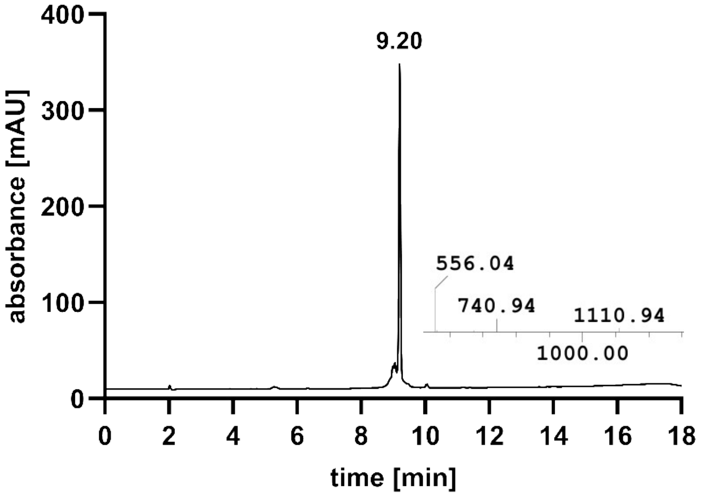

Analytical HPLC chromatogram of normal TetraDVP azide 9

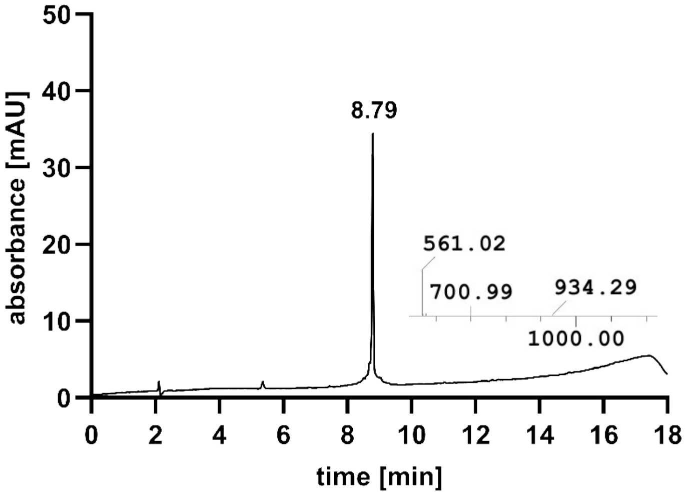

Analytical HPLC chromatogram of long TetraDVP azide 10

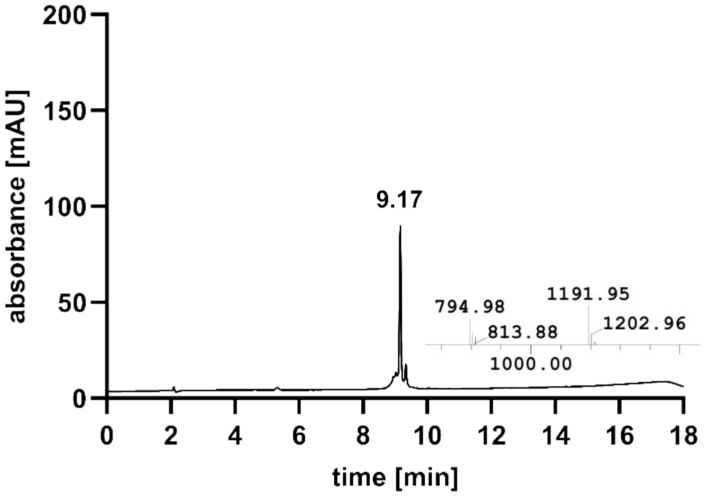

Analytical HPLC chromatogram of extended TetraDVP azide 11

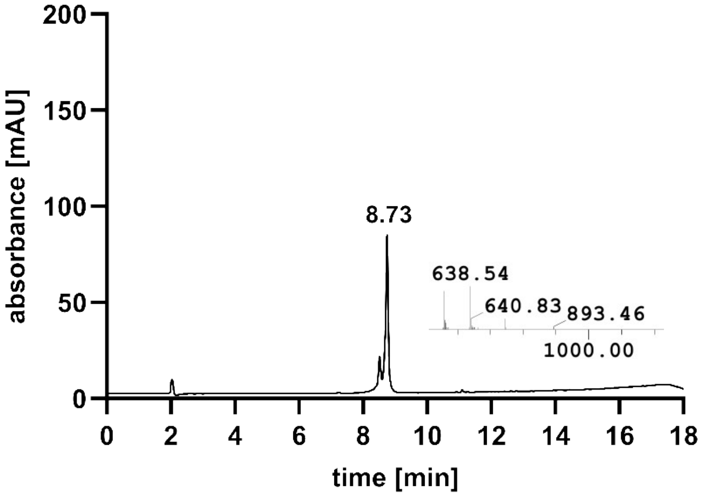

Analytical HPLC chromatogram of final TetraDVP-SpyTag conjugate 15

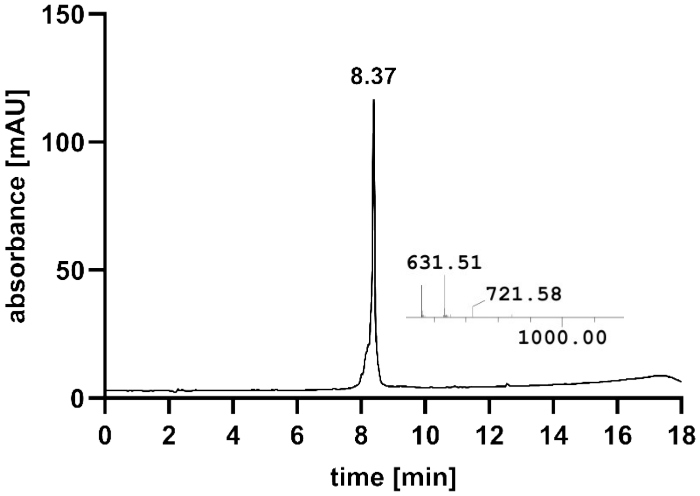

Analytical HPLC chromatogram of final TetraDVP-SpyTag conjugate 16

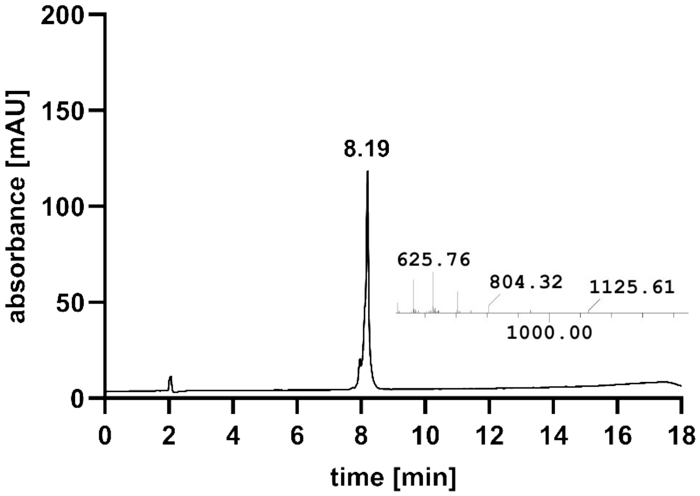

Analytical HPLC chromatogram of final TetraDVP-SpyTag conjugate 17

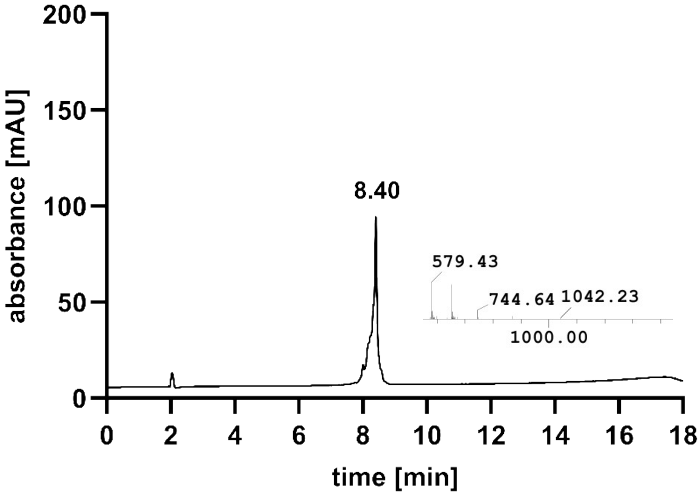

Analytical HPLC chromatogram of final TetraDVP-SpyTag conjugate 18

4. Bioconjugation

4.1. General bioconjugation procedure

To a 2.5 mg/mL solution of trastuzumab (F. Hoffmann-La Roche AG) in TBS buffer (25 mM Tris-HCl, 25 mM NaCl, 0.5 mM EDTA, pH 8, 100 µL) was added tris(2-carboxyethyl)phosphine hydrochloride (TCEP, 5 mM in TBS, 10 eq.) and the solution incubated for 1 h at 37 °C on a thermal shaker at 400 rpm. TetraDVP linker (2.5 mM in DMSO, 5 eq.) was added and the solution incubated for 23 h at 37 °C on a thermal shaker at 400 rpm. The final solution was filtered through a Zeba^{TM} spin desalting column (equilibrated into PBS buffer, 40,000 MW cut-off, Thermo-Fisher Scientific) and buffer exchanged into PBS via an Amicon-Ultra 15 diafiltration spin-concentrator (10,000 MW cut-off, Merck Millipore) to give a final antibody-conjugate concentration of up-to 100 µM.

The resulting antibody-conjugates were analysed *via* SDS-PAGE, LRMS, SEC, and HIC (see section 1.2). In all cases, no stability issues were observed for the antibodies during the bioconjugations or the resulting antibody-SpyTag conjugates.

4.2. Final conjugates bioconjugation

- ***Trastuzumab-TetraDVP-SpyTag Tras-17***

Synthesised according to the General Bioconjugation Procedure using TetraDVP conjugate **17**. Also carried out on a 500 µL scale for biological testing.

LRMS calcd. *m/z* $[M+H]^+$ 150,796; found $[M+H]^+$ 150,802.

- ***Durvalumab-TetraDVP-SpyTag Tras-17***

Synthesised according to the General Bioconjugation Procedure using TetraDVP conjugate **17**.

LRMS calcd. *m/z* $[M+H]^+$ 151,660; found $[M+H]^+$ 151,664.

- ***Brentuximab-TetraDVP-SpyTag Tras-17***

Synthesised according to the General Bioconjugation Procedure using TetraDVP conjugate **17**.

LRMS calcd. *m/z* $[M+H]^+$ 150,782; found $[M+H]^+$ 150,788.

- ***Cetuximab-TetraDVP-SpyTag Tras-17***

Synthesised according to the General Bioconjugation Procedure using TetraDVP conjugate **17**.

LRMS calcd. *m/z* $[M+H]^+$ 155,186; found $[M+H]^+$ 155,190.

- ***Gemtuzumab-TetraDVP-SpyTag Tras-17***

Synthesised according to the General Bioconjugation Procedure using TetraDVP conjugate **17**. Bioconjugation was not complete, therefore a further addition of TCEP (10 eq.) followed by TetraDVP (5 eq.) was added and the reaction incubated for a further 23 h. This was repeated for a total of 3 additions, over three 24 h cycles.

LRMS calcd. *m/z* $[M+H]^+$ 150,580; found $[M+H]^+$ 150,578.

4.3. Tras-17 conjugate analysis

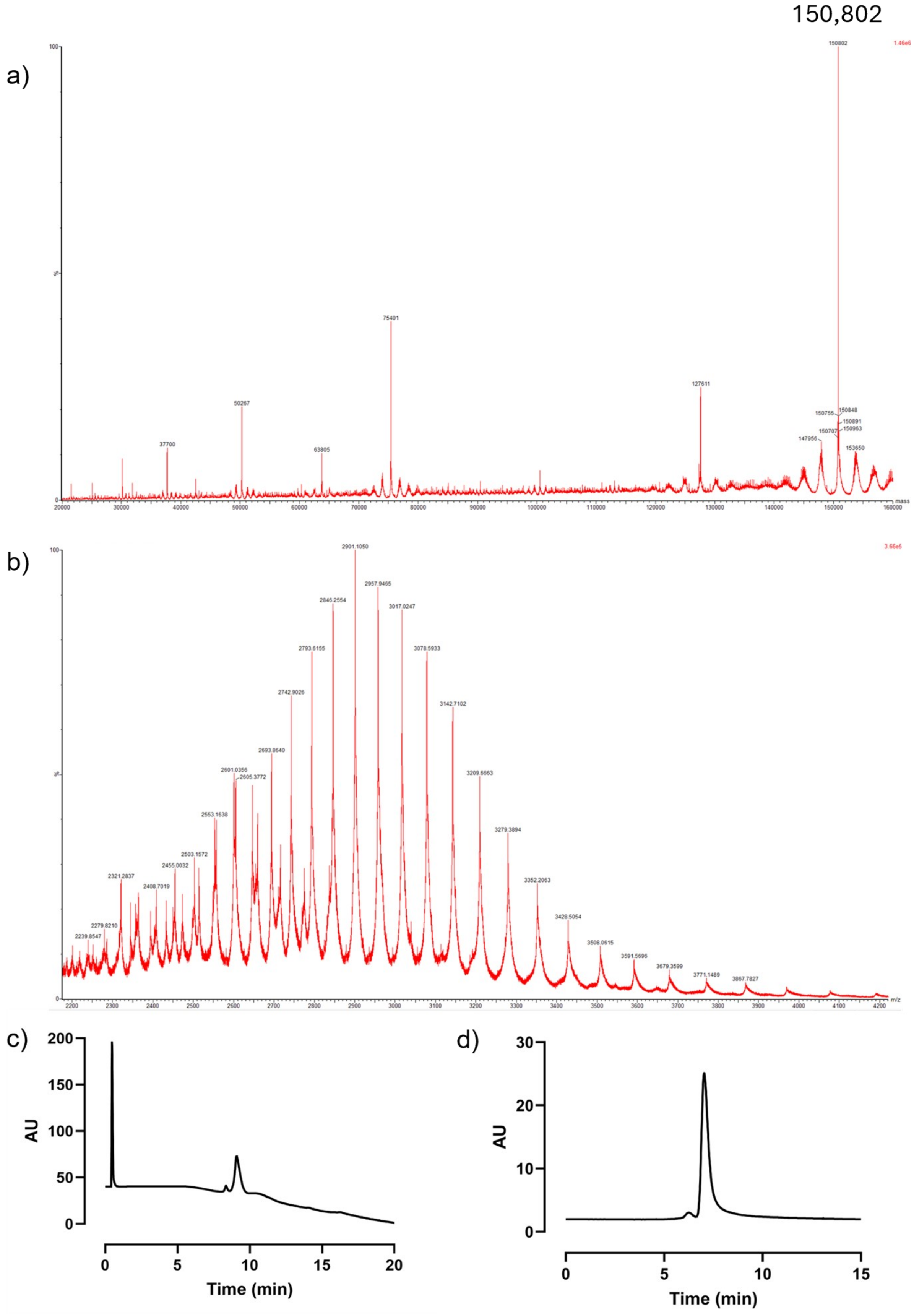

Figure S1: Analytical data for Trastuzumab-TetraDVP-SpyTag **Tras-17**. **a)** deconvoluted MS (2200 – 4000 *m/z*); intensity vs deconvoluted mass, peak height referenced to most intense signal. Full **Tras-17** $[M+H]^+$ 150,802 (expected: 150,796); light-heavy-heavy chain re-bridged with **17**, TCEP adduct $[M+H]^+$ 127,611 (expected: 127,601); full **Tras-17** $[M+2H]^{+2}$ 75,401; light-heavy-heavy chain re-bridged with **17**, TCEP adduct $[M+2H]^{+2}$ 63,805; full **Tras-17** $[M+3H]^{+3}$ 50,267; **b)** raw MS; intensity vs m/z; **c)** HIC analysis over 20 min; **d)** SEC analysis; 2.5% aggregation.

4.4. Dur-17 conjugate analysis

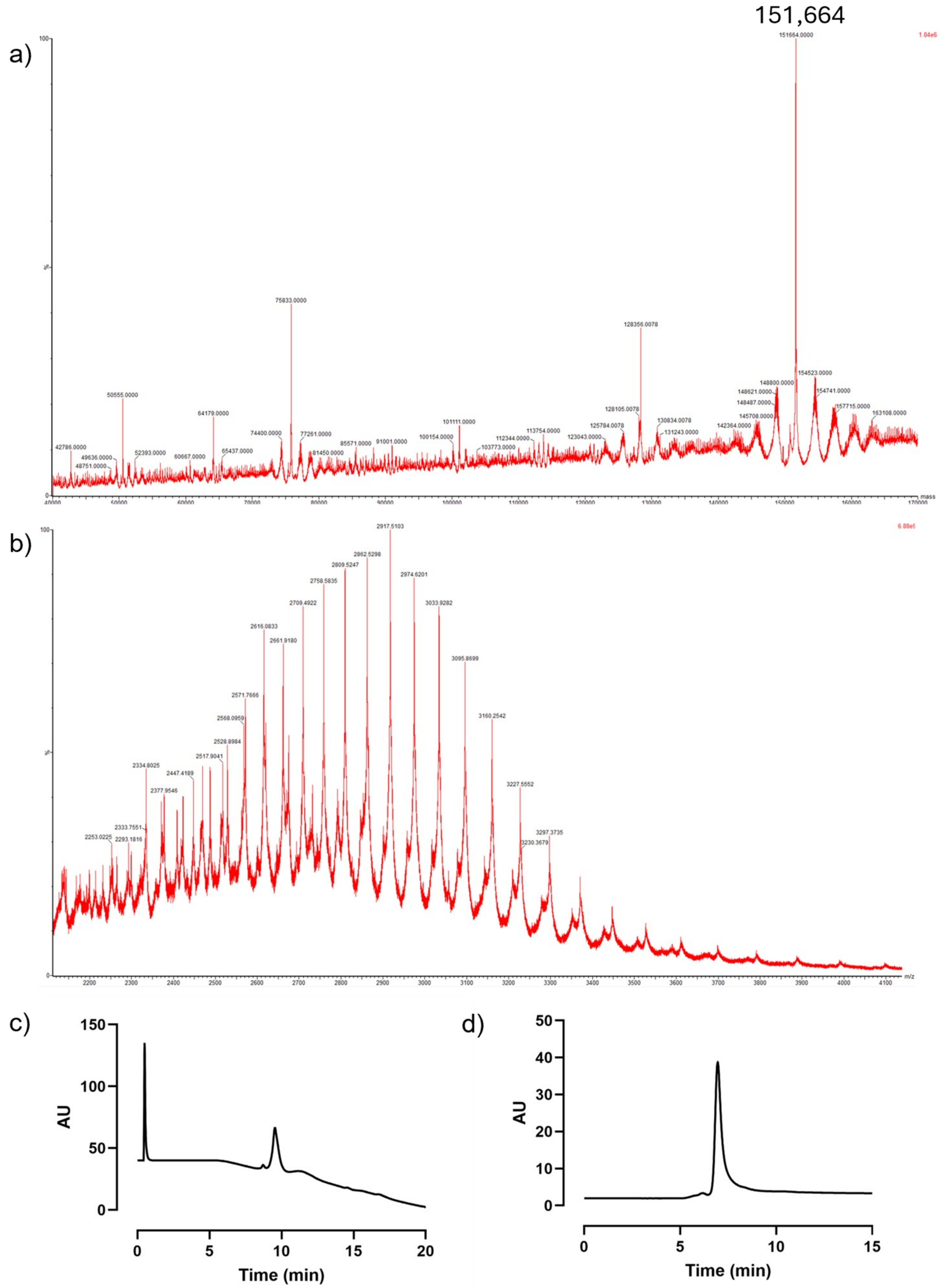

Figure S2: Analytical data for Durvalumab-TetraDVP-SpyTag **Dur-17**. **a)** deconvoluted MS (2200 – 4000 *m/z*); intensity vs deconvoluted mass, peak height referenced to most intense signal. Full **Dur-17** $[M+H]^+$ 151,664 (expected: 151,660); light-heavy-heavy chain re-bridged with **17**, TCEP adduct $[M+H]^+$ 128,356; full **Dur-17** $[M+2H]^{+2}$ 75,833; light-heavy-heavy chain re-bridged with **17**, TCEP adduct $[M+2H]^{+2}$ 64,179; full **Dur-17** $[M+3H]^{+3}$ 50,555; **b)** raw MS; intensity vs m/z; **c)** HIC analysis over 20 min; **d)** SEC analysis; 2.0% aggregation.

4.5. Bren-17 conjugate analysis

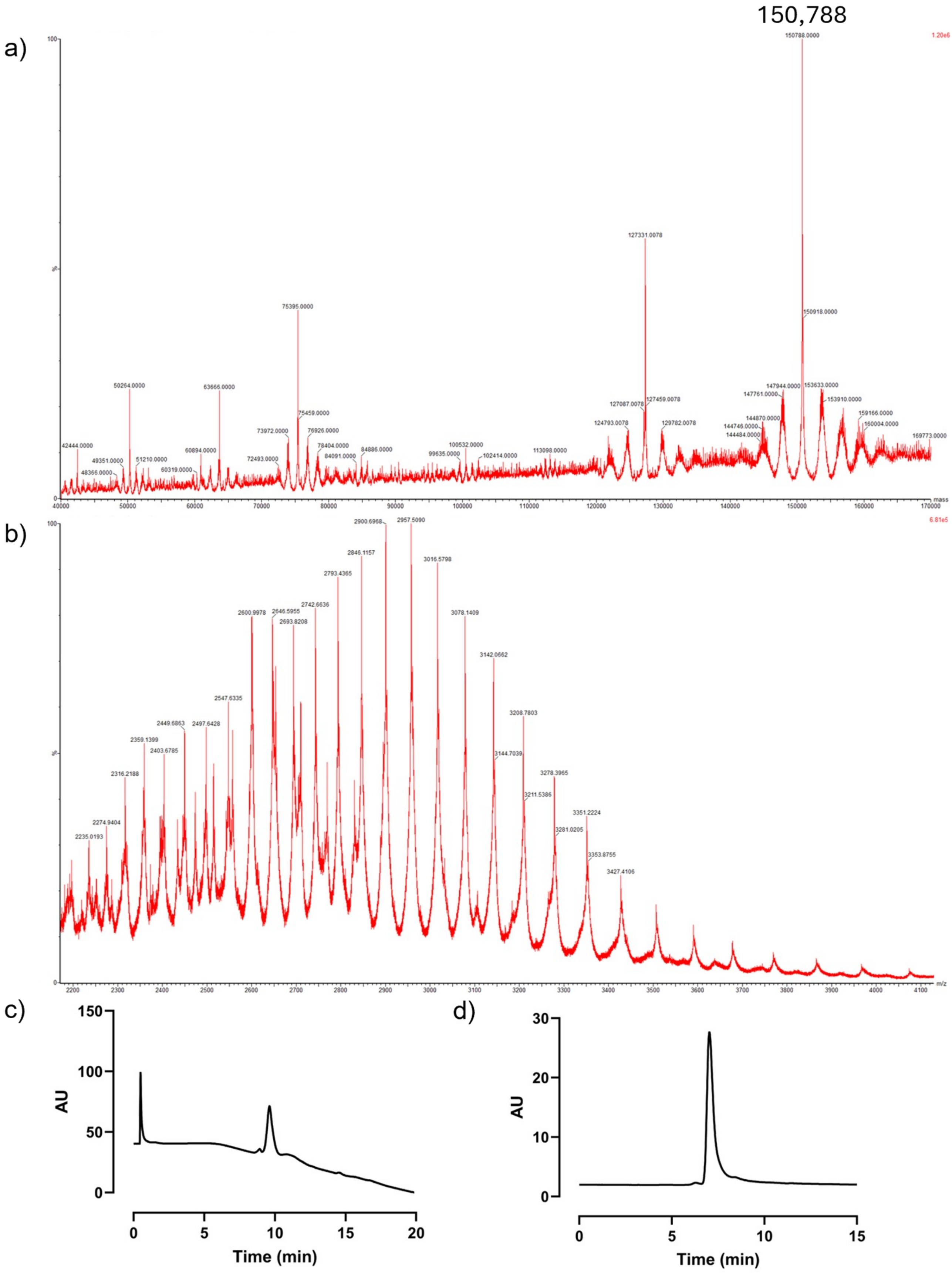

Figure S3: Analytical data for Brentuximab-TetraDVP-SpyTag **Bren-17**. **a)** deconvoluted MS (2200 – 4000 *m/z*); intensity vs deconvoluted mass, peak height referenced to most intense signal. Full **Bren-17** $[M+H]^+$ 150,788 (expected: 150,782); light-heavy-heavy chain re-bridged with **17**, TCEP adduct $[M+H]^+$ 127,331; full **Bren-17** $[M+2H]^{+2}$ 75,395; light-heavy-heavy chain re-bridged with **17**, TCEP adduct $[M+2H]^{+2}$ 63,665; full **Bren-17** $[M+3H]^{+3}$ 50,264; **b)** raw MS; intensity vs m/z; **c)** HIC analysis over 20 min; **d)** SEC analysis; 0.7% aggregation.

4.6. Cet-17 conjugate analysis

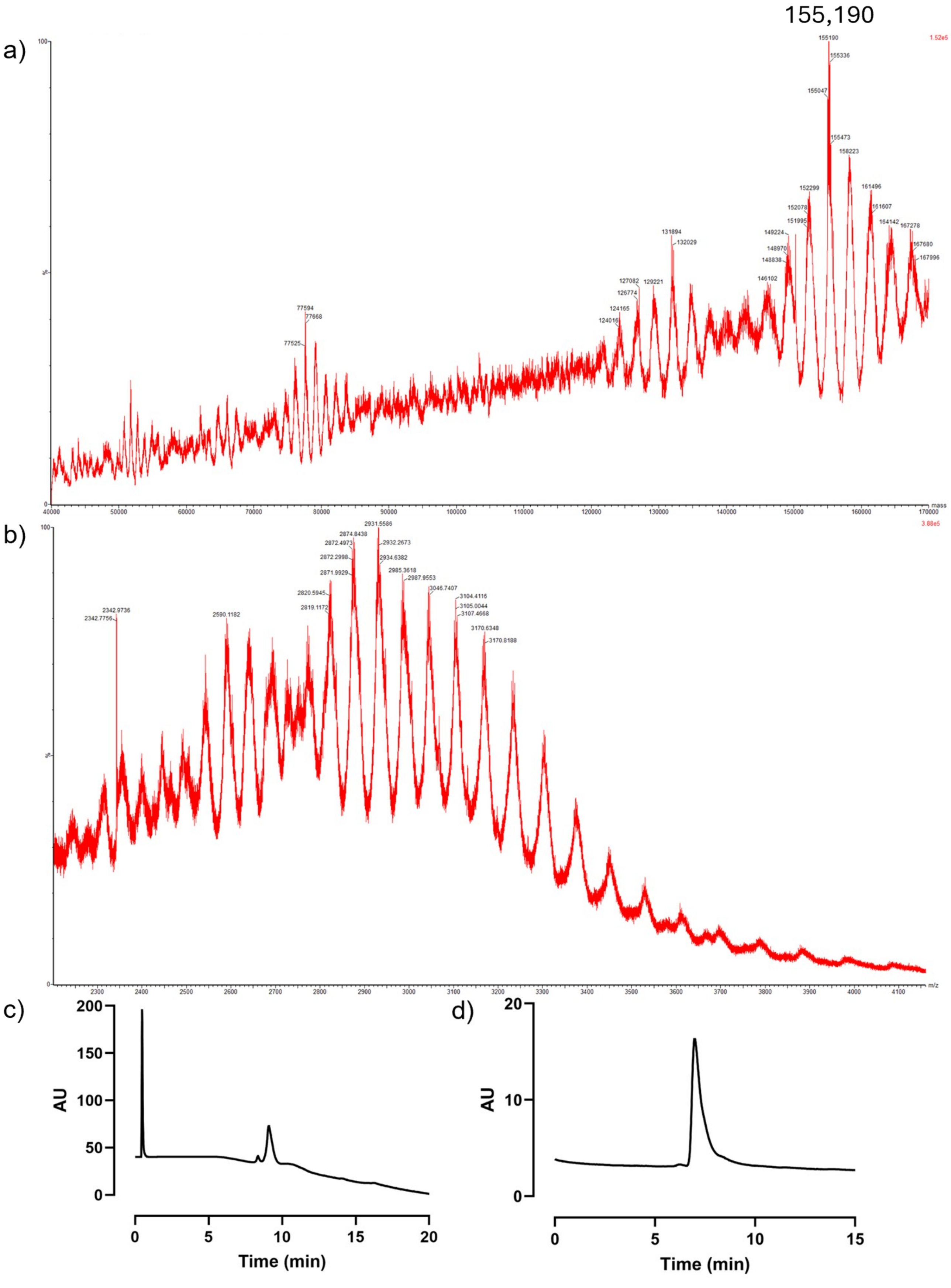

Figure S4: Analytical data for Cetuximab-TetraDVP-SpyTag **Cet-17**. **a)** deconvoluted MS (2200 – 4000 *m/z*); intensity vs deconvoluted mass, peak height referenced to most intense signal. Full **Cet-17** $[M+H]^+$ 155,190 (expected: 155,186); light-heavy-heavy chain re-bridged with **17**, TCEP adduct $[M+H]^+$ 129,221; full **Cet-17** $[M+2H]^{+2}$ 77,594; **b)** raw MS; intensity vs m/z; **c)** HIC analysis over 20 min; **d)** SEC analysis; 1.7% aggregation.

4.7. Gem-17 conjugate analysis

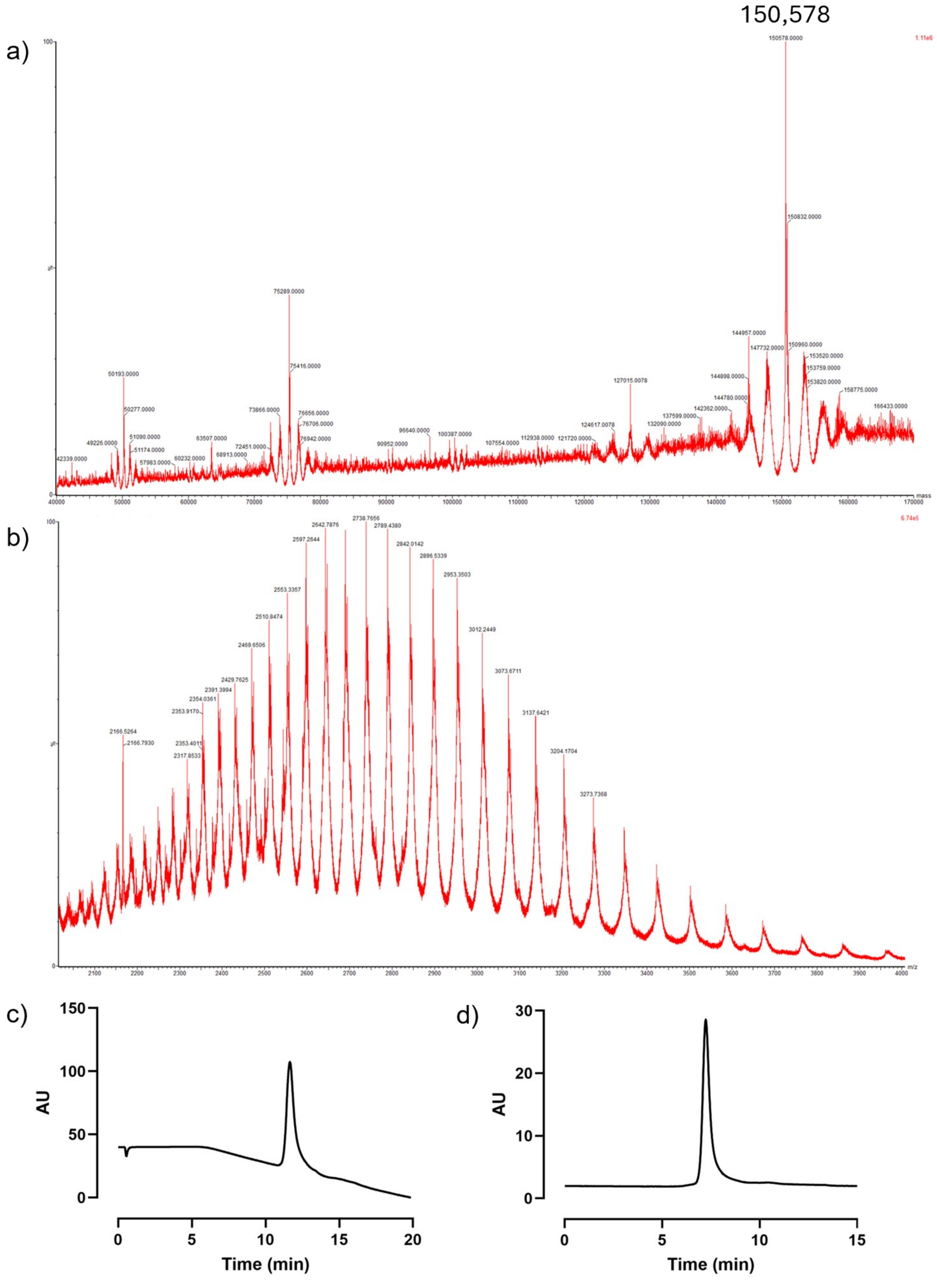

Figure S5: Analytical data for Gemtuzumab-TetraDVP-SpyTag **Gem-17**. **a)** deconvoluted MS (2200 – 4000 *m/z*); intensity vs deconvoluted mass, peak height referenced to most intense signal. Full **Gem-17** $[M+H]^+$ 150,578 (expected: 150,580); light-heavy-heavy chain re-bridged with **17**, TCEP adduct $[M+H]^+$ 127,015; full **Gem-17** $[M+2H]^{+2}$ 75,289; light-heavy-heavy chain re-bridged with **17**, TCEP adduct $[M+2H]^{+2}$ 63,507; full **Gem-17** $[M+3H]^{+3}$ 50,193; **b)** raw MS; intensity vs m/z; **c)** HIC analysis over 20 min; **d)** SEC analysis; <1% aggregation.

4.8. Bioconjugation optimisation

- ***HIC comparison of TetraDVP linker size and the effect of EDTA and buffer type***

a)

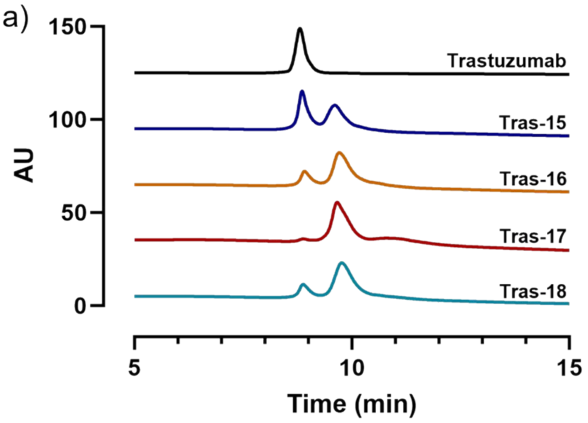

Antibody-Conjugate	Conversion
Tras-15	50%
Tras-16	80%
Tras-17	97%
Tras-18	80%

b)

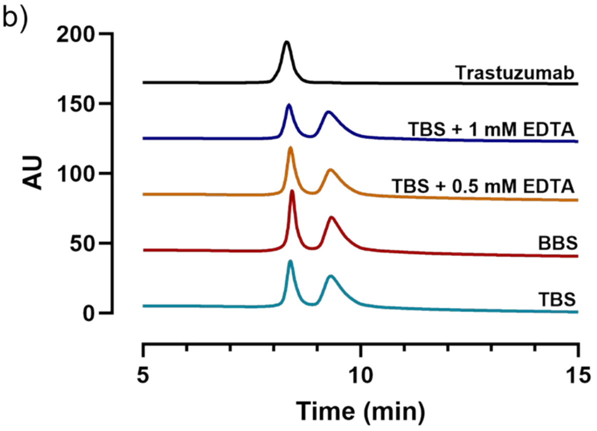

Antibody-Conjugate	Conversion
Standard TBS	57%
Standard BBS	54%
TBS + 0.5 mM EDTA	61%
TBS + 1 mM EDTA	51%

Figure S6: a) HIC analysis of the different TetraDVP linkers on trastuzumab. Conjugations run in TBS (1 x, 2.5 mg/mL trastuzumab, 100 µL) with 10 eq. TCEP for 1 h then 5 eq. of TetraDVP conjugates **15**, **16**, **17**, or **18** were added and incubated at 37 °C for 23 h; repeated daily for 3 days. The conversion was assessed by measuring peak area of the conjugate against unreacted trastuzumab.
b) Effect of changing conjugation buffer and EDTA concentration. Conjugations run in TBS/BBS (including 0.5 mM EDTA, 2.5 mg/mL trastuzumab, 100 µL), or with additional EDTA added, with 10 eq. TCEP for 1 h then 5 eq. TetraDVP conjugate **16** added and incubated at 37 °C for 23 h. The conversion was assessed by measuring peak area of the conjugate against unreacted trastuzumab. (BBS = Borate Buffered Saline (25 mM boric acid, 25 mM NaCl, 0.5 mM EDTA, pH 8.0)

To reach high conversion, these initial bioconjugation reactions required repeated reagent addition. Increasing the equivalents of TetraDVP, concentration, or time did not improve the conversion. Therefore, it was postulated that the TetraDVP linkers themselves were hindering the conjugation. It was found that the purity of the linkers greatly affected the rate, with purer linkers allowing >95% bioconjugation after a single reagent addition, compared to requiring 3 x 24 h reagent addition cycles. All further IgG1 studies in this work utilise a single 24 h bioconjugation.

▪ ***Gemtuzumab Bioconjugation***

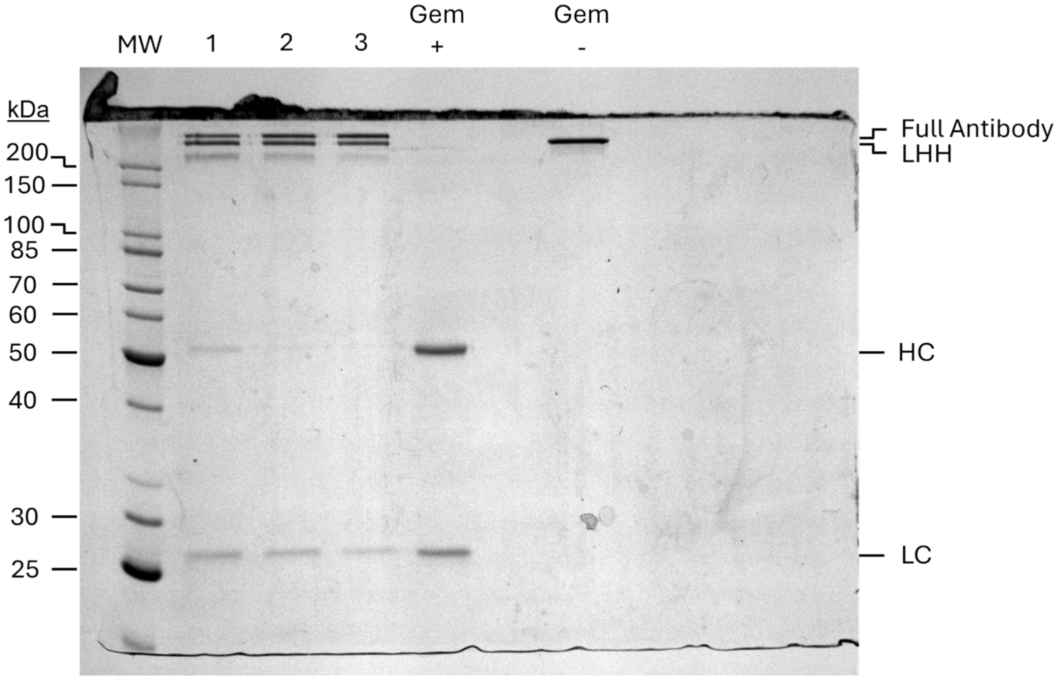

Figure S7: SDS-PAGE comparing conjugation of gemtuzumab with TetraDVP-SpyTag **17** over 3 days; with Coomassie blue staining. Conjugations run in TBS (1 x, 2.5 mg/mL trastuzumab, 100 µL) with 10 eq. TCEP for 1 h then TetraDVP-SpyTag **17** (5 eq.) was added and incubated at 37 °C for 23 h; repeated for further days as required. Lane number refers to the number of reaction addition cycles carried out. 3 reagent addition cycles gave near complete conversion. All analytes run in a reducing stain. MW = Molecular weight ladder with values given in kDa; Gem.= gemtuzumab; LC = native antibody light chain; HC = native antibody heavy chain; LHH = re-bridged antibody light-heavy-heavy chains. +/- indicates with or without reducing stain.

▪ ***Original SDS-PAGE gel form Figure 3B***

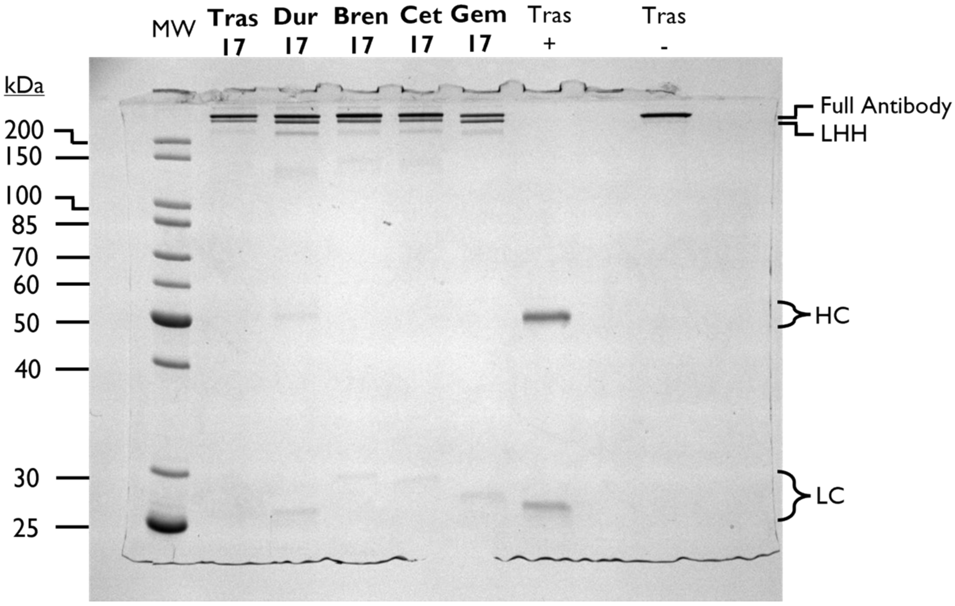

Figure S8: Uncropped SDS-PAGE from Figure 3B with Coomassie blue staining. Conjugations run in TBS (1 x, 2.5 mg/mL trastuzumab, 100 µL) with 10 eq. TCEP for 1 h then TetraDVP-SpyTag **17** (5 eq.) was added and incubated at 37 °C for 23 h. All analytes run in a reducing stain. MW = Molecular weight ladder with values given in kDa; Tras.= trastuzumab; LC = native antibody light chain; HC = native antibody heavy chain; LHH = re-bridged antibody light-heavy-heavy chains. +/- indicates with or without reducing stain.

5. Biology

- ***Plasmids***

o pDEST14-SpyCatcher003 (GenBank Accession no. MN433887)	https://www.ncbi.nlm.nih.gov/nuccore/MN433887
Addgene plasmid ID 133447	https://www.addgene.org/133447
o pET28-SpyCatcher002-MBP (GenBank Accession no. PP341225)	https://www.ncbi.nlm.nih.gov/nuccore/PP341225
o pDEST14-DoubleCatcher (SpyCatcher003-$(GSG)_3$-SpyCatcher003-TEVs-SpyTag003DA); GenBank Accession no. PP341218	https://www.ncbi.nlm.nih.gov/nuccore/PP341218
Addgene plasmid ID 216284	https://www.addgene.org/216284/
o pET28a-nanoHER2-SpyTag003 (GenBank Accession no. PP341234)	https://www.ncbi.nlm.nih.gov/nuccore/PP341234
Addgene plasmid ID 216312	https://www.addgene.org/216312/
o pJ404-SpyCatcher003-sfGFP (GenBank Accession No. MN433889)	https://www.ncbi.nlm.nih.gov/nuccore/MN433889
Addgene plasmid ID 133449	https://www.addgene.org/133449

Plasmids have been described in literature.[4,5]

- ***Isopeptide bond formation assays***

All reactions were carried out in PBS pH 7.4. Trastuzumab-TetraDVP-SpyTag003 **Tras-17** (1 µM) was incubated with either SpyCatcher003 (5 µM), SpyCatcher002-MBP (5 µM) or DoubleCatcher (5µM), in PBS pH 7.4 at 37 °C for 4 h or 24 h, as indicated. SpyCatcher003, SpyCatcher002-MBP or Double Catcher were purified as previously by Ni-NTA, following *Escherichia coli* expression. [5] At each time-point or end-point indicated, reactions were quenched by adding 6 × SDS loading buffer [0.23 M Tris-HCl pH 6.8, 24% (v/v) glycerol, 120 µM bromophenol blue, 0.23 M SDS], with subsequent heating at 99 °C for 3 min in a Bio-Rad C1000 thermal cycler. Reactions were analysed by SDS-PAGE (NuPAGE 4-12% Bis-Tris, ran in NuPAGE MOPS SDS Running Buffer (Invitrogen)) followed by Coomassie staining.

- ***Cell culture***

SKBR3 cells (HTB-30; a HER2-positive breast cancer cell line) and MDA-MB-468 (HTB-132; a HER2-negative breast cancer cell line) were purchased from ATCC. Both cell-lines were grown in complete DMEM: Dulbecco's Modified Eagle Medium–high glucose (DMEM) supplemented with 10% (v/v) foetal bovine serum (FBS), 100 U/mL penicillin and 100 µg/mL streptomycin (1 × pen/strep; Sigma-Aldrich), 1% (v/v) GlutaMAX (Thermo Fisher) at 37 °C and 5% (v/v) CO_2. Cells were passaged at 70-80% confluency and were sub-cultured for fewer than 2 months. Cell-lines were routinely validated as mycoplasma-negative by PCR.

- ***Flow cytometry***

Trastuzumab-TetraDVP-SpyTag003 **Tras-17** (2 µM) and nanoHER2-SpyTag003 (2 µM) were incubated with SpyCatcher003-sfGFP (2 µM) in HEPES Buffered Saline (HBS; 50 mM HEPES + 150 mM NaCl) pH 7.2 supplemented with 10% (v/v) FBS at 37 °C for 1 h. For negative controls, SpyTagged binders or SpyCatcher003-sfGFP alone were incubated in HBS + 10% (v/v) FBS at 2 µM. SKBR3 and MDA-MB-468 cells were grown in complete DMEM until 80% confluency, when they were trypsinized and resuspended in HBS pH 7.2 + 10% (v/v) FBS to a final concentration of 4×10^6 cells/mL. Cells were aliquoted into 96-well V-bottom plates (Corning) (50 µL, 2×10^5 cells, per well), centrifuged at 125 *g* at 4 °C for 3 min, and resuspended in 50 µL of the indicated pre-incubated binder:SpyCatcher003-sfGFP mixture. 'Unstained' cells were resuspended in HBS + 10% (v/v) FBS alone. Cells were incubated with binder-fluorophore mixtures for 30 min at 37 °C and 5% (v/v) CO_2, before washing twice by centrifugation at 125 *g*, 4 °C, 3 min, and resuspension in 200 µL HBS, except for the final wash step when cells were resuspended in 150 µL FACS buffer [HBS + 1% (w/v) Bovine Serum Albumin + 0.1% (w/v) sodium azide]. Cells were kept on ice and 5 minutes prior to flow cytometric analysis eBioscience™ 7-AAD Viability Staining Solution (Invitrogen, Cat no. 00-6993-50) was added to each well at 2.5 µL per million cells to exclude non-viable cells from analyses.

Labelled cells were then analysed by flow cytometry using a CytoFLEX LX flow cytometer (Beckman Coulter), with at least 10,000 events acquired per sample. Compensation matrices were calculated from unstained or single-stained compensation controls:

unstained cells, heat-treated (70 °C, 30 min) cells stained with eBioscience™ 7-AAD Viability Staining Solution, and cells incubated with nanoHER2:SpyCatcher003-sfGFP (2 μM) only. Compensation matrices were calculated using the CytExpert software (Beckman Coulter) and applied to sample data to compensate for overlapping spectra. FCS data files were subsequently analysed using FlowJo v10.10.0 software (BD Biosciences).

- ***Original SDS-PAGE from Figure 2C***

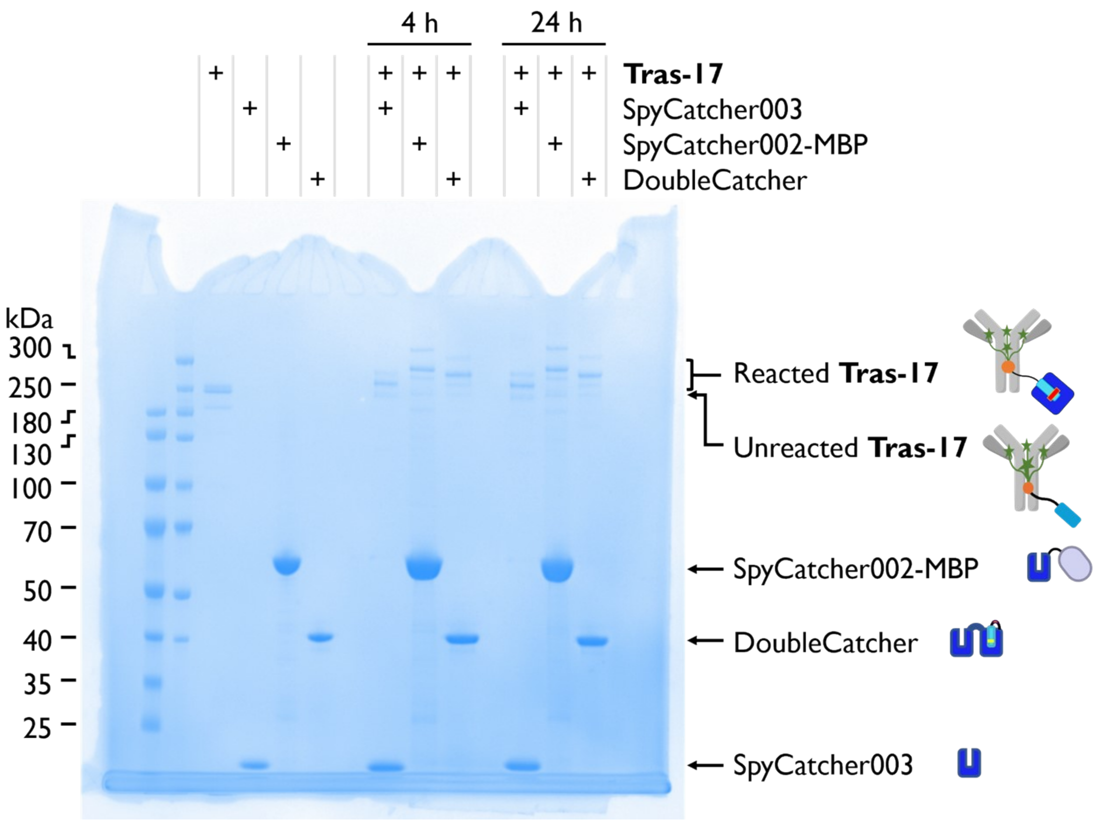

Figure S9: Uncropped SDS-PAGE gel from Figure 2C with Coomassie blue staining.

6. NMR spectra

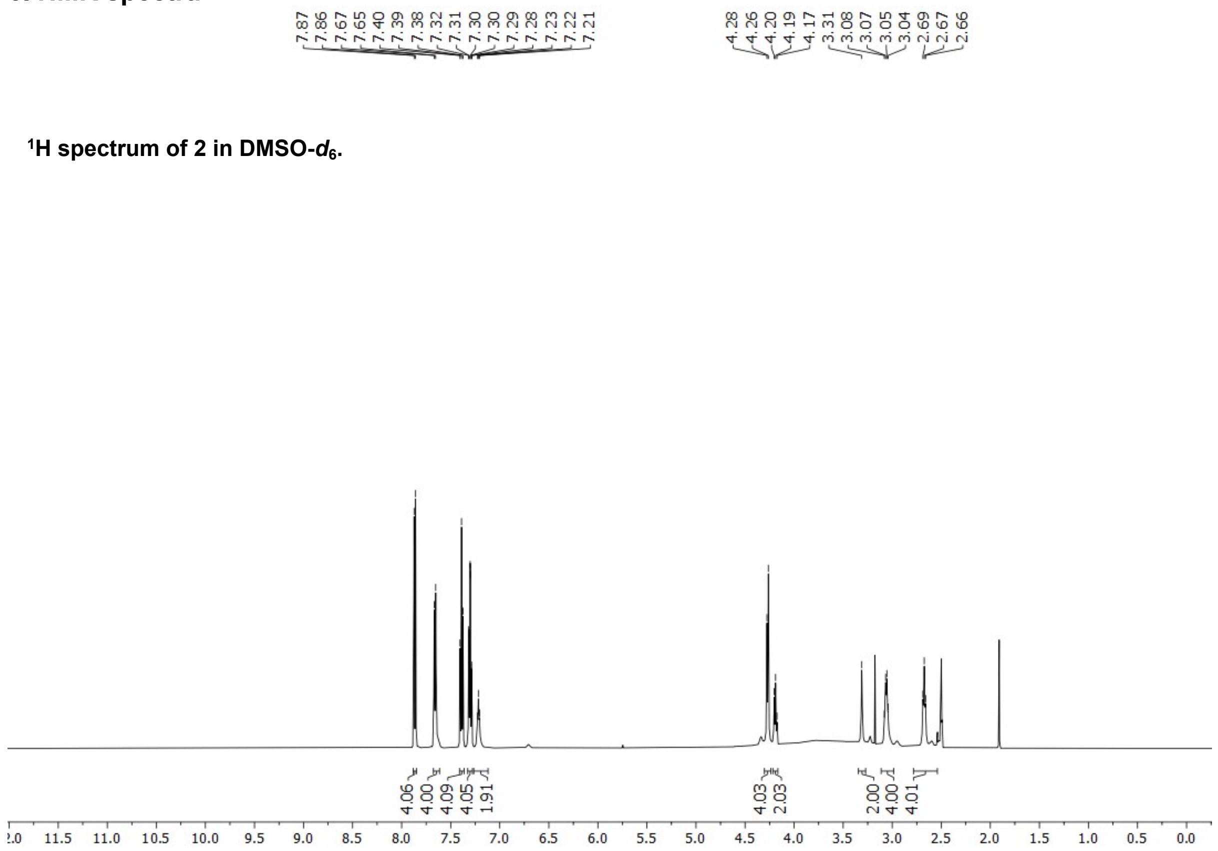

^{1}H spectrum of 2 in DMSO-d_6.

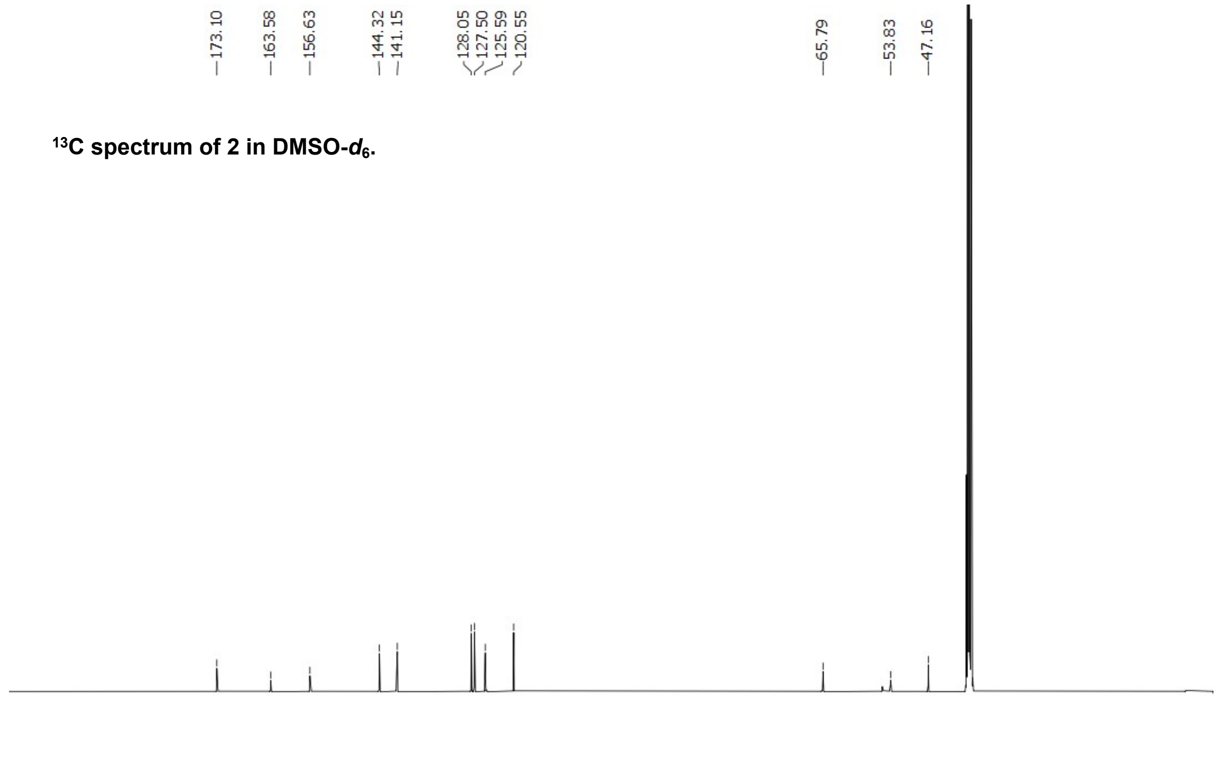

^{13}C spectrum of 2 in DMSO-d_6.

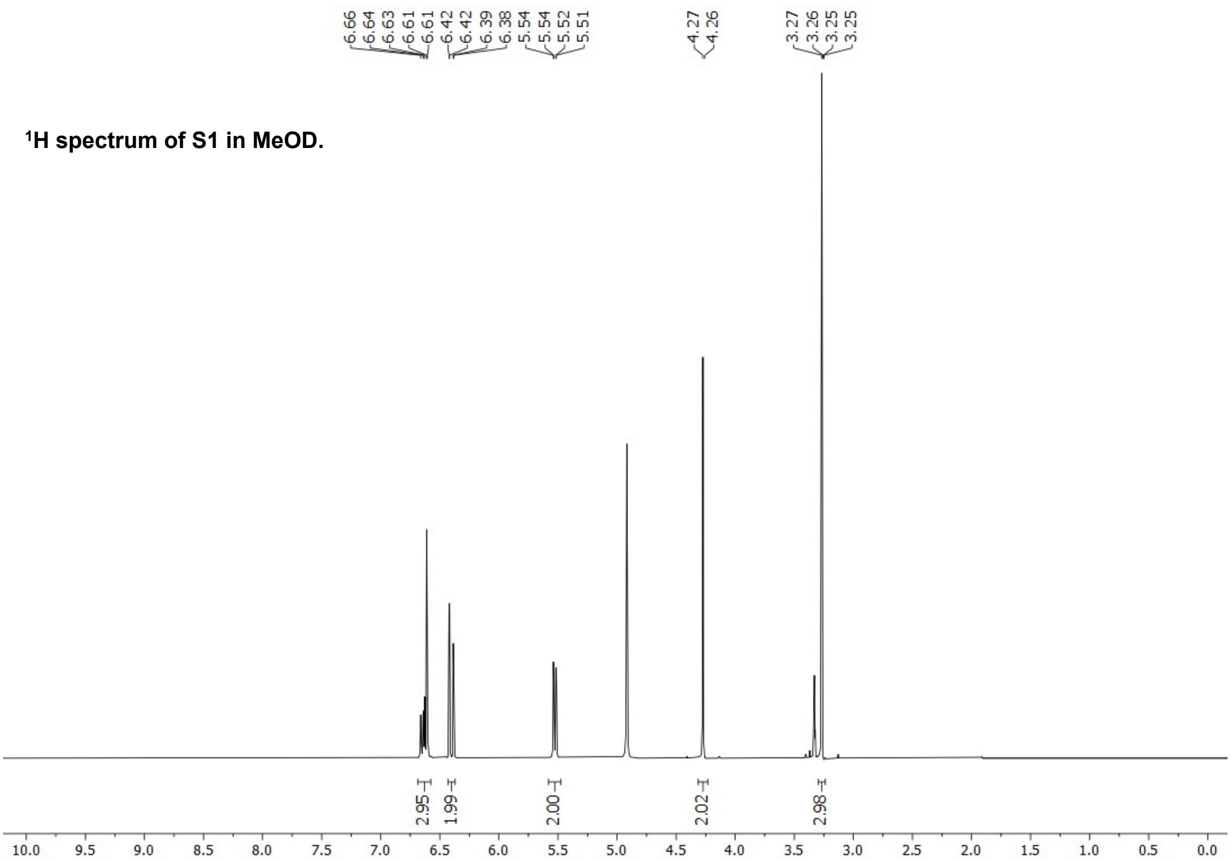

^{1}H spectrum of S1 in MeOD.

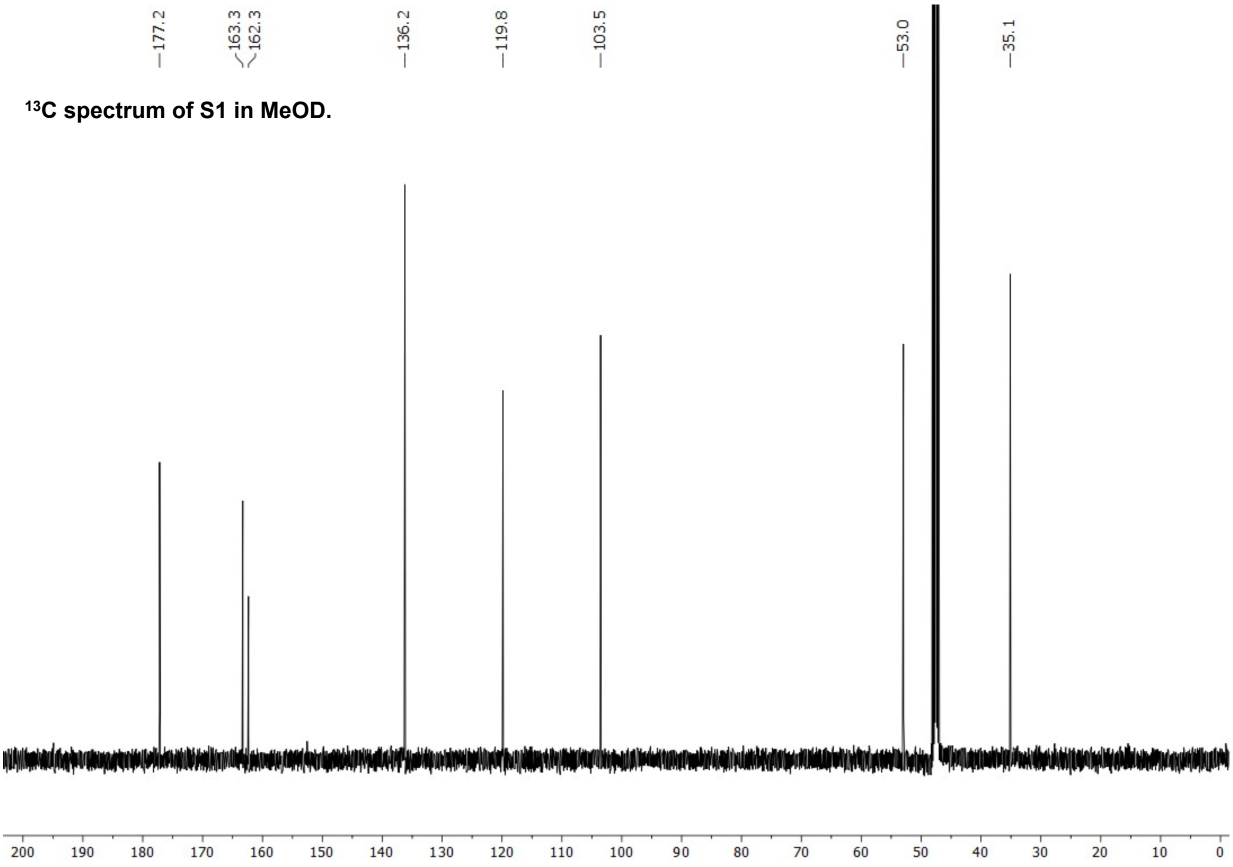

^{13}C spectrum of S1 in MeOD.

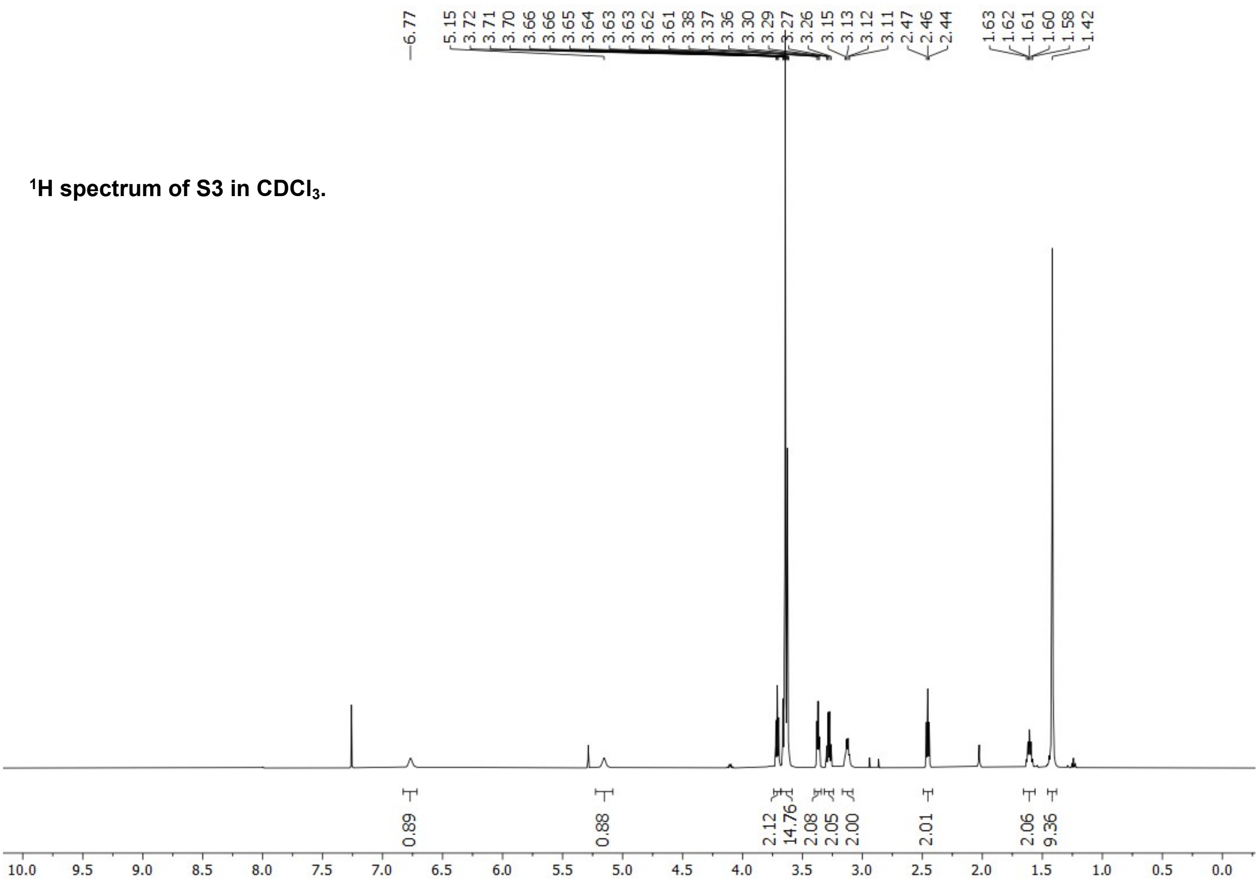

^{1}H spectrum of S3 in $CDCl_3$.

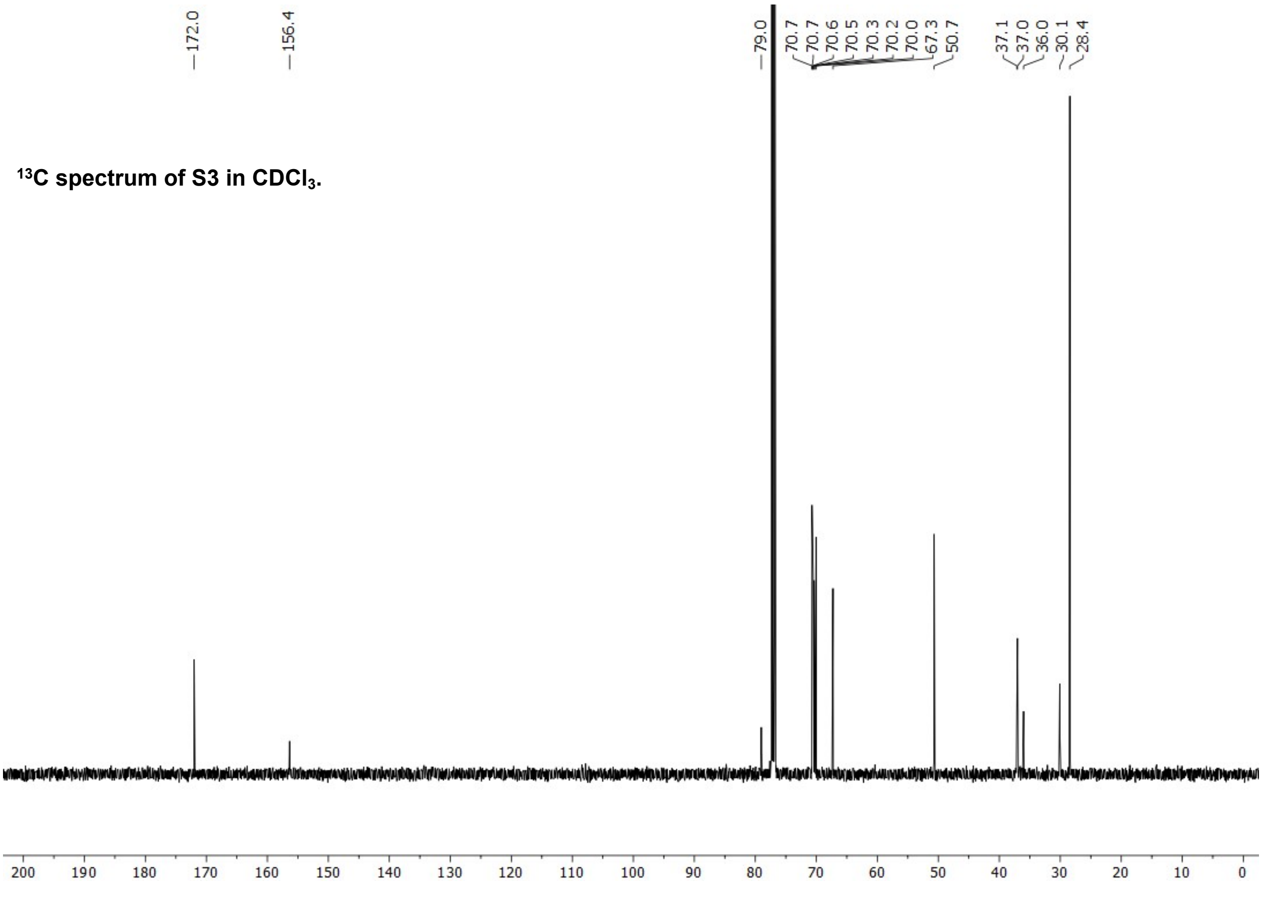

^{13}C spectrum of S3 in $CDCl_3$.

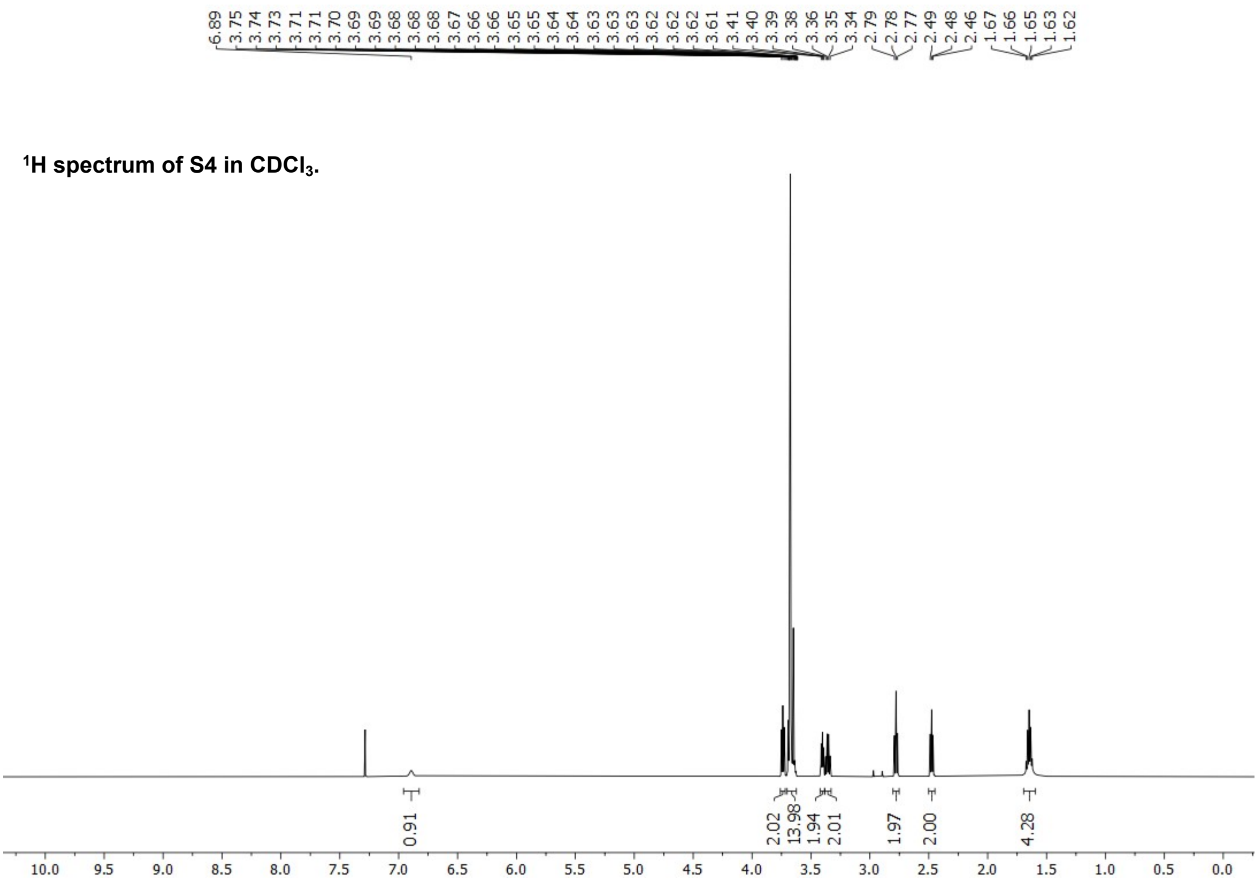

^{1}H spectrum of S4 in $CDCl_3$.

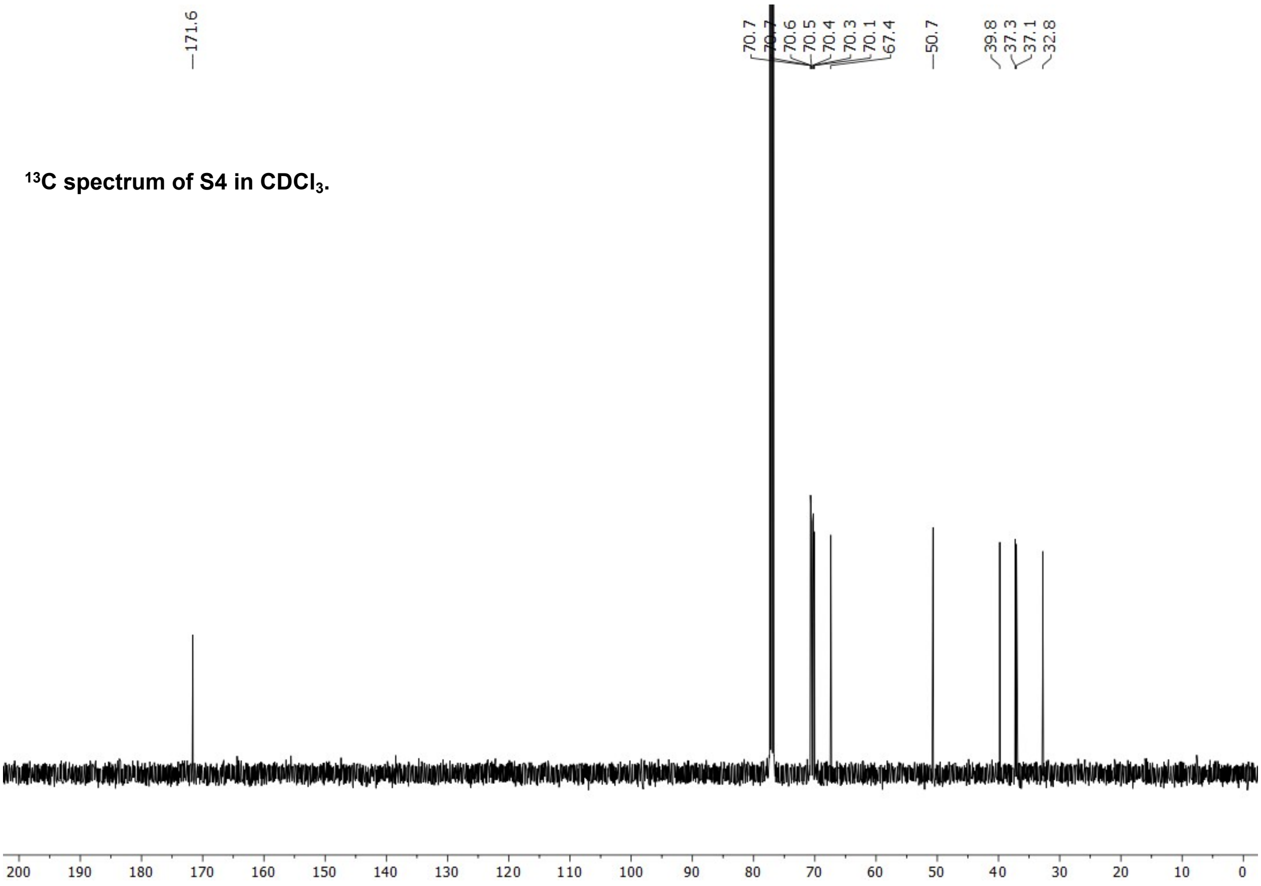

^{13}C spectrum of S4 in $CDCl_3$.

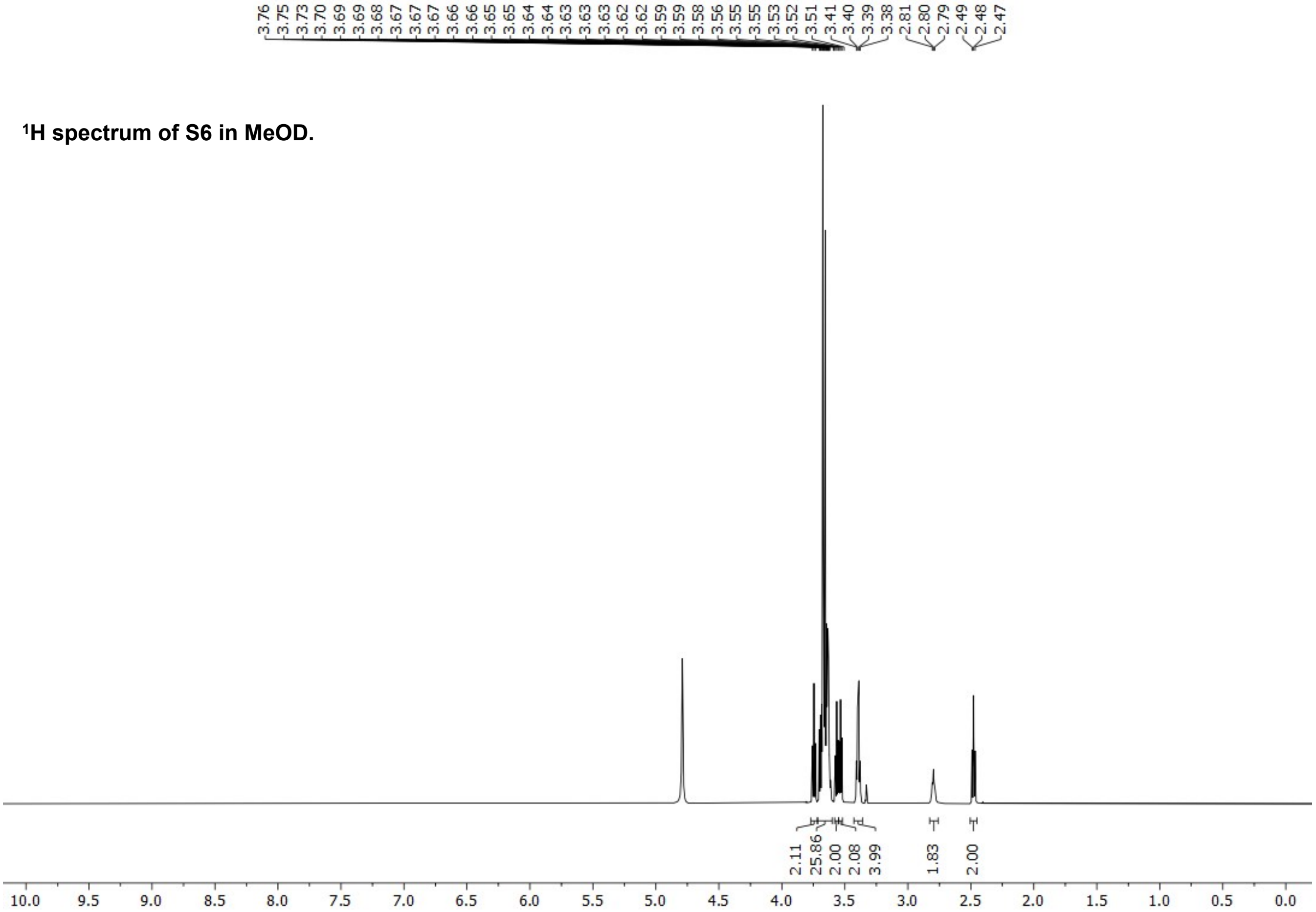

^{1}H spectrum of S6 in MeOD.

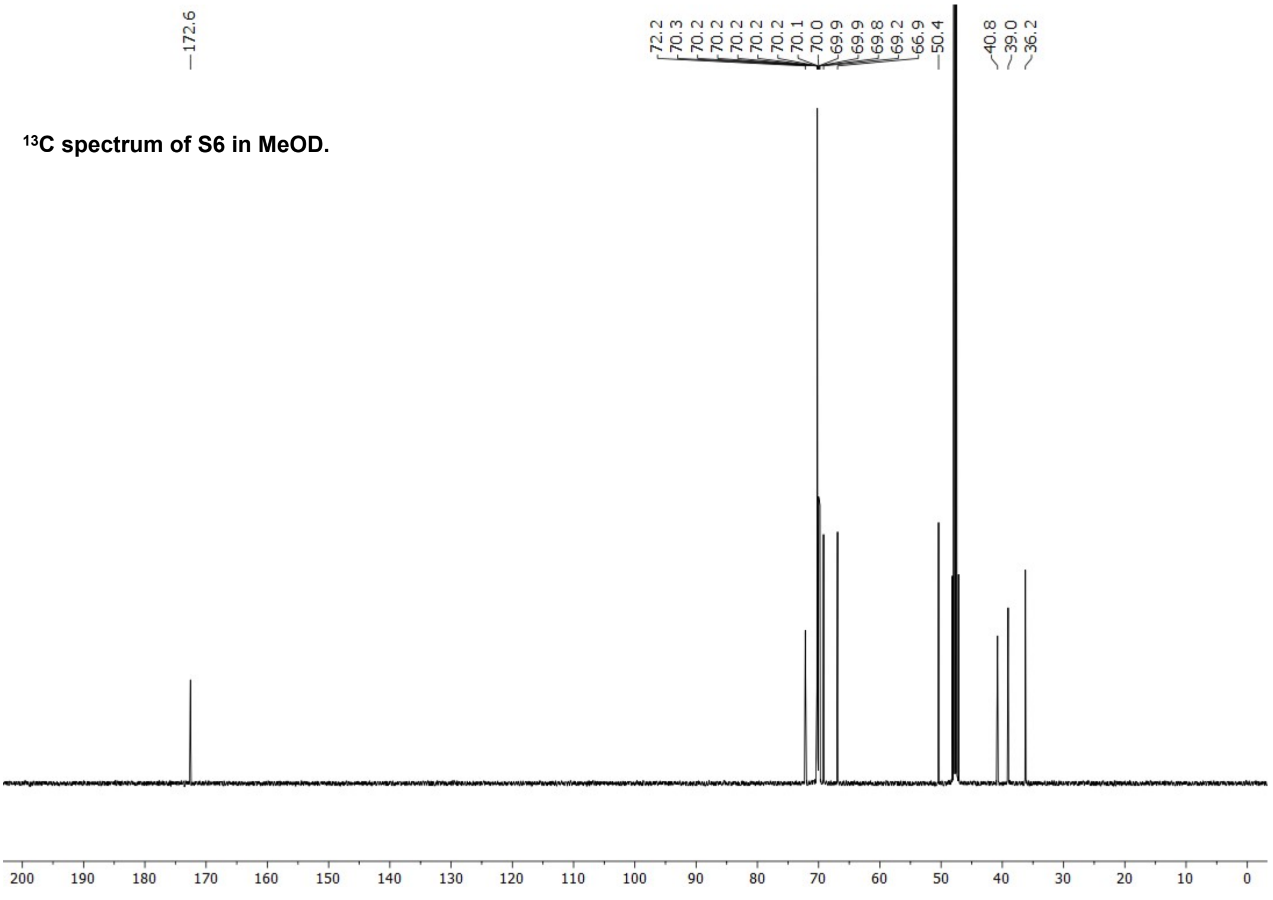

^{13}C spectrum of S6 in MeOD.

Crude ¹H spectrum of TetraDVP-acid 7 in DMSO-d_6.

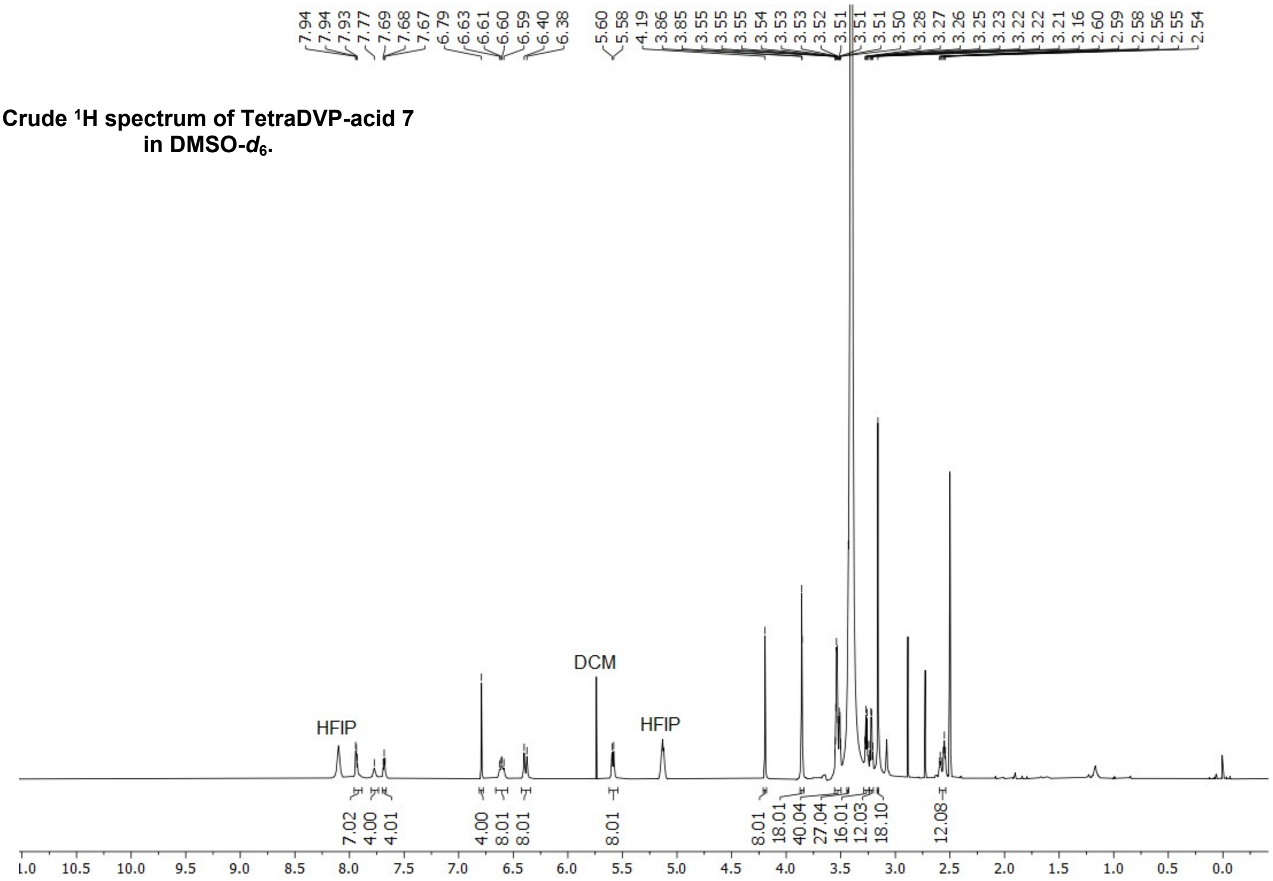

Crude ¹³C spectrum of TetraDVP-acid 7 in DMSO-d_6.

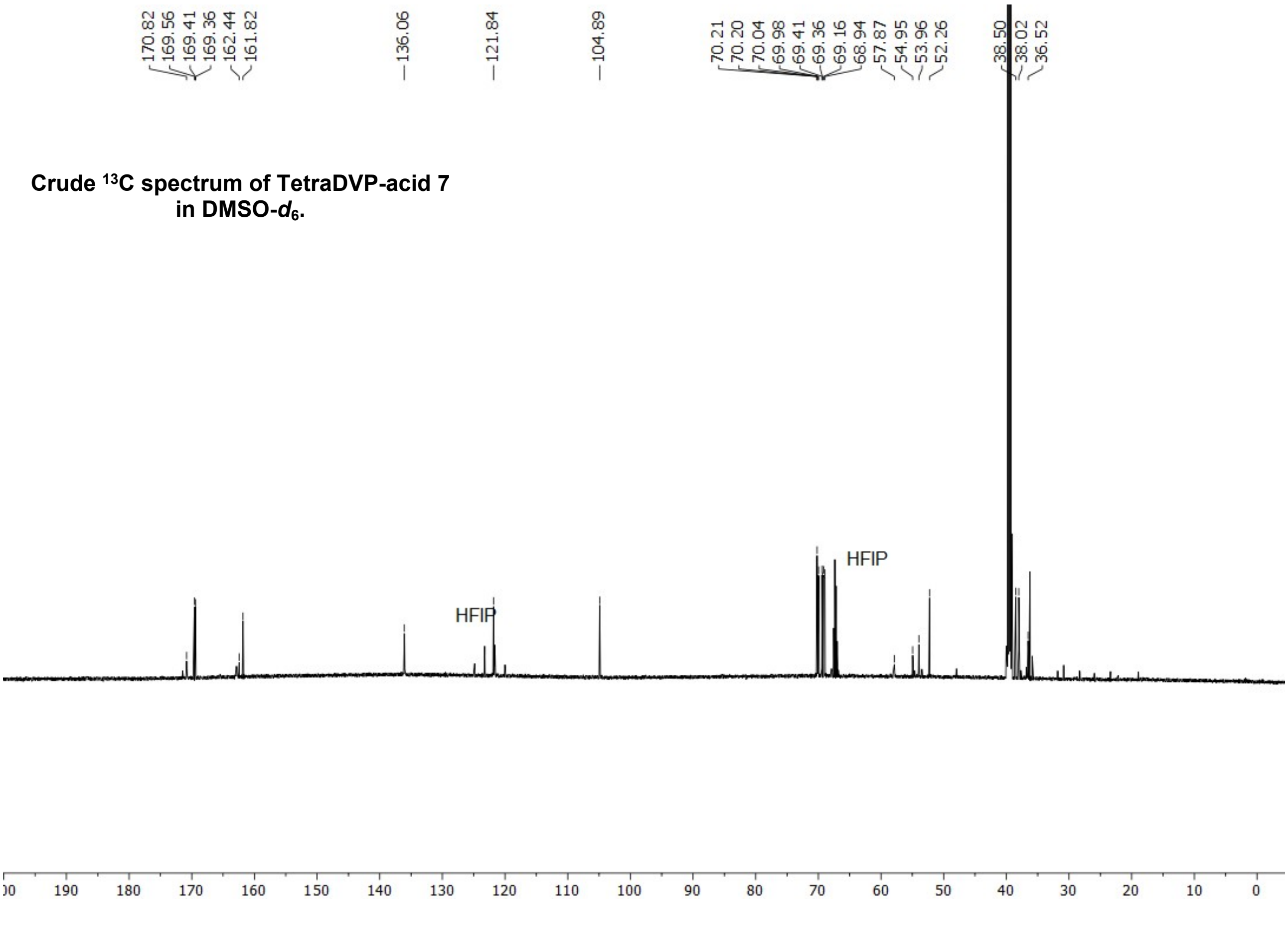

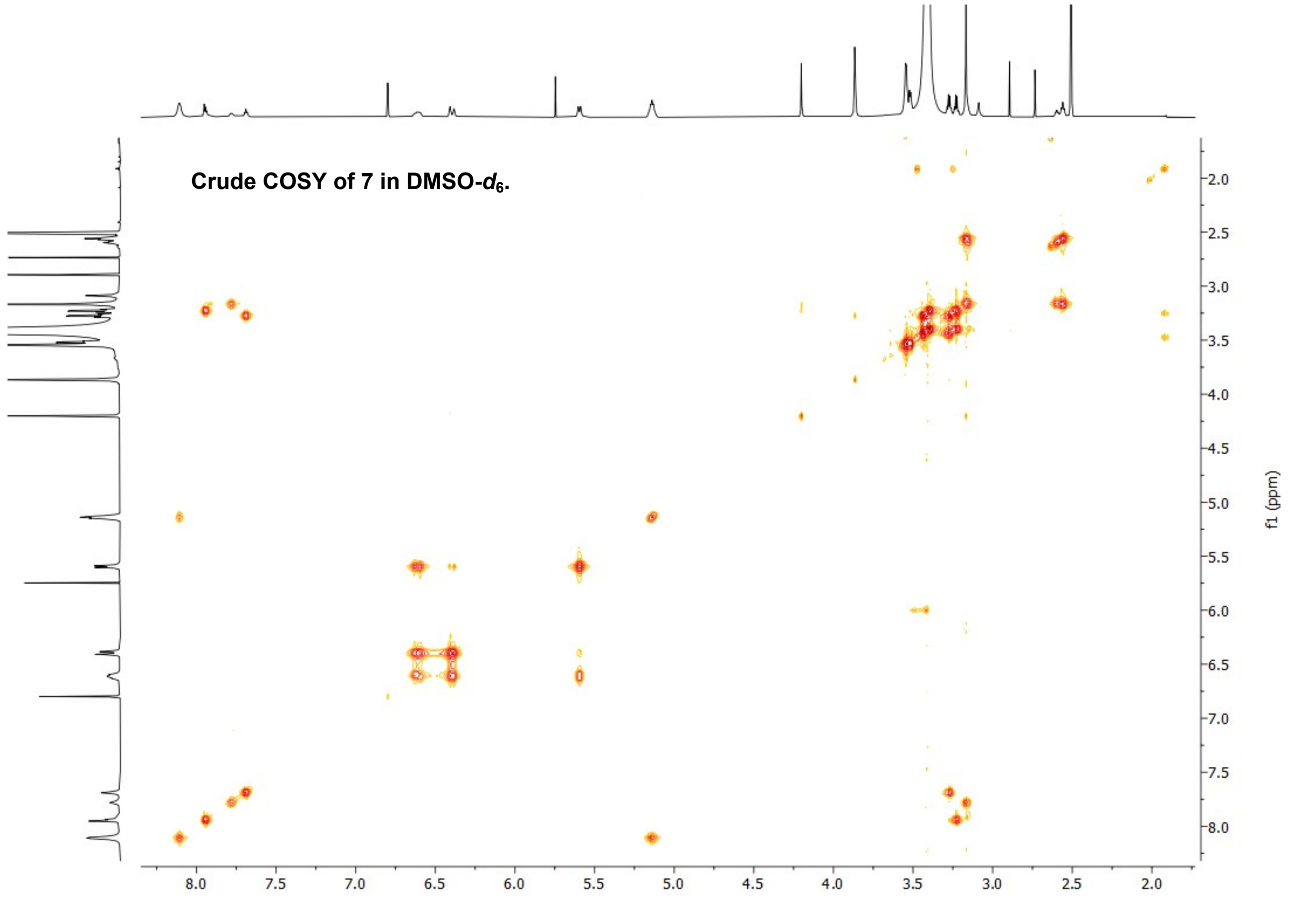

Crude COSY of 7 in DMSO-d_6.
f1 (ppm)

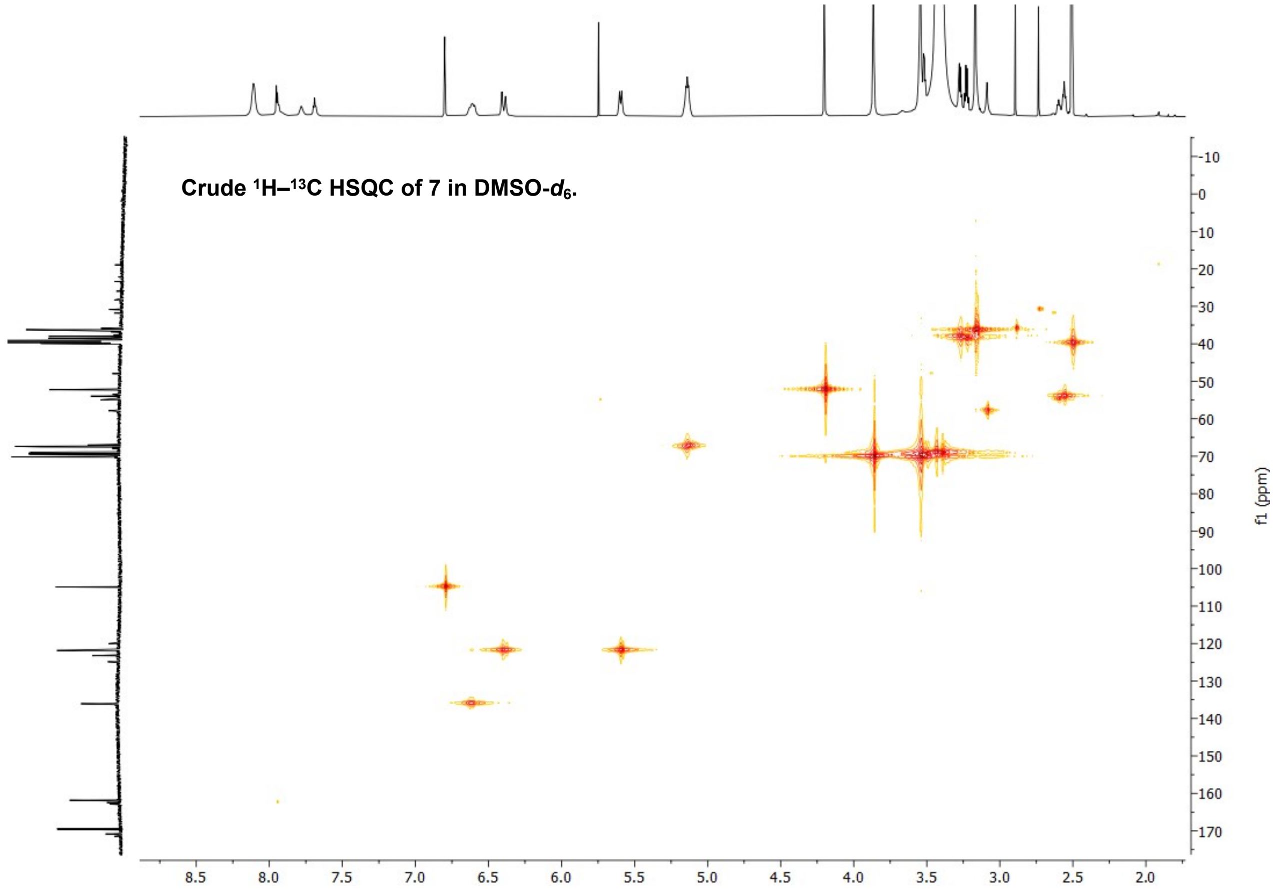

Crude ^1H–^{13}C HSQC of 7 in DMSO-d_6.
f1 (ppm)

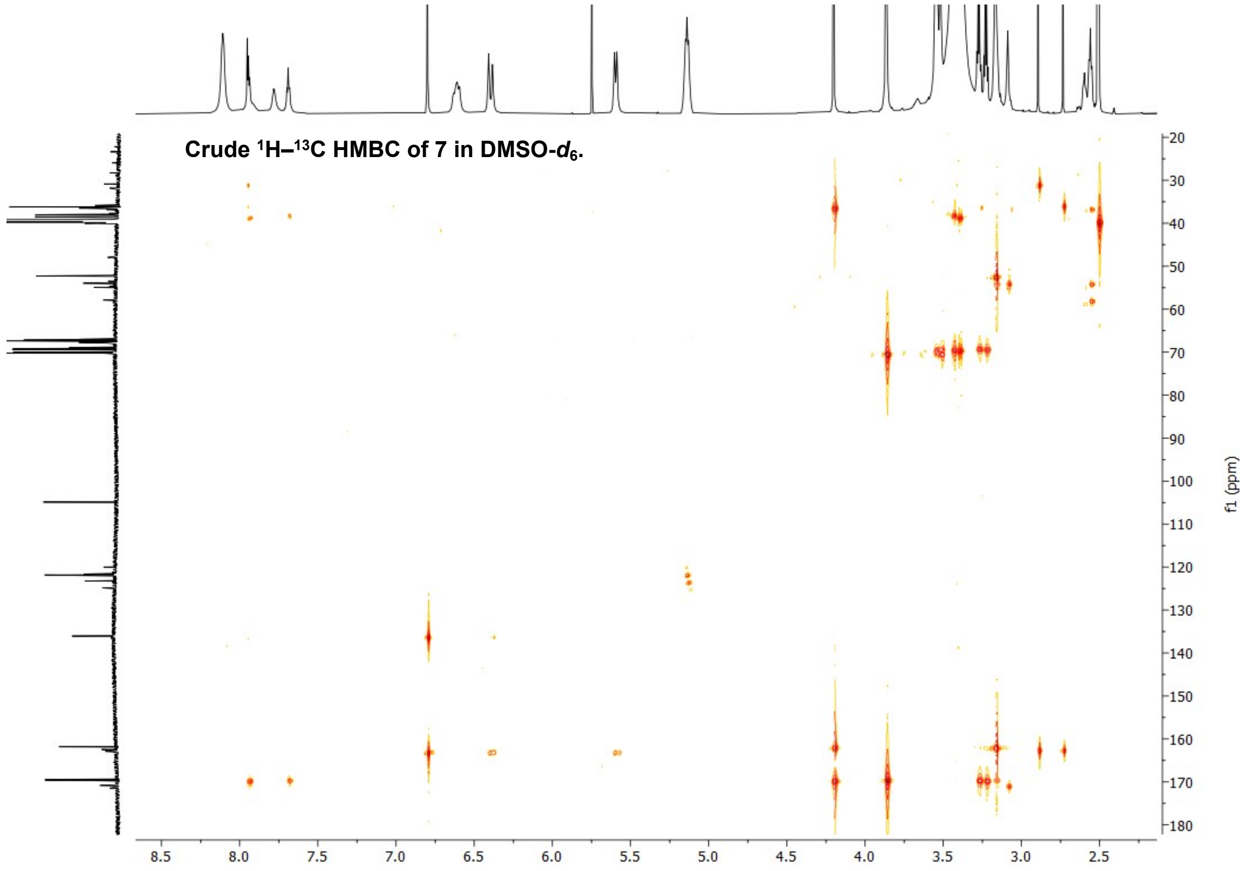

Crude 1H–13C HMBC of 7 in DMSO-d6.
f1 (ppm)

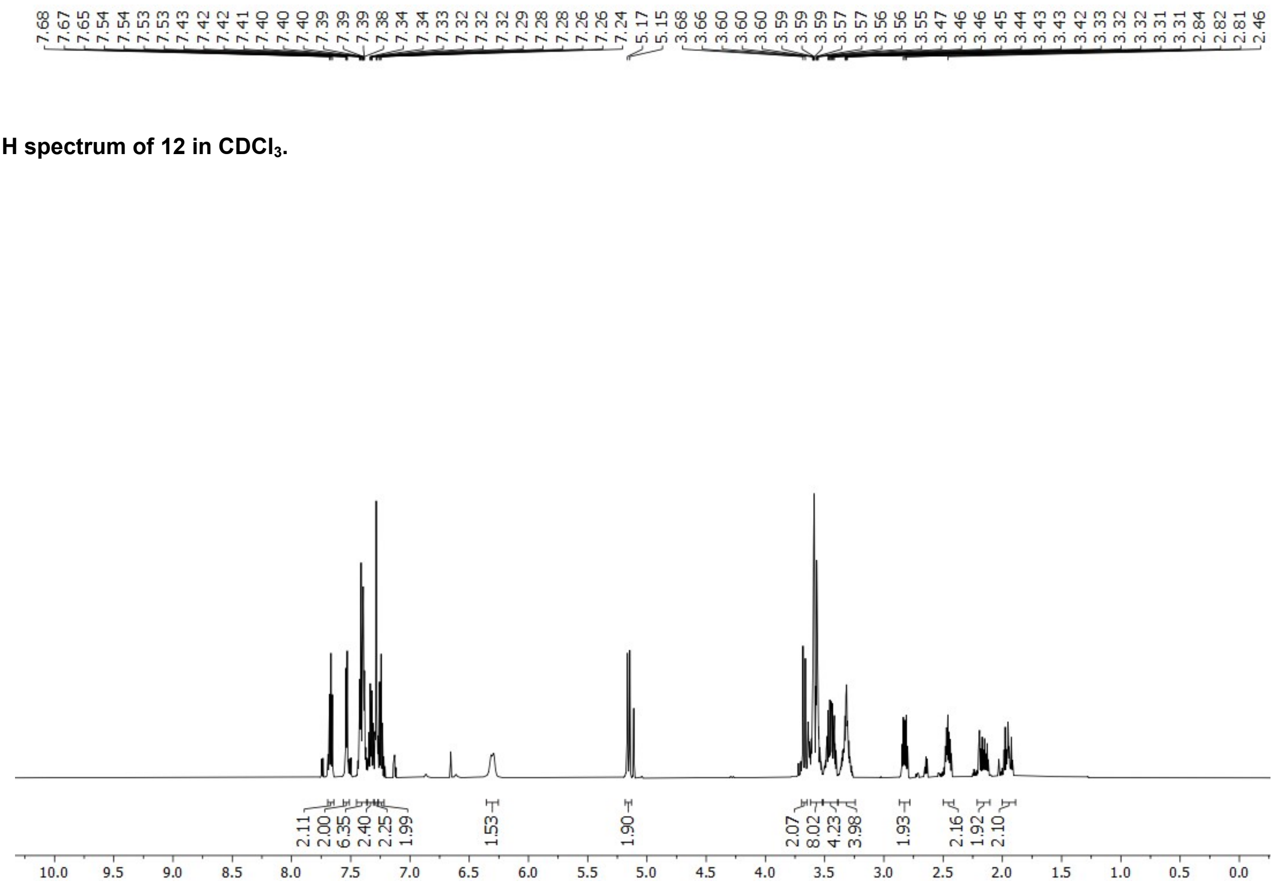

^{1}H spectrum of 12 in $CDCl_3$.

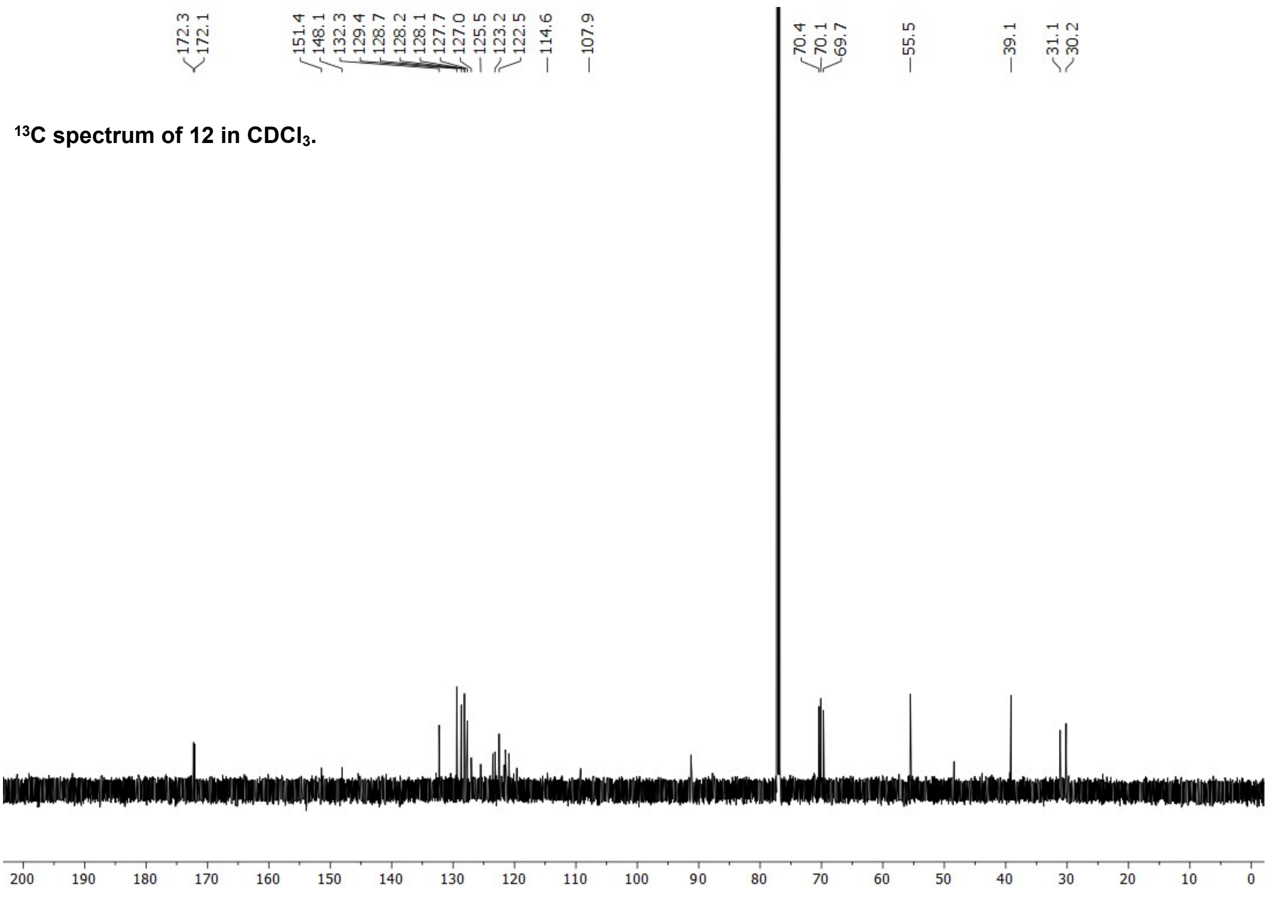

^{13}C spectrum of 12 in $CDCl_3$.

7. References

1 J. S. Parker, H. F. Sore, D. R. Spring, S. J. Walsh, *Linkers and Conjugates*, **2020**, WO2020025108A1.
2 D. R. Spring, S. J. Walsh, F. M. Dannheim, *Conjugating Reagents and Conjugates Thereof*, **2023**, WO2023006782A1.
3 S. A. McNelles, J. L. Pantaleo and A. Adronov, *Org. Process Res. Dev.*, 2019, **23**, 2740–2745.
4 A. H. Keeble, P. Turkki, S. Stokes, I. N. A. Khairil Anuar, R. Rahikainen, V. P. Hytonen and M. Howarth, *Proc. Natl. Acad. Sci.*, 2019, **116**, 26523–26533.
5 C. L. Driscoll, A. H. Keeble and M. R. Howarth, *Nat. Commun.*, 2024, **15**, 2403.
